# A randomized study of apalutamide in Chinese patients with non-metastatic castration-resistant prostate cancer

**DOI:** 10.1016/j.isci.2025.113166

**Published:** 2025-07-18

**Authors:** Shusuan Jiang, Ye Tian, Hongqian Guo, Jianming Guo, Haiying Dong, Hong Luo, Wei Xue, Tao Xu, Lei Li, Mingxing Qiu, Liping Xie, Angela Lopez-Gitlitz, Sharon McCarthy, Yanmei Liu, Haocheng Ma, Hongchuan Liang, Yanhui Li, Na Chen, Dingwei Ye

**Affiliations:** 1 Department of Urology, Hunan Cancer Hospital, Changsha, Hunan, China; 2 Department of Urology, Beijing Friendship Hospital, Beijing, China; 3 Department of Urology, Nanjing Drum Tower Hospital, Nanjing, Jiangsu, China; 4 Department of Urology, Zhongshan Hospital, Fudan University, Shanghai, China; 5 Department of Urology, Zhejiang Provincial People’s Hospital, Hangzhou, Zhejiang, China; 6 Department of Urology, Chongqing University Cancer Hospital, Chongqing, China; 7 Department of Urology, Shanghai Jiaotong University School of Medicine Renji Hospital, Shanghai, China; 8 Department of Urology, Peking University People’s Hospital, Beijing, China; 9 Department of Urology, The First Affiliated Hospital of Xi’an Jiaotong University, Xi’ an, Shaanxi, China; 10 Department of Urology, Sichuan Provincial People’s Hospital, Chengdu, Sichuan, China; 11 Department of Urology, The First Affiliated Hospital Zhejiang University College of Medicine, Hangzhou, Zhejiang, China; 12 Clinical Development, Johnson & Johnson, Los Angeles, CA, USA; 13 Clinical Development, Johnson & Johnson, Spring House, PA, USA; 14 Clinical Development, Johnson & Johnson, Beijing, China; 15 Statistics and Decision Sciences, Johnson & Johnson, Shanghai, China; 16 Medical Affairs Delivery Unit, Johnson & Johnson, Beijing, China; 17 Medical Affairs, Xi’an Janssen Pharmaceutical Ltd., Beijing, China; 18 Department of Urology, Fudan University Shanghai Cancer Center, Shanghai, China

**Keywords:** Oncology, Clinical medicine, Therapeutics

## Abstract

This post-approval commitment study addressed the limited data on the safety and efficacy of apalutamide in Chinese patients with non-metastatic castration-resistant prostate cancer (NM-CRPC). Utilizing a double-blinded, placebo-controlled trial with pre-planned crossover design, 75 patients were randomized (2:1) to receive apalutamide 240 mg daily or placebo while continuing androgen deprivation therapy. Apalutamide significantly reduced the risk of prostate-specific antigen (PSA) progression by 76.7% compared with placebo (hazard ratio [HR] = 0.233, *p* = 0.0052), with confirmed PSA response rate of 92.0% versus 12.0%. The median metastasis-free survival with apalutamide was 36.8 months, while the median overall survival was not reached. Grade 3 and 4 treatment-emergent adverse events were reported in 43.1% and 4.2% of patients, respectively, with hypertension, pneumonia, and rash being the most frequently reported, and the safety profile was consistent with existing data on apalutamide. Overall, these findings indicate that apalutamide is both efficacious and safe for Chinese patients, providing a valuable treatment option for high-risk NM-CRPC.

## Highlights

• Apalutamide+ADT reduces the risk of PSA progression in Chinese patients with NM-CRPC
• The combination shows consistent long-term benefit in Chinese patients with NM-CRPC
• The combination therapy has a tolerable safety profile in Chinese patients

## Graphical abstract

**Figure:**
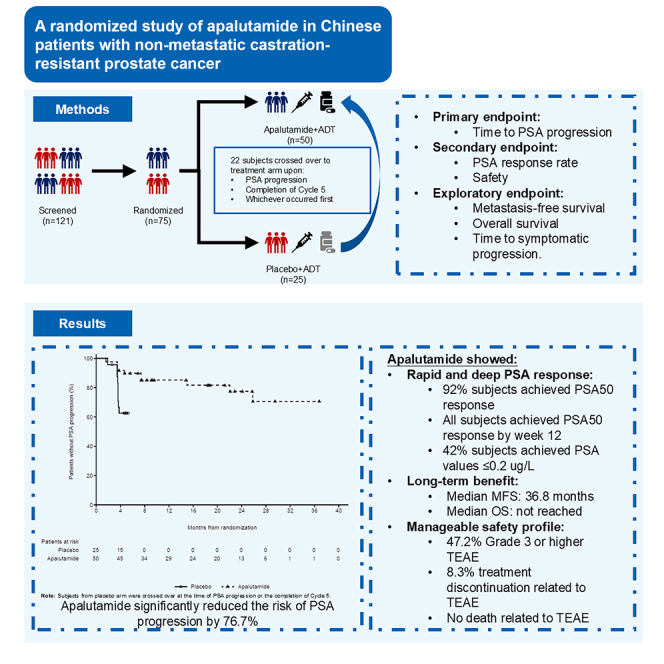

## Introduction

Prostate cancer (PC) ranks the sixth most common cancer among men in China, with approximately 125,646 new cases and 56,239 deaths estimated in 2022.^1^ In comparison with Western countries, where the estimated 5-year survival rate of PC exceeds 90%, China reports a lower rate of 69%.^2^ Initial treatment options for PC depend on factors such as disease stage, risk group, and overall health condition of the patient, including life expectancy.^3^

Approximately 30% of patients with localized PC treated with radical prostatectomy develop biochemical recurrence within 10 years.^4^ Androgen deprivation therapy (ADT) with or without an anti-androgen agent may be given to those patients with rising prostate-specific antigen (PSA) levels to inhibit the signaling of the androgen receptor, which is critical for tumor growth and progression.^5^ While a decline in PSA is usually observed following the initiation of ADT, most patients eventually experience rising PSA levels again with tumor regrowth, manifesting as castration-resistant PC (CRPC).^6^ When PSA levels rise without any evidence of metastatic disease detected through conventional imaging while the patient continues to receive ADT, the disease stage is categorized as non-metastatic CRPC (NM-CRPC).^7^ Without further treatment, high-risk NM-CRPC can rapidly metastasize, and the median overall survival (OS) is <2 years in patients with metastatic CRPC.^8^^,^^9^ As such, the main treatment goals for high-risk NM-CRPC are to delay the onset of metastasis and extend OS.^10^

Apalutamide (ERLEADA) is an oral, potent, next-generation selective androgen receptor pathway inhibitor (ARPI) that effectively inhibits the action of androgens, nuclear translocation of the androgen receptor, and DNA binding to androgen response elements.^11^^,^^12^^,^^13^ Apalutamide has been approved for the treatment of NM-CRPC and metastatic hormone-sensitive prostate cancer in multiple countries/territories.^14^^,^^15^ In the pivotal phase 3 trial (SPARTAN) evaluating apalutamide in patients with NM-CRPC, the addition of apalutamide to ADT demonstrated superior median metastasis-free survival (MFS) (40.5 vs. 16.2 months at the first interim analysis)^16^ and improved median OS (73.9 vs. 59.9 months at final analysis) compared with placebo.^17^ Despite 19% of patients in the placebo group crossing over to receive apalutamide and 84% of placebo-treated patients receiving subsequent therapy for metastatic PC, the apalutamide group showed a 22% reduction in the hazard of death.^17^

Apalutamide is one of the preferred treatment regimens recommended by guidelines for high-risk NM-CRPC.^3^ However, limited data are available regarding its use in the Chinese population. Therefore, this study was conducted as a post-approval commitment study to provide safety and efficacy data for apalutamide in Chinese patients with high-risk NM-CRPC.

## Results

### Patients

**Figure 1:**
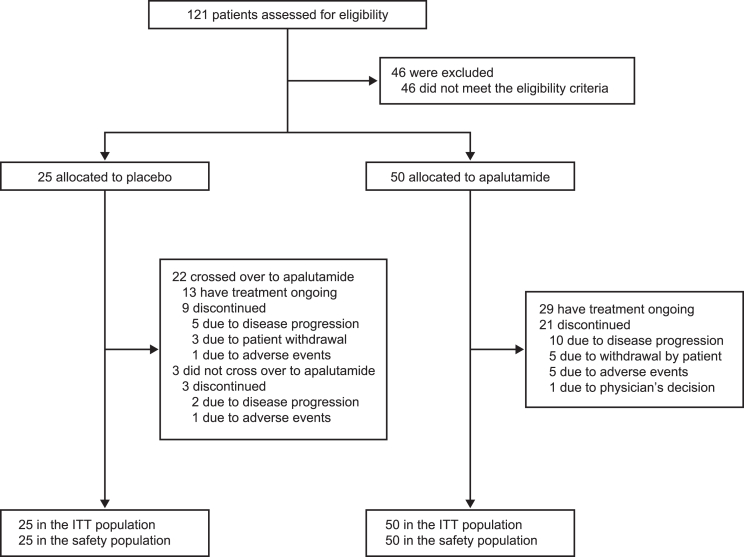
Trial profile

Of 121 patients who were screened for eligibility between 4 February 2020 and 16 December 2022, 75 were randomized (Figure 1). All enrolled patients received at least one dose of study drug.

The baseline demographics and characteristics were generally balanced between the two treatment arms (Table 1). The median age was 77.0 (range 56–93) years, with 43 patients (57.3%) aged ≥75 years. Forty patients (53.3%) had an Eastern Cooperative Oncology Group performance status (ECOG PS) of 0. Fifty-one patients (68.9%) had a Gleason score >7 at initial diagnosis. Most patients had a very high risk for the development of metastases (*n =* 65 [86.7%]), with a prostate-specific antigen doubling time (PSADT) ≤6 months. Fifty-six patients (74.7%) received prior surgery or radiotherapy for PC. All patients had received prior ADT: 71 patients (94.7%) were given gonadotropin-releasing hormone agonist (GnRHa) and four patients (5.3%) received orchiectomy. Sixty-seven patients (89.3%) received prior first-generation anti-androgen therapy.

**Table 1:** Demographic and baseline characteristics of the ITT population

	Placebo (*n =* 25)	Apalutamide (*n =* 50)	Total (*n =* 75)
Median age, years (range)	78.0 (58–85)	76.0 (56–93)	77.0 (56–93)
Age groups, *n* (%)			
<65 years	4 (16.0%)	6 (12.0%)	10 (13.3%)
≥65 to <75 years	5 (20.0%)	17 (34.0%)	22 (29.3%)
≥75 years	16 (64.0%)	27 (54.0%)	43 (57.3%)
Male	25 (100.0%)	50 (100.0%)	75 (100.0%)
Asian	25 (100.0%)	50 (100.0%)	75 (100.0%)
ECOG performance status score at enrollment, *n* (%)			
0	17 (68.0%)	23 (46.0%)	40 (53.3%)
1	8 (32.0%)	27 (54.0%)	35 (46.7%)
Median time from initial diagnosis to randomization, years (range)	3.4 (0.7–15.8)	4.9 (0.0–14.0)	4.7 (0.0–15.8)
Tumor stage at diagnosis, *n* (%)			
T1	3 (12.0%)	2 (4.0%)	5 (6.7%)
T2	5 (20.0%)	19 (38.0%)	24 (32.0%)
T3	9 (36.0%)	13 (26.0%)	22 (29.3%)
T4	5 (20.0%)	6 (12.0%)	11 (14.7%)
TX	3 (12.0%)	10 (20.0%)	13 (17.3%)
Lymph node stage at diagnosis, *n* (%)			
N0	17 (68.0%)	39 (78.0%)	56 (74.7%)
N1	5 (20.0%)	10 (20.0%)	15 (20.0%)
NX	3 (12.0%)	1 (2.0%)	4 (5.3%)
Metastasis stage at diagnosis			
M0	25 (100.0%)	50 (100.0%)	75 (100.0%)
Gleason score at diagnosis, *n* (%)			
<7	0	3 (6.1%)	3 (4.1%)
7	8 (32.0%)	12 (24.5%)	20 (27.0%)
3 + 4	3 (12.0%)	2 (4.1%)	5 (6.8%)
4 + 3	5 (20.0%)	10 (20.4%)	15 (20.3%)
>7	17 (68.0%)	34 (69.4%)	51 (68.9%)
Median PSADT, months (range)	3.2 (0.8–9.4)	3.1 (0.8–8.7)	3.2 (0.8–9.4)
PSADT groups, *n* (%)			
≤6 months	21 (84.0%)	44 (88.0%)	65 (86.7%)
>6 months	4 (16.0%)	6 (12.0%)	10 (13.3%)
Median PSA level at baseline, μg/L (range)	5.6 (1.9–69.0)	4.2 (0.5–69.7)	4.6 (0.5–69.7)
Prior prostate cancer therapy, *n* (%)			
Surgery or radiotherapy	15 (60.0%)	41 (82.0%)	56 (74.7%)
Surgery only	13 (52.0%)	31 (62.0%)	44 (58.7%)
Radiotherapy only	1 (4.0%)	2 (4.0%)	3 (4.0%)
Both surgery and radiotherapy	1 (4.0%)	8 (16.0%)	9 (12.0%)
Hormonal therapy	25 (100.0%)	50 (100.0%)	75 (100.0%)
GnRHa	22 (88.0%)	49 (98.0%)	71 (94.7%)
First generation anti-androgen	24 (96.0%)	43 (86.0%)	67 (89.3%)
Orchiectomy	2 (8.0%)	1 (2.0%)	3 (4.0%)
Other	1 (4.0%)	0	1 (1.3%)
Chemotherapy	2 (8.0%)	3 (6.0%)	5 (6.7%)
Other	0	1 (2.0%)	1 (1.3%)

ECOG, Eastern Cooperative Oncology Group; GnRHa, gonadotropin-releasing hormone agonist; ITT, intent-to-treat; PSA, prostate-specific antigen; PSADT, prostate-specific antigen doubling time.

At the time of data cutoff (1 June 2023), among 22 patients who switched to apalutamide from placebo, 13 remained on treatment, while among those originally assigned to apalutamide, 29 were still undergoing treatment. Disease progression (*n =* 17 [22.7%]) was the most common reason for treatment discontinuation; two discontinued during placebo treatment and 15 during apalutamide treatment. Eight patients (10.7%) discontinued due to their own decision, all during apalutamide treatment. The median duration of placebo exposure was 4.6 (range 3.4–4.9) months. The median duration of apalutamide exposure was 17.3 (range 1.2–29.1) months among patients who switched from placebo, and 20.0 (range 3.2–37.3) months among patients were originally assigned to the apalutamide arm.

### Efficacy

**Figure 2:**
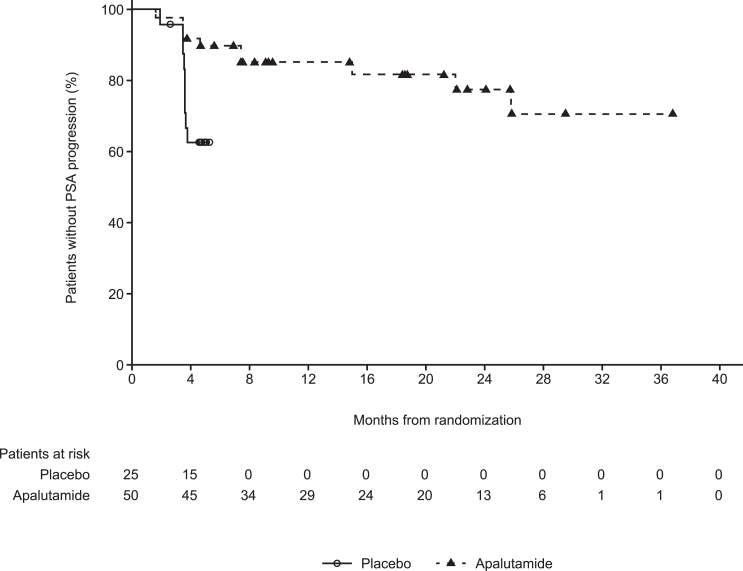
Kaplan-Meier plot of time to PSA progression of the ITT population ITT, intent-to-treat; PSA, prostate-specific antigen. Patients in the placebo arm who were event-free when switched to apalutamide were censored on the date of treatment crossover.

PSA progression was observed in 19 patients: 10 (20.0%) in the apalutamide arm and nine (36.0%) in the placebo arm. Treatment with apalutamide statistically significantly decreased the risk of PSA progression compared with placebo (hazard ratio [HR] 0.233 [95% confidence interval [CI]: 0.077–0.705], *p =* 0.0052) (Figure 2). The median time to PSA progression was not reached in either arm (Table 2). The benefit of apalutamide compared with placebo in time to PSA progression (TTPP) was consistently favorable across pre-specified subgroups, including age, number of prior hormone therapies, baseline ECOG PS, and PSADT at study entry (Figure S1).

**Table 2:** Summary of PSA results of the ITT population

	Placebo (*n =* 25)	Apalutamide (*n =* 50)
Median time to PSA progression, months (IQR)	NE (3.6–NE)	NE (25.8–NE)
PSA response rate (≥50% PSA reduction), *n* (%)	3 (12.0%)	46 (92.0%)
PSA response rate (≥50% PSA reduction) by week 12, *n* (%)	3 (12.0%)	46 (92.0%)
Deep PSA response (≥90% PSA reduction) by week 12, *n* (%)	1 (4.0%)	31 (62.0%)
Deep PSA response (PSA ≤0.2 μg/L) by week 12, *n* (%)	0	21 (42.0%)
Median PSA level at week 12^a^, μg/L (range)	5.4 (0.5–39.8)	0.3 (0.0–16.1)
Median maximum percent change^b^ in PSA level from baseline on or before cycle 6 days 1	3.80 (−99.3 to 44.1)	−96.10 (−99.8 to 43.1)
≥30% decline, *n* (%)	6 (24.0%)	48 (96.0%)
≥50% decline, *n* (%)	4 (16.0%)	48 (96.0%)
≥90% decline, *n* (%)	3 (12.0%)	35 (70.0%)
Median maximum percent change^b^ in PSA level from baseline at any time during study, % (range)	NC^c^	−97.33 (−99.9 to 43.1)
≥30% decline, *n* (%)	NC	48 (96.0%)
≥50% decline, *n* (%)	NC	48 (96.0%)
≥90% decline, *n* (%)	NC	37 (74.0%)

IQR, interquartile range; ITT, intent-to-treat; NC, not calculated; NE, not estimable; PSA, prostate-specific antigen.

a For patients who discontinued study treatment before week 12, the last PSA results before end of treatment was included.

b A negative percent indicates a decline in PSA, whereas a positive percent indicates that the patient never had a decline in PSA.

c Maximum percent change in PSA level from baseline at any time during study was not calculated for the placebo arm because most patients switched to apalutamide treatment during the study.

Compared with the placebo arm, the overall PSA response rate improved approximately 8-fold in the apalutamide arm (92.0% vs. 12.0%; *p <* 0.0001). The PSA response rate by week 12 showed the same results as the overall PSA response rate. Additionally, by cycle 6 days 1, a higher percentage of patients (96.0%) in the apalutamide arm experienced a ≥50% maximal decline in PSA from baseline compared with the placebo arm (16.0%). For PSA deep response by week 12, the apalutamide arm also had a higher percentage of patients with a ≥90% reduction in PSA (62.0% vs. 4.0%) and a greater proportion with PSA values ≤0.2 μg/L (42.0% vs. none) compared with the placebo arm. At week 12, PSA levels decreased in the apalutamide arm (median change of 94.53% decrease from baseline) but increased in the placebo arm (28.11% increase from baseline).

As of the data cutoff date, the median follow-up duration was 21.8 months. The median MFS in the apalutamide arm was estimated as 36.8 months (95% CI: 26.6–not reached), with 12 patients (24.0%) who had either detectable bone or soft-tissue distant metastasis or had died. The event-free rates at 12, 24, and 36 months were 90.1%, 75.1%, and 53.1%, respectively (Figure S2). The median OS was not reached, and there were four recorded deaths (Figure S3). No patients in the apalutamide arm experienced symptomatic progression events.

### Safety

A summary of the treatment-emergent adverse events (TEAEs) in the placebo arm, apalutamide arm, and apalutamide-treated patients is shown in Table 3. Due to the early crossover nature of the study design, median exposure to the assigned treatment based on randomization was longer in the apalutamide arm compared with the placebo arm (20.0 vs. 4.6 months). Therefore, direct comparison of AE incidence between the two arms was not relevant. Instead, the following safety profile was focused on all patients who received at least one dose of apalutamide (apalutamide-treated patients, *n =* 72).

**Table 3:** Summary of TEAEs of the safety population

	Placebo^a^ (*n =* 25)	Apalutamide (*n =* 50)	Apalutamide- treated^b^ (*n =* 72)
At least one TEAE	18 (72.0%)	48 (96.0%)	70 (97.2%)
Grade 3–4 TEAEs	3 (12.0%)	25 (50.0%)	34 (47.2%)
TEAEs leading to treatment discontinuation	1 (4.0%)	5 (10.0%)	6 (8.3%)
TEAEs leading to death	1 (4.0%)	2 (4.0%)	2 (2.8%)
TEAE with incidence ≥5%			
Anemia	3 (12.0%)	15 (30.0%)	20 (27.8%)
Rash	3 (12.0%)	14 (28.0%)	18 (25.0%)
Hypertension	2 (8.0%)	11 (22.0%)	14 (19.4%)
COVID-19	0	8 (16.0%)	13 (18.1%)
Hyperglycemia	0	10 (20.0%)	13 (18.1%)
Leukopenia	0	9 (18.0%)	13 (18.1%)
Weight decreased	0	10 (20.0%)	12 (16.7%)
Rib fracture	1 (4.0%)	5 (10.0%)	7 (9.7%)
Neutropenia	0	4 (8.0%)	6 (8.3%)
Pruritus	1 (4.0%)	5 (10.0%)	6 (8.3%)
Hypercholesterolemia	0	4 (8.0%)	5 (6.9%)
Hyperkalemia	0	5 (10.0%)	5 (6.9%)
Lymphopenia	1 (4.0%)	4 (8.0%)	5 (6.9%)
Pneumonia	1 (4.0%)	5 (10.0%)	5 (6.9%)
Alanine aminotransferase increased	1 (4.0%)	3 (6.0%)	4 (5.6%)
Eczema	0	3 (6.0%)	4 (5.6%)
Fatigue	0	3 (6.0%)	4 (5.6%)
Hypertriglyceridemia	1 (4.0%)	2 (4.0%)	4 (5.6%)
Hypoalbuminemia	0	3 (6.0%)	4 (5.6%)
Hypokalemia	1 (4.0%)	3 (6.0%)	4 (5.6%)
Malaise	0	4 (8.0%)	4 (5.6%)
Pain in extremity	0	3 (6.0%)	4 (5.6%)
Pyrexia	1 (4.0%)	0	4 (5.6%)
Toothache	0	2 (4.0%)	4 (5.6%)
Arthralgia	0	3 (6.0%)	3 (4.2%)
Aspartate aminotransferase increased	2 (8.0%)	2 (4.0%)	3 (4.2%)
Back pain	0	3 (6.0%)	3 (4.2%)
Blood thyroid-stimulating hormone increased	0	3 (6.0%)	3 (4.2%)
Diarrhea	0	3 (6.0%)	3 (4.2%)
Fracture	0	3 (6.0%)	3 (4.2%)
Low-density lipoprotein increased	0	3 (6.0%)	3 (4.2%)
Edema peripheral	0	3 (6.0%)	3 (4.2%)
Rash maculo-papular	0	3 (6.0%)	3 (4.2%)

Data are *n* (%) or *n*. ADT, androgen deprivation therapy; TEAE, treatment-emergent adverse event.

a Patients who were treated with placebo plus ADT before crossover.

b Patients who were treated with apalutamide plus ADT in the study; included patients from the apalutamide arm and patients switched from the placebo arm.

Discontinuation of apalutamide due to one or more TEAEs occurred in six patients (8.3%), including rash (three patients [4.2%]), pneumonia (two patients [2.8%]), sepsis (one patient [1.4%]), skin ulcer (one patient [1.4%]), and leukopenia (one patient [1.4%]). Dose modification due to TEAEs occurred in 24 patients (33.3%); most frequently reported were rash (six patients [8.3%]), pneumonia (three patients [4.2%]), dermatitis allergic (two patients [2.8%]), eczema (two patients [2.8%]), and COVID-19 (two patients [2.8%]). Three deaths occurred within 30 days of the last dose of study drug, including one patient in the placebo arm due to suicide and two apalutamide-treated patients due to pneumonia; all deaths were deemed unrelated to apalutamide treatment.

Almost all (*n =* 70 [97.2%]) patients experienced at least one TEAE. The most common TEAEs with an incidence of ≥15% were anemia (20 patients [27.8%]), rash (18 patients [25.0%]), hypertension (14 patients [19.4%]), COVID-19 (13 patients [18.1%]), hyperglycemia (13 patients [18.1%]), leukopenia (13 patients [18.1%]), and weight decreased (12 patients [16.7%]). Grade 3 and 4 TEAEs were observed in 31 (43.1%) and three (4.2%) patients, while hypertension (seven patients [9.7%]), pneumonia (four patients [5.6%]), and rash (four patients [5.6%]) were most frequently reported (incidence ≥3%). Of note, the grade 3 hypertension by Common Terminology Criteria for Adverse Events (CTCAE) criteria is clinical stage 2 hypertension, which can be managed with additional medication. Rash grading by CTCAE criteria is evaluated by body surface area covered by rash rather than severity of rash.^18^ Grade 4 leukopenia, neutropenia, hyperkalemia, sepsis, and atrioventricular block complete occurred once each (1.4%). Treatment-related AEs (TRAEs) that were considered by the investigators to be related to apalutamide occurred in 55 patients (76.4%); the most frequently reported (incidence ≥10%) TRAEs were rash (18 patients [25.0%]), hyperglycemia (10 patients [13.9%]), anemia (nine patients [12.5%]), hypertension (nine patients [12.5%]), and leukopenia (eight patients [11.1%]). Of 10 patients who experienced hyperglycemia, two had pre-existing diabetes.

Half of the patients (50%) experienced AEs of special interest (AESIs), of which skin rash, fracture, and cerebrovascular disorders were reported in 27 (37.5%), 13 (18.1%), and four patients (5.6%), respectively. The other known AESIs of apalutamide were not observed during the study. Most skin rash events were grade 1 or 2 in severity, while grade 3 skin rash events were reported in six patients (8.3%). No Stevens-Johnson syndrome or toxic epidermal necrolysis cases were reported. Skin rash events were manageable with dose modification and supportive care.

## Discussion

This randomized, double-blinded, placebo-controlled phase 4 study met its primary endpoint by demonstrating that treatment with apalutamide achieved a statistically significant and clinically meaningful decrease in the risk of PSA progression. The treatment effect of apalutamide on TTPP across the different pre-specified subgroups (age, number of prior hormone therapies, baseline ECOG PS, and PSADT at study entry) was consistent with the overall population, which all favored the apalutamide arm. Furthermore, the apalutamide arm demonstrated superior PSA response and deep PSA response (PSA reduction ≥90% from baseline or PSA level ≤0.2 μg/L) by week 12. The safety profile of the apalutamide combination was consistent with observations in previous studies without new safety signals.^19^^,^^20^^,^^21^

Generally, patients in this study had more severe baseline disease characteristics compared with the SPARTAN population.^16^ The median PSADT at baseline was much shorter in this study than that in the SPARTAN study (unpublished data). A higher proportion of patients in this study had a Gleason score of >7 at diagnosis, N1 stage at diagnosis, and ECOG PS score of 1. Moreover, the median age of patients in this study was also slightly older than that in the SPARTAN study, with a higher proportion of patients aged ≥75 years old (unpublished data).

Although PSA-related endpoints rather than long-term survival outcomes were chosen as primary and secondary endpoints in this study, it is important to note that PSA monitoring has demonstrated prognostic value, as there is a strong association between early PSA response and long-term benefits such as MFS and OS.^22^ The efficacy of apalutamide in combination with ADT in Chinese patients with NM-CRPC was generally consistent with the global population findings from the SPARTAN study^16^ (HR for TTPP: 0.23 vs. 0.06; percentage of patients with a PSA response: 92.0% vs. 89.7%; median percentage decline in PSA at week 12: 94.5% vs. 89.7%), despite enrolling patients with more severe baseline disease characteristics. The relatively higher HR for TTPP observed in our study may be impacted by the smaller sample size (75 patients in this study vs. 1,207 patients in SPARTAN), as well as the crossover design employed. As the proportions of patients in the apalutamide arm who experienced PSA progression were similar between the two studies (20.0% in this study vs. 24.0% in SPARTAN study), the TTPP results were considered comparable (unpublished data).

In terms of long-term outcomes, the SPARTAN study showed superior MFS outcomes in the apalutamide arm over the placebo arm (22.8% vs. 48.4%). Unfortunately, it is not possible to directly compare the MFS and OS outcomes between both arms given the crossover design of the present study. Besides, as of the clinical cutoff, the MFS and OS of the present study remain immature. When comparing with historical data, long-term efficacy endpoints in this study showed clinically meaningful improvement when compared with historical data of ADT alone.^16^^,^^23^^,^^24^

Apalutamide in combination with ADT had a manageable safety profile in Chinese patients, which is consistent with the known safety profile reported in the SPARTAN and TITAN studies.^19^^,^^20^ The incidence of TEAEs (97.2% vs. 97.3% vs. 96.8%), grade 3–4 TEAEs (47.2% vs. 53.1% vs. 42.2%), and TEAEs leading to deaths (2.8% vs. 2.1% vs. 1.9%) were comparable among the current, SPARTAN, and TITAN studies. The most common TEAEs observed in this study were generally consistent with those reported in the SPARTAN and TITAN studies, with most TEAEs mild to moderate, and manageable with supportive care and dose modification. Of note, the rates of anemia, COVID-19, hyperglycemia, and leukopenia were higher in the present study than that of SPARTAN and TITAN. The higher incidence of cytopenia, especially the higher reporting rate of anemia, may be attributed to a more aggressive disease pattern, as 12% of the patients in the placebo arm also reported anemia prior to crossover. Meanwhile, when looking at the patient-level data, 80% of the anemia cases were grade 1 and 20% were grade 2, which were of mild to moderate severity and could be well managed.

An integrated analysis showed that the incidence of apalutamide-related skin rash was higher in the East Asian population compared with patients from the rest of the world.^25^ The incidence of any-grade (51.5%) and grade 3 (14.7%) skin rash in Japanese patients from the pooled analysis of the three studies was higher compared with the global populations of the SPARTAN (any grade: 23.8%; grade 3: 5.2%) and TITAN (any grade: 27.1%; grade 3: 6.3%) studies. Similarly, incidence of skin rash in our study (any grade: 37.5%; grade 3: 8.3%) showed a consistent trend where the incidences were comparable with the East Asian populations of the SPARTAN (any grade: 37.8%; grade 3: 12.2%) and TITAN studies (any grade: 37.3%; grade 3: 10.9%) but higher than the global population (unpublished data).^19^ Although the incidence of skin rash was higher in Chinese patients, most events were low grade (grade 1–2) and manageable through dose modification and/or supportive medications.

In conclusion, apalutamide in combination with ADT showed superior efficacy compared with ADT alone in Chinese patients with NM-CRPC. The safety profile of this combination in Chinese patients was generally comparable to the known adverse reactions in the overall population.

### Limitations of the study

This study had a few limitations. First, the sample size of the study was relatively small. Second, the median time to PSA progression in the placebo arm was not estimable. Moreover, due to the early crossover design (which was done to minimize the exposure of patients to less-effective treatment) and the relatively short follow-up at data cutoff (median 21.8 months), comparison of long-term outcomes (MFS and OS) could not be made. Additionally, the COVID-19 pandemic also affected the conduct of the study, leading to travel restrictions that posed challenges in obtaining samples for central evaluation of PSA. Fortunately, these protocol deviations had minimal impact on data quality.

## Resource availability

### Lead contact

• Requests for further information and resources should be directed to the lead contact, Dingwei Ye (dwyeli@163.com).

### Materials availability

• This study did not generate new unique reagents.

### Data code and availability

• Data: The full dataset was deposited. The data sharing policy of Johnson & Johnson is available at https://www.janssen.com/clinical-trials/transparency.
• Code: This paper does not report the original code.
• Any additional information required to reanalyze the data reported in this paper is available from the lead contact upon request.

## Acknowledgments

This study was supported by 10.13039/100004331Johnson & Johnson.

We thank the patients, investigators, and site staff for their participation in the study. We also thank the following employees from Johnson & Johnson: Xiangdan Wu for study administration, Yi Zhang for the writing of the clinical study report, Yu Cheng for statistical programming, Siyuan Yu for data management, Yuping Chen for statistical inputs on study design, Zhuokai Li and Ting Sun for performing the statistical analysis, and Xue Qi for clinical inputs on study design. Medical writing assistance was provided by Shao-Hua Chin and Lawrence Law from Parexel (funded by Johnson & Johnson).

## Author contributions

Conceptualization and design, A.L.-G., S.M., Y.L., H.L., and D.Y.; administrative support, Y.L.; provision of study material or patients, S.J., Y.T., H.G., J.G., H.D., H.L., W.X., T.X., L.L., M.Q., L.X., and D.Y.; collection and assembly of data, S.J., Y.T., H.G., J.G., H.D., H.L., W.X., T.X., L.L., M.Q., L.X., and D.Y.; unrestricted access to all data, A.L.-G., S.M., Y.L., H.M., and H.L.; data analysis, Y.L., H.M., H.L., and D.Y.; data interpretation, Y.L., H.M., H.L., N.C., S.J., Y.T., and D.Y.; preparation of the first draft of manuscript, all authors; final approval of manuscript: all authors. All authors agreed to submit the manuscript, read and approved the final draft, and take full responsibility of its content, including the accuracy of the data and the fidelity of the trial to the registered protocol and its statistical analysis.

## Declaration of interests

S.J., Y.T., H.G., J.G., H.D., H.L., W.X., T.X., L.L., M.Q., L.X., and D.Y. reported grants from Johnson & Johnson for the conduct of the study and medical writing support. A.L.-G., S.M., Y.L., and H.L. are current employees of Johnson & Johnson and hold stock in Johnson & Johnson. H.M. and Y.L. are current employees of Johnson & Johnson. N.C. is a current employee of Xi’an Janssen Pharmaceutical Ltd.

## STAR★Methods

### Key resources table

**Table:** 

REAGENT or RESOURCE	SOURCE	IDENTIFIER
**Biological samples**		

Blood from prostate cancer patients	Patients enrolled	NA

**Critical commercial assays**		

Prostate-specific antigen	Hybritech PSA reagent	Beckman Coulter Inc., Brea, CA
Testosterone	Testosterone reagent	Beckman Coulter Inc., Brea, CA
Direct bilirubin	Roche DBILI direct bilirubin reagent	Manufactured by Cobas and distributed by Roche Diagnostics, Indianapolis, IN
Total bilirubin	Roche bilirubin total Gen. 3 (BILTS V3) reagent	Manufactured by Cobas and distributed by Roche Diagnostics, Indianapolis, IN
ALT	Roche ALT reagent	Roche Diagnostics, Indianapolis, IN
AST	Roche AST reagent	Roche Diagnostics, Indianapolis, IN
Creatinine	Roche creatinine Jaffé Gen. 2 reagent	Roche Diagnostics
Fasting glucose	Roche glucose reagent	Roche Diagnostics
Serum sodium Serum potassium	Roche ISE indirect Na (sodium) reagent Roche ISE indirect K (potassium) reagent	Roche Diagnostics, Indianapolis, IN
Direct LDL 3rd-gen, fasting	Roche direct LDL 3rd-gen reagent	Roche Diagnostics
Hematology	CBC TIMEPAC&DIFF TIMEPAC	Siemens Medical Solutions Diagnostics, Inc., Tarrytown, NY
Hepatitis B Hepatitis B Core Total Hepatitis B core IGM	HbsAgII (HBsII) reagent HBc total (HBcT) reagent HBc IgM (HBcIgM) primary reagent	Siemens Healthcare Diagnostics, Tarrytown, NY
Hepatitis C virus antibody	HCV/aHCV reagent	Siemens Healthcare Diagnostics, Tarrytown, NY
Lipid panel	HDLC4 (HDL-cholesterol Gen. 4) reagent, triglycerides reagent	Roche Diagnostics
Thyroid-stimulating hormone	TSH (3rd IS) reagent	Beckman Coulter Access Immunoassay Systems
Free T4	Free T4 reagent	Beckman Coulter Inc., Fullerton, CA
HBV DNA AMPLIPREP TAQMAN 2.0	Cobas TaqMan HBV test v2.0	Roche Cobas
HCV RNA AMPLIPREP TAQMAN 2.0	Cobas TaqMan HCV test v2.0	Roche Cobas

**Deposited data**		

Full dataset	Life Science Analytics Frame	SAS Institute Inc. Identifier: DBR_FA_20230815_56021927PCR4007_RELEASE2

**Software and algorithms**		

SAS 9.4	SAS Institute Inc.	https://www.sas.com/
RTSM 4.0	Bracket Global LLC	https://rtsm.bracketglobal.com/

### Experimental model and study participant details

This was a randomized, double-blinded, placebo-controlled phase 4 study conducted at 24 hospitals in China. Adult male patients (≥18 years of age) with pathologically confirmed adenocarcinoma of the prostate without neuroendocrine differentiation or small cell features and a prostate-specific antigen (PSA) doubling time (PSADT) of ≤10 months, either medically (ongoing gonadotropin-releasing hormone agonist [GnRHa]) or surgically castrated, were enrolled. Patients were also required not to have received prior systemic anti–prostate cancer therapy, except for andogen deprivation therapy and first-generation anti-androgens, and had to have an Eastern Cooperative Oncology Group performance status of 0 or 1. Patients were excluded if they had distant metastases or a history of seizures. A complete list of inclusion and exclusion criteria is provided in the supplemental information.

This study was conducted in accordance with the principles of the Declaration of Helsinki, consistent with Good Clinical Practices, and applicable local regulatory requirements. All patients or their legally authorized representatives provided written, dated informed consent to participate in the study before enrollment. The study protocol and all amendments were approved by the independent ethics committee/institutional review board (Table S1) before implementation.

### Method details

#### Randomization and blinding

Patients were randomly assigned in a 2:1 ratio using an interactive web response system (IWRS) to receive either apalutamide or a matching placebo. The randomization process was stratified according to PSADT (>6 vs. ≤6 months). All patients who had not undergone prior bilateral orchiectomy continued to receive GnRHa. Apalutamide 240 mg or placebo daily was administered orally. At the time of study design, apalutamide had been approved for NM-CRPC treatment in China. Therefore, for ethical consideration, patients randomized to the placebo arm would switch to apalutamide treatment after completing five cycles of placebo or at the onset of PSA progression, whichever occurred first. Study treatment would continue until the development of distant metastases as assessed by investigators or the occurrence of adverse events (AEs) or withdrawal of consent. Dose modifications of apalutamide, as well as interventions for the management of local or regional symptoms, were allowed.

Patients, investigators, and study personnel were blinded to treatment assignment and PSA results until the final analysis of this study. To maintain treatment blinding, PSA results were analyzed by a central laboratory. For patients in the placebo arm who had PSA progression within the first 5 months of study treatment, an automatic switch to active treatment would be triggered through the IWRS.

#### Procedures

Participants' information on sex, age, and race was self-reported. Information on gender and socioeconomic status was not collected. PSA assessment was done no later than 1 week before day 1 of cycles 3, 4, and 5. Tumor imaging (computed tomography or magnetic resonance imaging) was done at baseline and every 16 weeks until investigator-assessed disease progression. Patients who discontinued study treatment before documented disease progression continued with tumor assessments as planned. Safety was monitored from the time informed consent was obtained up to 30 days after the last dose of the study intervention. AEs were recorded and graded for severity using Common Terminology Criteria for Adverse Events (CTCAE) version 4.03. Grouped terms for AEs of special interest (AESIs) for apalutamide included skin rash (a grouped term including several skin-related abnormalities), fracture, cerebrovascular disorders, seizure, fall, and ischemic heart disease.

#### Endpoints and assessment

The primary efficacy endpoint of this study was time to PSA progression (TTPP), which was defined as the time from randomization to the first date of documented PSA progression based on the PC Working Group 2 (PCWG2) criteria.^26^ The secondary endpoints included PSA response rate and safety. The exploratory endpoints were metastasis-free survival (MFS), overall survival (OS), and time to symptomatic progression. The PSA response rate was defined as the proportion of patients who achieved at least a 50% decline in PSA value from baseline, as assessed by a central laboratory following PCWG2 criteria. A deep PSA response was defined as at least a 90% decline in PSA value from baseline or a PSA value ≤0.2 μg/L. The PSA response had to be confirmed by a central laboratory measurement obtained ≥4 weeks later. MFS was defined as the time from randomization to first evidence of investigator-assessed radiographically detectable bone or soft tissue distant metastasis using the Response Evaluation Criteria in Solid Tumors (RECIST v1.1) or death due to any cause (whichever occurred earlier). Time to symptomatic progression was defined as the time from randomization to first documentation of a skeletal-related event (pathologic fracture, spinal cord compression, or need for surgical intervention or radiation therapy to the bone), pain progression or worsening of disease-related symptoms requiring initiation of a new systemic anti-cancer therapy, or development of clinically significant symptoms due to loco-regional tumor progression requiring surgical intervention or radiation therapy. Safety outcomes, which were AEs, were monitored from the time informed consent was obtained until up to 30 days after the last dose of the study intervention.

### Quantification and statistical analysis

The study aimed to enroll 75 patients, providing approximately 95% power to detect a hazard ratio (HR) of 0.11 for TTPP at a two-tailed significance level of 0.05.

Efficacy was analyzed in the intent-to-treat (ITT) population, which included all patients randomized into the study. Safety was assessed in the safety population which included all patients who received at least one dose of study drug.

A two-sided significance level of 0.05 was used for all hypothesis testing, and two-sided 95% confidence intervals (CIs) were calculated. The pre-specified analysis plan included a stratified analysis based on PSADT (>6 vs. ≤6 months) for the primary endpoint. However, due to a limited number of events for TTPP in each PSADT stratum in both treatment arms, non-stratified analyses were conducted under the pre-specified pooling algorithm. For the primary endpoint, a Cox proportional-hazard model was used to estimate the HR and its 95% CI. Comparison between the two treatment arms was based on a non-stratified log-rank test. Time-to-event endpoints were summarized using the Kaplan-Meier method. The PSA response rate was summarized descriptively. The relative risk (treatment: control) was reported along with the associated two-tailed 95% CIs, and Fisher’s exact test was used to compare the two arms.

### Additional resources

The study was registered at clinicaltrials.gov (NCT04108208).

## Supplemental information

Document S1. Figures S1–S3, Table S1, and Method S1

Data S1. CONSORT checklist, study protocol, and study statistical analysis plan
