# Supplementary material for: A randomized study of apalutamide in Chinese patients with non-metastatic castration-resistant prostate cancer
Source: iScience. 2025 Jul 18;28(8):113166. doi: 10.1016/j.isci.2025.113166 (PMC12355417; doi:10.1016/j.isci.2025.113166)
Supplement: Document S1. Figures S1–S3, Table S1, and Method S1 [file mmc1.pdf]

iScience, Volume *28*

Supplemental information

A randomized study of apalutamide in Chinese patients with non-metastatic castration-resistant prostate cancer

Shusuan Jiang, Ye Tian, Hongqian Guo, Jianming Guo, Haiying Dong, Hong Luo, Wei Xue, Tao Xu, Lei Li, Mingxing Qiu, Liping Xie, Angela Lopez-Gitlitz, Sharon McCarthy, Yanmei Liu, Haocheng Ma, Hongchuan Liang, Yanhui Li, Na Chen, and Dingwei Ye

Figure S1. Forest plot of time to PSA progression for subgroups defined by baseline clinical disease characteristics (ITT population)

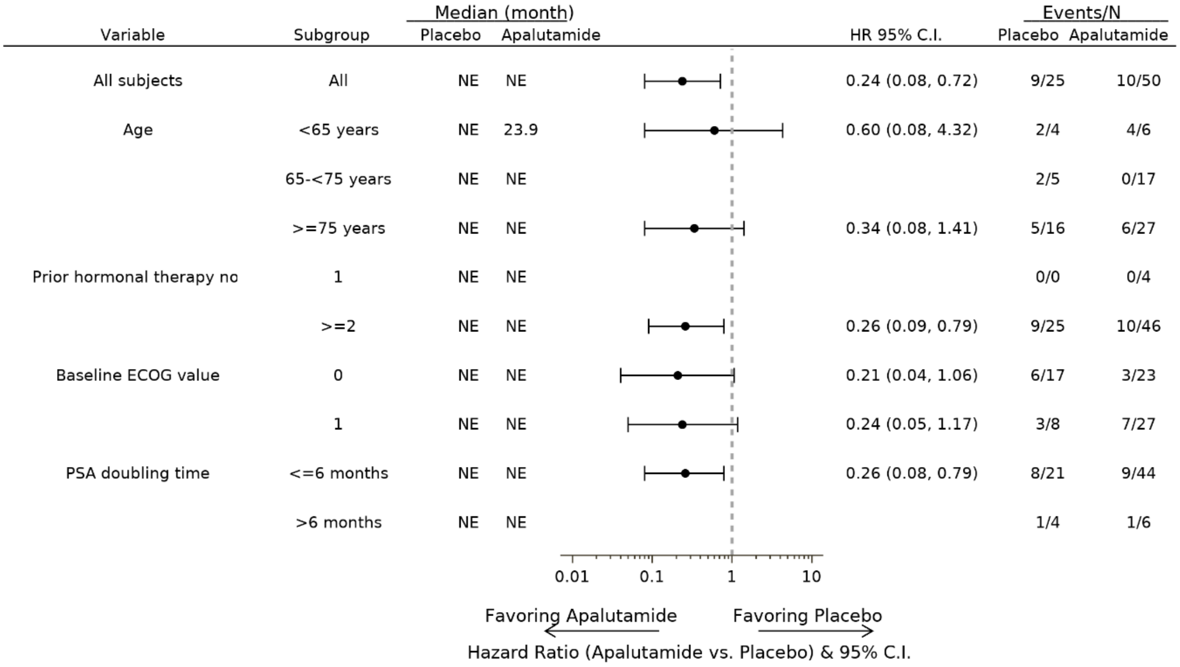

ECOG, Eastern Cooperative Oncology Group; ITT, intent-to-treat; PSA, prostate-specific antigen.

Data are represented as hazard ratio value with 95% confident interval. Hazard ratio results are not reported in subgroups with limited number of events.

Figure S2. Kaplan-Meier plot of metastasis-free survival for the apalutamide arm (ITT population)

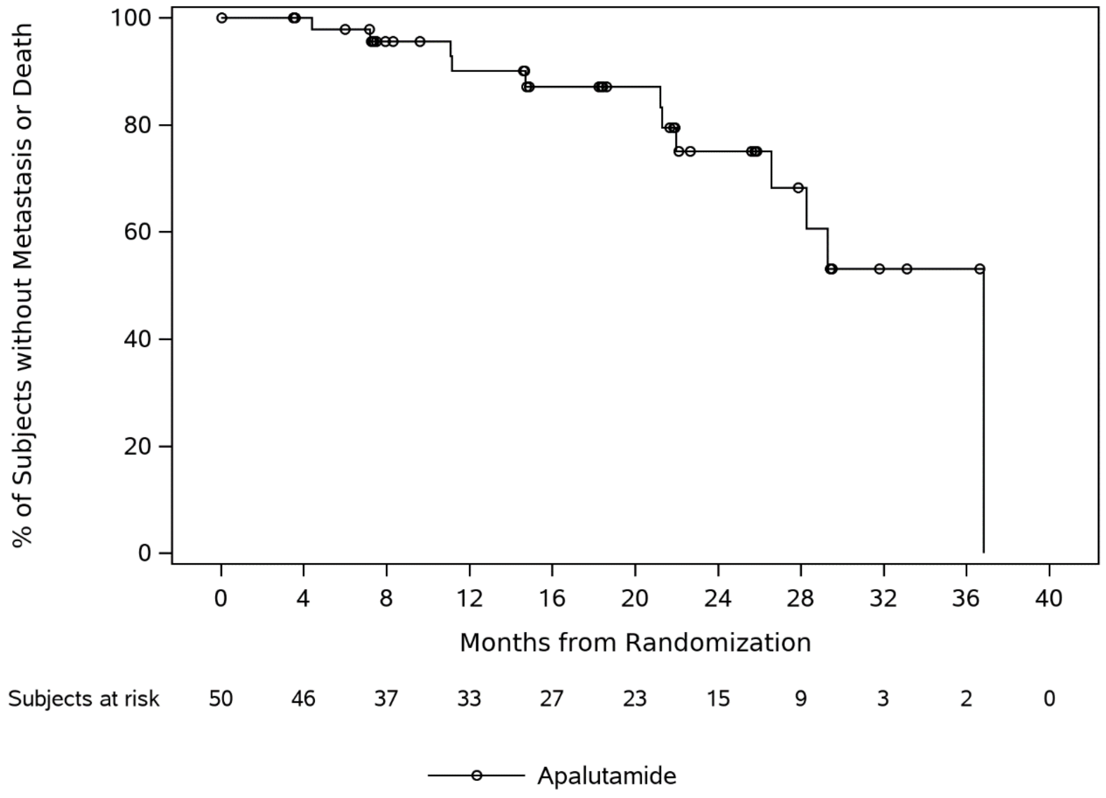

ITT, intent-to-treat.

Figure S3. Kaplan-Meier plot of overall survival in the apalutamide arm (ITT population)

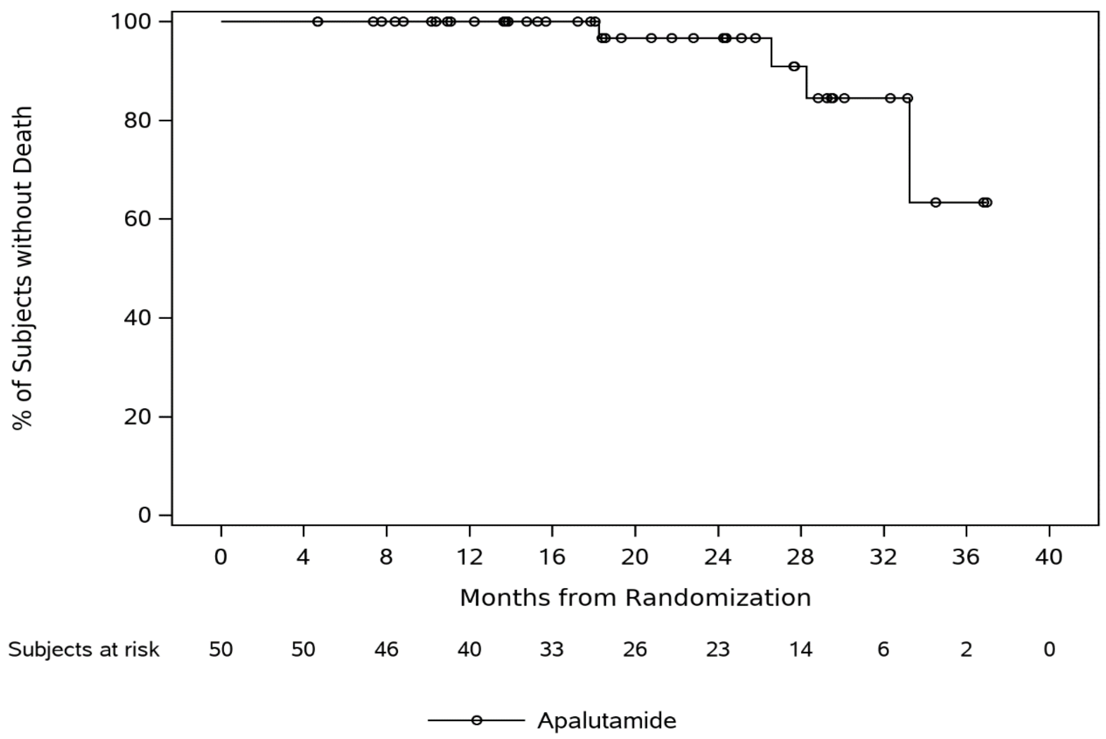

ITT, intent-to-treat.

Methods S1: Study eligibility criteria

Inclusion criteria:

1. Male (according to their reproductive organs and functions assigned by chromosomal complement).
2. ≥18 years of age (or the legal age of consent in the jurisdiction in which the study was taking place).
3. Histologically or cytologically confirmed adenocarcinoma of the prostate without neuroendocrine differentiation or small cell features, with high risk for development of metastases, defined as prostate-specific antigen doubling time (PSADT) ≤10 months. PSADT was calculated using at least 3 values of prostate-specific antigen (PSA) obtained during continuous androgen deprivation therapy (ADT).
4. Castration-resistant prostate cancer (PC) demonstrated during continuous ADT, defined as 3 PSA rises at least 1 week apart, with the last PSA >2 ng/mL.
5. Surgically or medically castrated, with testosterone levels of <50 ng/dL. If the participant was medically castrated, continuous dosing with gonadotropin-releasing hormone agonist (GnRHa) had to initiated at least 4 weeks prior to randomization and had to continue throughout the study to maintain castrate levels of testosterone.
6. Patients who had received a first-generation anti-androgen (eg, bicalutamide, flutamide, nilutamide) had to have at least a 4-week washout prior to randomization AND had continuing disease progression (an increase in PSA) after washout.
7. At least 4 weeks had elapsed from the use of 5-α reductase inhibitors (eg, dutasteride, finasteride), estrogens (irrespective of dose used), and any other anticancer therapy prior to randomization, including chemotherapy given in the adjuvant/neoadjuvant setting (eg, clinical trial).

8. At least 4 weeks had elapsed from major surgery or radiation therapy prior to randomization.
9. Eastern Cooperative Oncology Group (ECOG) - Performance Status (PS) grade 0 or 1.
10. Resolution of all acute toxic effects of prior therapy or surgical procedure to Grade 1 or baseline prior to randomization.
11. Adequate organ function as defined by the following criteria:
    - Serum aspartate aminotransferase (AST; serum glutamic oxaloacetic transaminase [SGOT]) and serum alanine aminotransferase (ALT; serum glutamic pyruvic transaminase [SGPT]) ≤2.5x upper limit of normal (ULN).
    - Total bilirubin ≤1.5 x ULN; participants with Gilbert syndrome can be enrolled if conjugated bilirubin is within normal limits (≤1.5 x ULN or direct bilirubin ≤ULN for participants with total bilirubin levels >1.5 x ULN).
    - Serum creatinine ≤2 x ULN.
    - Absolute neutrophil count (ANC) ≥1500/µL.
    - Platelets ≥100,000/µL.
    - Hemoglobin ≥9.0 g/dL.
    - Administration of growth factors or blood transfusions was not allowed within 4 weeks of the hematology labs required to confirm eligibility.
12. Singed an informed consent form (or their legally acceptable representative must sign) indicating that he understands the purpose of, and procedures required for, the study and is willing to participate in the study.
13. Willingness and ability to comply with scheduled visits, treatment plans, laboratory and radiographic assessments, and other study procedures, including ability to swallow study drug tablets, and long-term survival follow-up visits.
14. Willing and able to adhere to the lifestyle restrictions specified in this protocol.

Exclusion criteria:

1. Presence of distant metastases, including central nervous system (CNS) and vertebral or meningeal involvement, or history of distant metastases. Exception: Pelvic lymph nodes <2 cm in short axis (N1) located below the iliac bifurcation were allowed.
2. Symptomatic loco-regional disease requiring medical intervention, such as moderate or severe urinary obstruction or hydronephrosis, due to primary tumour (eg, tumour obstruction of bladder trigone).
3. Prior treatment with second generation anti-androgens (eg, enzalutamide).
4. Prior treatment with CYP17 inhibitors (eg, abiraterone acetate, orteronel, galerterone, ketoconazole, aminoglutethimide) for PC.
5. Prior treatment with radiopharmaceutical agents (eg, Strontium-89), immunotherapy (eg, sipuleucel-T), or any other investigational agent for NM-CRPC.
6. Prior chemotherapy for PC, except if administered in the adjuvant/neoadjuvant setting.
7. History of seizure or condition that may pre-dispose to seizure (eg, prior stroke within 1 year prior to randomization, brain arteriovenous malformation, Schwannoma, meningioma, or other benign CNS or meningeal disease which might require treatment with surgery or radiation therapy).
8. Concurrent therapy with any of the following (all had to be discontinued or substituted for at least 4 weeks prior to randomization):
    - Medications known to lower the seizure threshold.
    - Herbal and non-herbal products that may decrease PSA levels.
    - Spironolactone.
    - Systemic (oral/IV/IM) corticosteroids. Short term use (≤4 weeks) of corticosteroids during the study is allowed if clinically indicated, but it will be tapered off as soon as possible.
    - Any other experimental treatment on another clinical trial.

- Agents indicated for the prevention of SREs in participants with solid tumours (eg, denosumab [XGEVA®]), zoledronic acid [ZOMETA®]). Treatment with bone-sparing agents for the treatment of osteoporosis (eg, denosumab [PROLIA®], zoledronic acid [ACLASTA®]) is not exclusionary.

9. History of severe/unstable angina, myocardial infarction, symptomatic congestive heart failure, arterial or venous thromboembolic events (eg, pulmonary embolism, cerebrovascular accident including transient ischemic attacks) or clinically significant ventricular arrhythmias within 6 months prior to randomization.
10. History of malignancy within 5 years before screening (exceptions were squamous and basal cell carcinomas of the skin, superficial bladder cancer.
11. Uncontrolled hypertension (systolic blood pressure (BP) ≥160 mm Hg or diastolic BP ≥100 mm Hg). Patients with a history of uncontrolled hypertension were allowed provided BP is controlled by anti-hypertensive treatment.
12. Gastrointestinal disorder affecting absorption.
13. Active infection, such as human immunodeficiency virus, or active hepatitis.
14. Had received an investigational intervention (including investigational vaccines) or used an invasive investigational medical device within 4 weeks before the planned first dose of study intervention or was being enrolled in an investigational study.
15. Men who were sexually active with women of childbearing potential except:
    - agreed to use a condom with spermicidal foam/gel/film/cream/suppository.
    - agreed to not donate sperm during the study and for at least 3 months after the last dose of study drug.
    - did not plan to father a child during the study or within 3 months after the last dose of study drug.

16. Any condition for which, in the opinion of the investigator, participation would not be in the best interest of the participant (eg, compromise the well-being) or that could prevent, limit, or confound the protocol-specified assessments.
17. Employee of the investigator or study site, with direct involvement in the proposed study or other studies under the direction of that investigator or study site, as well as family members of the employees or the investigator.
18. Known allergies, hypersensitivity, or intolerance to apalutamide or its excipients.

Table S1. List of Independent Ethics Committee/Institutional Review Board that approved this study

Study site	Name of Independent Ethics Committee/Institutional Review Board	Ethics Committee approval number
Zhejiang Provincial People's Hospital	Ethics Committee of Zhejiang Provincial People's Hospital	2019YW048
Nanjing Drum Tower Hospital	Ethics Committee of Nanjing Drum Tower Hospital	2019-226-03
Shanghai Zhongshan Hospital	Ethics Committee of Shanghai Zhongshan Hospital	2019-113R
Hunan Cancer Hospital	Ethics Committee of Hunan Cancer Hospital	JXHL1800179
Liaoning Cancer Hospital & Institute	Ethics Committee of Liaoning Cancer Hospital & Institute	20191127-1
Huashan Hospital Fudan University	Ethics Committee of Huashan Hospital Fudan University	(2020)临审第（033）号
Peking University First Hospital	Ethics Committee of Peking University First Hospital	2019-0115
Sun Yat-Sen Memorial Hospital Sun Yat-sen University	Ethics Committee of Sun Yat-Sen Memorial Hospital Sun Yat-sen University	2019-YW-082
Chongqing University Cancer Hospital	Ethics Committee of Chongqing University Cancer Hospital	2019(179)
Peking University Third Hospital	Ethics Committee of Peking University Third Hospital	2019 药伦审第（121-03）号
First Affiliated Hospital, SooChow University	Ethics Committee of First Affiliated Hospital, SooChow University	（2019）伦审批第 148-3 号
Sichuan Provincial People's Hospital	Ethics Committee of Sichuan Provincial People's Hospital	伦审(药)2020 年第 7 号
The Fifth People's Hospital of Shanghai, Fudan University	Ethics Committee of The Fifth People's Hospital of Shanghai, Fudan University	(2019)伦审（141）号
Huadong Hospital Affiliated to Fudan University	Ethics Committee of Huadong Hospital Affiliated to Fudan University	20190118
Beijing Friendship Hospital	Ethics Committee of Beijing Friendship Hospital	2019-PI-药 046-01
TongJi Hospital of TongJi Medical College of Huazhong University of Science & Technology	Ethics Committee of TongJi Hospital of TongJi Medical College of Huazhong University of Science & Technology	[2019]伦审字（317）-2 号
Guangzhou First Municipal People's Hospital	Ethics Committee of Guangzhou First Municipal People's Hospital	A-2019-024-02
Cancer Hospital Chinese Academy of Medical Sciences	Ethics Committee of Cancer Hospital Chinese Academy of Medical Sciences	19/193-1977

Wuxi People's Hospital	Ethics Committee of Wuxi People's Hospital	2019LLPJ-IV-38
Renji Hospital, Shanghai Jiaotong University School of Medicine	Ethics Committee of Renji Hospital, Shanghai Jiaotong University School of Medicine	2019-035
Cancer Hospital, FuDan University	Ethics Committee of Cancer Hospital, FuDan University	1908205-11
Zhejiang Cancer Hospital	Ethics Committee of Zhejiang Cancer Hospital	IRB-[2019]721
Jiangsu Cancer Hospital	Ethics Committee of Jiangsu Cancer Hospital	2020-047
Peking University People's Hospital	Ethics Committee of Peking University People's Hospital	2019PHA092-001
Yunnan Cancer Hospital	Ethics Committee of Yunnan Cancer Hospital	YW202028
The First Affiliated Hospital, Zhejiang University	Ethics Committee of The First Affiliated Hospital, Zhejiang University	PRO20200028
Beijing Hospital	Ethics Committee of Beijing Hospital	2019BJYYEC-209-01
Fujian Medical University Union Hospital	Ethics Committee of Fujian Medical University Union Hospital	2020YW020-07
First Affiliated Hospital, Xi'an Jiaotong University	Ethics Committee of First Affiliated Hospital, Xi'an Jiaotong University	XJTU1AF2019LSY-126
Ningbo First Hospital	Ethics Committee of Ningbo First Hospital	2019-D010-YJ01
