# Supplementary material for: A randomized study of apalutamide in Chinese patients with non-metastatic castration-resistant prostate cancer
Source: iScience. 2025 Jul 18;28(8):113166. doi: 10.1016/j.isci.2025.113166 (PMC12355417; doi:10.1016/j.isci.2025.113166)
Supplement: Data S1. CONSORT checklist, study protocol, and study statistical analysis plan [file mmc2.zip › Statistical Analysis Plan.pdf]

Janssen Research & Development

Statistical Analysis Plan

A Multicenter, Randomized, Double-Blind, Placebo-Controlled, Phase IV study of Apalutamide in Chinese Participants with Non-Metastatic Castration-Resistant Prostate Cancer (NM-CRPC)

Protocol 56021927PCR4007; Phase 4
Amendment 1

JNJ-56021927 (apalutamide)

**Status:**	Approved
**Date:**	12 July 2023
**Prepared by:**	Janssen Research & Development, LLC
**Document No.:**	EDMS-RIM-1088345, 1.0

Compliance: The study described in this report was performed according to the principles of Good Clinical Practice (GCP).

Confidentiality Statement
The information in this document contains trade secrets and commercial information that are privileged or confidential and may not be disclosed unless such disclosure is required by applicable law or regulations. In any event, persons to whom the information is disclosed must be informed that the information is privileged or confidential and may not be further disclosed by them. These restrictions on disclosure will apply equally to all future information supplied to you that is indicated as privileged or confidential.

TABLE OF CONTENTS

TABLE OF CONTENTS **2**

AMENDMENT HISTORY **4**

ABBREVIATIONS **6**

1. INTRODUCTION **7**
1.1. Trial Objectives 7
1.2. Trial Design 7
1.3. Statistical Hypotheses for Trial Objectives 8
1.4. Sample Size Justification 9
1.5. Randomization and Blinding 9
1.5.1. Randomization 9
1.5.2. Blinding 10

2. GENERAL ANALYSIS DEFINITIONS **10**
2.1. Visit Windows 11
2.2. Pooling Algorithm for Analysis Centers and Stratification Factors 11
2.3. Analysis Sets 11
2.3.1. Efficacy Analysis Set 11
2.3.2. Safety Analysis Set 11
2.3.3. Pharmacokinetics Analysis Set 11
2.4. Definition of Subgroups 11
2.5. Study Day and Relative Day 11
2.6. Baseline 12
2.7. Imputation Rules for Missing AE Date/Time of Onset/Resolution 12

3. INTERIM ANALYSIS AND DATA MONITORING COMMITTEE REVIEW **13**
3.1. Independent Data Monitoring Committee (IDMC) 13
3.2. Interim Analysis for Efficacy 13

4. SUBJECT INFORMATION **13**
4.1. Demographics and Baseline Characteristics 13
4.2. Disposition Information 14
4.3. Treatment Compliance 14
4.4. Extent of Exposure 14
4.5. Protocol Deviations 14
4.6. Prior and Concomitant Medications 15

5. EFFICACY **15**
5.1. Analysis Specifications 15
5.1.1. Level of Significance 15
5.2. Primary Efficacy Endpoint 15
5.2.1. Definition 16
5.2.2. Estimand 16
5.2.3. Analysis Methods 17
5.3. Secondary Endpoint 17
5.3.1. Definition 17
5.3.2. Analysis Methods 18
5.4. Exploratory Efficacy Variables 18
5.4.1. Definition 18
5.4.2. Analysis Methods 18

6. SAFETY **19**
6.1. Adverse Events 19

6.2. Clinical Laboratory Tests 20
6.3. Vital Signs and Physical Examination Findings 21
6.4. Electrocardiogram 21

7. PHARMACOKINETICS/PHARMACODYNAMICS 21

REFERENCES 22

AMENDMENT HISTORY

SAP Version	Issue Date
Amendment 1	12Jul2023
Original SAP	30Apr2020

Amendments below are listed beginning with the most recent amendment.

Amendment 1 (12Jul2023)

Applicable Section(s)	Description of Change(s)	Rationale
Section 1.2. Trial Design; Section 1.4. Sample Size Justification; Section 1.5.2. Blinding; Section 3. INTERIM ANALYSIS AND DATA MONITORING COMMITTEE REVIEW	• Changed the number of participants to be enrolled from 111 to 75.<br>• Removed the interim analysis.<br>• Revised the role of Independent Data Monitoring Committee (IDMC).<br>• Revised the unblinding circumstance.	The change was made based on CDE consultation outcome and to align with protocol amendment 3. Since interim analysis is not applicable anymore and study will be unblinded at the final analysis, the role and responsibility of IDMC was also revised.
Section 2.2. Pooling Algorithm	• Added the pooling algorithm for stratification factors.	Stratified analysis may lead to reduced statistical power if the sample size within each stratum is small.
Section 4.2. Disposition Information; Section 4.5. Protocol Deviations; Section 5.2.3 Analysis Method; Section 6.1. Adverse Events	• Added analyses for COVID-19 related data in applicable sections.	To explore the potential impact of the COVID-19 pandemic on the study outcomes, additional analyses were added.
Section 5.2.2. Estimand	• Intercurrent events of clinical cutoff and treatment discontinuation were deleted.<br>• The strategy name for treatment switching and subsequent anti-cancer was revised.	Clinical cutoff is a pre-determined timepoint; treatment discontinuation in this study will lead to an administrative stop of subsequent PSA testing. Neither are considered intercurrent events.<br><br>The strategy name was adjusted according to the definition of each strategy in ICH E9(R1).

Section 5.2.3. Analysis Methods	• Sensitivity analysis regarding additional censoring rules for TTPP event dates was deleted.<br><br>• Revised the time point to estimate the PSA progression-free rate from "6-month, 1-year and 2-year" to "at selected timepoints".	The additional censoring rule for TTPP event dates explores an alternative clinical question of interest (Estimand), which differs from the primary objective in its focus and assumption.<br><br>Considering the censoring rule of placebo arm, the estimating timepoints were revised.
Section 5.3.2. Analysis Methods	• Added Fisher's exact test.<br><br>• Added PSA response by Week 12.	Fisher's Exact Test may provide a more accurate assessment of associations in the contingency table when some cells have low counts.<br><br>PSA response by Week 12 can provide valuable insights and comprehensive understanding into the treatment effect.
Section 6.1. Adverse Events	• Narrative criteria were revised.	The narrative criteria were revised to keep consistent with SPARTAN study.
Throughout the SAP	• Minor grammatical, formatting, or spelling changes were made.	Minor errors were noted.

ABBREVIATIONS

ADT	androgen deprivation therapy
AE	adverse event
CI	confidence interval
CRF	case report form
CRPC	castration-resistant prostate cancer
CSR	Clinical Study Report
CTCAE	Common Terminology Criteria for Adverse Events
ECG	Electrocardiogram
ECOG	Eastern Cooperative Oncology Group
eCRF	electronic case report form
EoT	end of treatment
GnRH	gonadotropin releasing hormone
HR	hazard ratio
IA	Interim Analysis
IDMC	Independent Data Monitoring Committee
ITT	Intent-to-Treat
IWRS	interactive web response system
K-M	Kaplan-Meier
NM-CRPC	non-metastatic castration-resistant prostate cancer
MedDRA	Medical Dictionary for Regulatory Activities
MFS	metastasis-free survival
MRI	magnetic resonance imaging
OS	overall survival
PCWG2	Prostate Cancer Working Group 2
PK	pharmacokinetic(s)
PSA	prostate specific antigen
PSADT	prostate specific antigen (PSA) doubling time
SAE	serious adverse event
SAP	Statistical Analysis Plan
SD	standard deviation
SOC	system organ class
SRE	skeletal related events
TTPP	time to prostate specific antigen (PSA) progression
WHO	World Health Organization

1. INTRODUCTION

This statistical analysis plan (SAP) describes the definitions of analysis sets, derived variables, and statistical methods for all planned statistical analyses in protocol 56021927PCR4007.

1.1. Trial Objectives

Primary Objectives:

To compare the improvement in time to prostate specific antigen (PSA) progression (TTPP, as defined by Prostate Cancer Working Group 2 [PCWG2]) of apalutamide versus placebo in Chinese subjects with high risk NM-CRPC.[1]

Secondary Objectives:

- To evaluate the safety and tolerability of apalutamide
- To compare the prostate-specific antigen (PSA) response rate in Chinese subjects with high risk NM-CRPC treated with apalutamide versus placebo
- To evaluate the pharmacokinetics (PK) of apalutamide

Exploratory Objectives:

- To evaluate the metastasis-free survival (MFS) of Chinese subjects with high risk NM-CRPC treated with apalutamide
- To evaluate the overall survival (OS) of Chinese subjects with high risk NM-CRPC treated with apalutamide
- To evaluate the time to symptomatic progression in Chinese subjects with high risk NM-CRPC treated with apalutamide

1.2. Trial Design

This is a randomized, double-blind, placebo-controlled, multicenter, Phase 4 study evaluating the efficacy and safety of ADT plus apalutamide versus ADT plus placebo in Chinese subjects with high-risk NM-CRPC.

The study will consist of 4 phases: an optional Prescreening Phase, a Screening Phase (≤35 days prior to randomization); a Treatment Phase (starting on day 1 of cycle 1 until disease progression/ unacceptable toxicity, withdrawal of consent, death or termination of the study) and a post-treatment Follow-up Phase (starting after end-of-treatment visit until death, withdrawal of consent for study participation, or termination of the study).

Approximately 75 Chinese subjects who meet all the inclusion criteria and none of the exclusion criteria will be randomized in a 2:1 ratio to receive apalutamide or matching placebo. Subjects will be stratified by PSADT (>6 months vs. ≤6 months). Apalutamide or matched placebo will be administered orally on a continuous daily dosing schedule at a starting dose of 240 mg per day in the Treatment Phase. ADT with gonadotrophin-releasing hormone agonists (GnRHa) will be continued for all patients who have not been surgically castrated. Subjects randomized to the

placebo arm who do not have evidence of distant metastasis (according to imaging result at Cycle 5 Day 1, as well as at any other relevant unscheduled visit) will be crossed over and start receiving active therapy of apalutamide after completion of 5 cycles of placebo + ADT treatment (handling by the IWRS). Placebo subjects who have documented PSA progression prior to completion of 5 cycles of study treatment, will cross over to apalutamide at the time of PSA progression. Any subjects who develop distant metastasis at any time on study will discontinue study treatment and may receive subsequent therapy as per standard of care.

Subjects will be followed for safety and efficacy as per the schedule of activities and will remain on study treatment until development of distant metastases as assessed by investigators or the development of unacceptable toxicity or the study end defined as MFS events observed in approximately 60% of the study population or 3 years after the final analysis for the primary endpoint TTPP, whichever comes first. An End of Treatment (EoT) Visit will occur approximately 30 days of the last dose of study treatment. The Post-treatment Follow-up Phase will begin after the EoT visit. Subjects will be contacted every 4 months to obtain survival status, recording of development of symptomatic progression, initiation of any new systemic anti-cancer therapies until death, withdrawal of consent for study participation, or termination of the study. The final analysis for the primary endpoint TTPP will occur at 5 months after the last subject received the first dose.

Figure 1

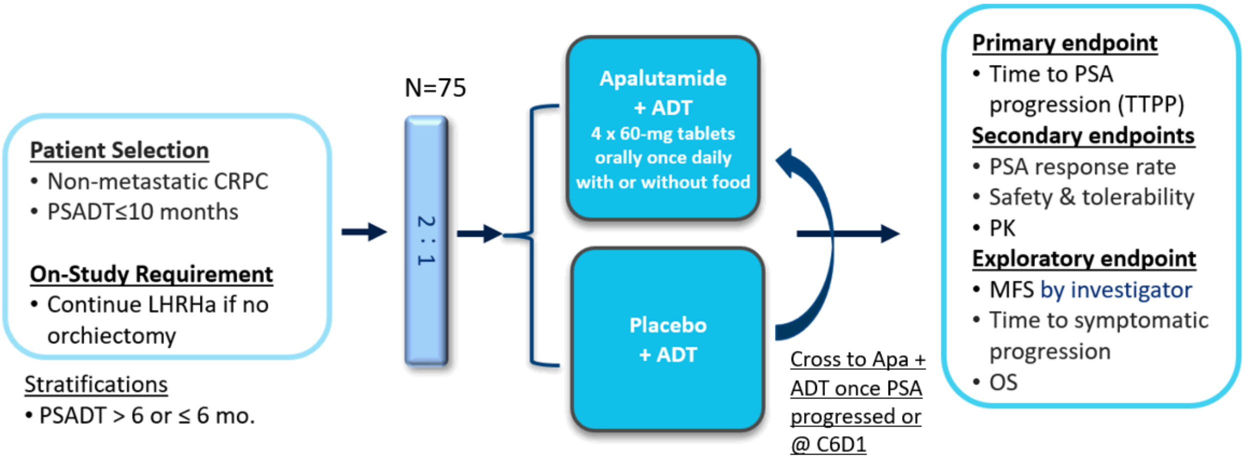

Note: ADT: androgen deprivation therapy, being either surgical castration or gonadotrophin-releasing hormone agonists (GnRHa), will be continued as background therapy for all subjects. ADT with GnRHa will be continued for all patients who have not been surgically castrated.

1.3. Statistical Hypotheses for Trial Objectives

The primary hypothesis of this study is that subjects receiving treatment with apalutamide will have a longer TTPP compared with subjects receiving placebo. The null hypothesis of this study is no difference between these two treatment groups in TTPP.

1.4. Sample Size Justification

Subjects randomized to the placebo arm who do not have evidence of distant metastasis (according to imaging result at Cycle 5 Day 1, as well as any other relevant unscheduled visit) will start receiving active therapy of apalutamide at the time they have documented PSA progression (per PCWG2) or after completion of 5 cycles of placebo+ADT treatment (handling by the IWRS). Any subjects who develop distant metastasis at any time on study will discontinue study treatment, and may receive subsequent therapy as per standard of care.

It is assumed that failure distribution of the primary endpoint TTPP follows an exponential distribution with a constant hazard rate. The pivotal SPARTAN study suggested that East Asian subjects treated with ADT would only have a median TTPP of 3.7 months. It is estimated that a sample size of 75 subjects with follow-up continued up to approximately 5 months after last subject dosed will provide approximately 28 TTPP events to yield a power exceeding 95% for detecting a hazard ratio (HR) of 0.11 (median TTPP of 3.7 months for the placebo arm versus 33.6 months for the apalutamide arm, here 0.11 is the upper limit of 95% CI in the TTPP results of East Asian) at a 2-tailed significance level of 0.05. With a 32-month accrual period and an additional 5-months of follow-up, the study duration for the TTPP final analysis will be approximately 37 months.

1.5. Randomization and Blinding

1.5.1. Randomization

Subjects who meet all the inclusion criteria and none of the exclusion criteria will be randomized in a 2:1 ratio to receive apalutamide or placebo. Subjects will be stratified by the following factor for randomization:

- PSADT: >6 months vs. ≤6 months

Randomization will take place across all study sites using a centralized Interactive Web Response System (IWRS).

To ensure an accurate and consistent determination of PSADT across all sites, the IWRS will provide PSADT calculations (using a linear regression model of the normal logarithm of PSA and time) based on at least 3 PSA values obtained during continuous ADT. All available consecutive PSA values obtained within 24 months prior to randomization beginning with the earliest value chosen for the PSADT calculation must be entered in the IWRS. The first and last PSA values used in the calculation must be separated by at least 8 weeks.

Those same PSA values will be used during Screening to determine whether the patient is eligible for the study (inclusion criterion #3). To pre-screen subjects for possible enrollment into the study, PSADT can be calculated using the Memorial Sloan-Kettering Cancer Center (MSKCC) PSADT prediction tool, available at the following website:

http://nomograms.mskcc.org/Prostate/PsaDoublingTime.aspx

1.5.2. Blinding

All subjects and study team members associated with the study conduct are to remain blinded to treatment group assignment until final analysis. Unblinding before final analysis may only occur in case of safety or medical emergency .

PSA will be collected and analyzed by a central laboratory but investigators, participants, and the Sponsor will be blinded to the results until the full unblinding of the study. The cross-over of control group subjects will be handled by IWRS to maintain the blinding of original individual treatment assignment. Control group subjects without distant metastasis will switch to treatment with apalutamide plus ADT at the time PSA progression (per PCWG2) is documented or subjects have completed 5 cycles treatment of placebo + ADT. The PSA data collected at baseline and post-baseline obtained within the first 5 cycles, for individual subject will be analyzed by the central lab to flag out documented PSA progression per PCWG2. The data will be integrated into the IWRS to trigger the cross-over.

2. GENERAL ANALYSIS DEFINITIONS

Study Day is calculated in reference to the date of randomization for efficacy analyses and date of first dose for safety analyses (efficacy analysis Study Day 1 = date of randomization; safety analysis Study Day 1 = date of first dose). Positive study days will count forward from Study Day 1. Study Day -1 will be the day before Study Day 1, and all subsequent negative study days will be measured backward from Study Day -1. There will be no Study Day 0.

The first dose starts on Day 1 of Cycle 1.

Unless otherwise specified, baseline value is defined as the closest measurement prior to the first dose of study drug. Change from baseline will be defined as (post-baseline value – baseline value).

Treatment Duration is calculated as the duration of time from the date of the first dose of study drug to the date of last dose of the study drug, ie, date of last dose – date of first dose + 1.

Time to Event is calculated as the number of days from randomization to the date of the event of interest, ie, date of event of interest – date of randomization + 1. Time to event endpoints will be based on the actual date of the event, not visit number or visit label.

Continuous/numerical variables will be summarized using mean, standard deviation (SD), median, minimum, and maximum. Categorical variables will be summarized using count and percentage. In general, means and other measures of central tendency will be displayed with one more decimal digit than collected, and standard deviations and other measures of variability will be displayed with two decimal digits more than collected. For frequency counts of categorical variables, categories for which the counts are zero will be displayed for the sake of completeness. For example, if no subject discontinued due to "withdrawal of consent", this reason would still be included in the table but with a count of 0.

2.1. Visit Windows

The Treatment Phase will begin at Cycle 1 Day 1 of treatment and will continue until study drug is discontinued. Nominal visits will be used to display the safety data.

2.2. Pooling Algorithm for Analysis Centers and Stratification Factors

There is no plan in pooling the centers (study sites) for efficacy or safety analyses, unless analysis by site is warranted. The stratification factor to be used in the analysis is PSADT. For efficacy analyses, if any stratum has few events (for time-to-event endpoint) or participants (for categorical endpoint) for any treatment arm, then the subjects will be pooled together by dropping the stratification factor.

2.3. Analysis Sets

2.3.1. Efficacy Analysis Set

The Intent-to-Treat (ITT) analysis set includes all randomized subjects and will be classified according to their assigned treatment group, regardless of the actual treatment received. Subject disposition, demographics and baseline characteristics, as well as efficacy analyses will be performed on data from the ITT analysis set.

2.3.2. Safety Analysis Set

The safety analysis set includes all randomized subjects who received at least 1 dose of study drug, with treatment assignments designated according to actual study treatment received. The safety analysis set will be used in the analyses of safety and treatment compliance.

2.3.3. Pharmacokinetics Analysis Set

The PK analysis set is defined as all subjects who have received at least one dose of apalutamide and have at least one quantifiable plasma concentration.

2.4. Definition of Subgroups

In order to assess the consistency of treatment benefit across different subject subpopulations, the planned analyses may also be carried out using the following subgroups in TTPP (see Section 5):

- ECOG performance status (0 vs. 1) at baseline
- Age category (< 65 vs. ≥ 65 years and < 75 vs. ≥ 75 years)
- Number of prior hormonal therapies (1 vs. ≥ 2)
- PSADT (> 6 months vs. ≤ 6 months)

2.5. Study Day and Relative Day

Study Day 1 or Day 1 refers to the start of the first study drug administration for safety analysis or randomization date for efficacy analysis. All efficacy and safety assessments at all visits will be assigned a day relative to this date.

Study day or relative day for a visit is defined as:

- Visit date - date of Day 1 +1, if visit date ≥date of Day 1
- Visit date - date of Day 1, if visit date <date of Day 1

There is no 'Day 0'

Study Day is calculated in reference to the date of randomization for efficacy analyses and date of first dose for safety analyses (efficacy analysis Study Day 1 = date of randomization; safety analysis Study Day 1 = date of first dose). Positive study days will count forward from Study Day 1. Study Day -1 will be the day before Study Day 1, and all subsequent negative study days will be measured backward from Study Day -1.

2.6. Baseline

Unless otherwise specified, baseline is defined as the last observation prior to the start of the first study drug administration. Change from baseline will be defined as (post-baseline value – baseline value).

2.7. Imputation Rules for Missing AE Date/Time of Onset/Resolution

Partial AE onset dates will be imputed as follows:

- If the onset date of an adverse event is missing day only, it will be set to:
  - First day of the month that the AE occurred, if month/year of the onset of AE is different from the month/year of the study drug start
  - The day of study drug start, if the month/year of the onset of AE is the same as month/year of the study drug start date and month/year of the AE resolution date is different
  - The day of study drug start or day of AE resolution date, whichever is earliest, if month/year of the onset of AE and month/year of the study drug start date and month/year of the AE resolution date are same
- If the onset date of an adverse event is missing both day and month, it will be set to the earliest of:
  - January 1 of the year of onset, as long as this date is on or after the study drug start date
  - Month and day of the study drug start date, if this date is the same year that the AE occurred
  - Last day of the year if the year of the AE onset is prior to the year of the study drug start date,
  - The AE resolution date.
- Completely missing onset dates will not be imputed.

Partial AE resolution dates not marked as ongoing will be imputed as follows:

- If the resolution date of an adverse event is missing day only, it will be set to the earliest of the last day of the month of occurrence of resolution or the day of the date of death, if the death occurred in that month.

- If the resolution date of an adverse event is missing both day and month, it will be set to the earliest of December 31 of the year or the day and month of the date of death, if the death occurred in that year.
- Completely missing resolution dates will not be imputed.

3. INTERIM ANALYSIS AND DATA MONITORING COMMITTEE REVIEW

3.1. Independent Data Monitoring Committee (IDMC)

An IDMC was commissioned to review the efficacy and safety data at the originally planned interim analysis prior to Protocol Amendment 3 (PA3). Per PA3, interim analysis is no longer applicable and the study will be unblinded at the final analysis when 75 subjects completed 5 cycles of study treatment. However, the IDMC will continue to be engaged to ensure that they are informed of any potential safety issue or emerging data. The IDMC will help review final analysis results and may provide recommendations to the sponsor regarding study conclusions or further analyses. Complete details regarding the IDMC responsibilities, authorities, and procedures will be documented in the IDMC charter.

3.2. Interim Analysis for Efficacy

No interim analysis is planned for this study.

4. SUBJECT INFORMATION

4.1. Demographics and Baseline Characteristics

The following parameters will be summarized by treatment group using the ITT analysis set:

- Age, race, ethnicity, weight, height
- ECOG performance status
- Baseline PSA value and PSA Doubling Time (PSADT)
- Time (months) from initial diagnosis of prostate cancer to randomization
- Total Gleason Score at initial diagnosis
- Tumor stage at initial diagnosis
- Lymph nodes stage at initial diagnosis
- Number of prior hormonal therapies
- History of surgical prostate cancer procedures (Yes/No)
- Type of surgical procedures (prostatectomy, orchiectomy, transurethral resection of the prostate (TURP), and other)
- History of radiotherapy (Yes/No)
- Type of radiotherapy
- History of prior chemotherapy (Yes/No)

4.2. Disposition Information

The number and percentage of subjects who are screened, screen failed, enrolled (randomized), dosed, or have study or treatment early terminated as well as the reason for discontinuation (including COVID-19 related) will be summarized by treatment group using ITT analysis set.

4.3. Treatment Compliance

Treatment compliance and dose modifications will be summarized by treatment group using the safety analysis set.

Treatment duration will be defined as the duration of time from the date of the first dose of study drug to the date of last dose of study drug + 1 day.

Drug compliance will be summarized by the treatment group and the percent treatment compliance will be defined as the number of tablets taken during the study divided by the expected number of tablets, multiplied by 100. The number of tablets taken will be calculated based on the number of tablets dispensed minus the number of returned tablets. A subject’s expected number of tablets will be calculated according to the planned treatment regimen. For example, for subjects with no dose modification the expected number of tablets equals the number of assigned tablets per day multiplied by treatment duration, however for subjects with dose reduction the expected number of tablets will be smaller. Subjects with at least one dose reduction or with dose interruption and the reason for the dose modification will be summarized by treatment group.

4.4. Extent of Exposure

Extent of exposure will be summarized by treatment group using the safety analysis set, in terms of treatment duration in cycles and in months, which are calculated as the number of days with dosing record divided by 30.4375 (ie, number of days in a month calculated as 365.25/12).

4.5. Protocol Deviations

Protocol deviations will be summarized by treatment group using the ITT. Protocol deviations will be reviewed on a case-by-case basis and major protocol deviations (including COVID-19 related) will be identified and summarized by the following example categories:

- Deviation from inclusion/exclusion criteria
- Received wrong treatment or incorrect dose
- Received disallowed concomitant treatments
- Other

The categories will be finalized prior to database lock.

If warranted, the protocol deviations that are specifically related to inclusion/exclusion criteria will also be summarized.

COVID-19 related protocol deviations will be listed.

4.6. Prior and Concomitant Medications

Prior and concomitant medications will be summarized by treatment group, World Health Organization (WHO) Drug therapeutic class, and generic medication name using the safety analysis set. Prior medications are those taken (with medication start date) prior to Cycle 1 Day 1. Concomitant medications are those, other than medications involved in experiment, taken during the Treatment Phase, ie, with medication start date on or prior to the 30th day after the last dose of study drug and medication stop date on or after Cycle 1 Day 1 or ongoing.

Subsequent prostate cancer therapies received after the Treatment Phase will be summarized by treatment group using the efficacy analysis set. If the therapy is medication, then it will also be summarized by WHO Drug therapeutic class and generic medication name.

5. EFFICACY

The following section outlines the planned analyses of the primary, secondary efficacy, and the exploratory efficacy outcomes of the study.

Efficacy analyses will be performed using the ITT population, incorporating the randomization stratification factor as documented on the IWRS, unless otherwise specified.

Time-to-event endpoints will be summarized using the Kaplan-Meier method and displayed graphically where appropriate.[3] Median event times and 2-sided 95% confidence interval for each median will be provided. Cox proportional-hazard models, including the stratification factor at baseline, will be used to estimate the hazard ratio (HR) and its 95% confidence interval (CI) for TTPP.

Response endpoints (e.g. PSA response rate) will be summarized using descriptive statistics for categorical data by treatment group. The relative risk (treatment:control) will be reported along with the associated 2-tailed 95% CIs. The two treatment groups will be compared using the stratified Mantel-Haenszel test if data warranted; Fisher's exact test may be used if the expected counts in some cells are small.

5.1. Analysis Specifications

All efficacy endpoints will be summarized by treatment group using the ITT. When stratified analyses are performed, strata used by the interactive web response system (IWRS) during subject randomization will be used unless otherwise specified.

5.1.1. Level of Significance

In general, a two-sided significance level of α=0.05 will be used for all hypothesis testing and all confidence intervals will be calculated on the two-sided 95% confidence level (CI), unless otherwise specified.

5.2. Primary Efficacy Endpoint

The primary endpoint is time to prostate specific antigen (PSA) progression (TTPP, as defined by Prostate Cancer Working Group 2 [PCWG2])

5.2.1. Definition

Time to prostate specific antigen progression (TTPP) - defined as the time from randomization to the first date of documented PSA progression based on Prostate Cancer Working Group 2 (PCWG2) criteria.

Apalutamide group subjects will be censored at the last date of PSA assessment if not PSA progressed at the time of analysis. For the control group, subjects who switched treatment and had not yet PSA progressed at that time will be censored at the date treatment switching. For all randomized subjects who received anticancer subsequent therapy before PSA progression documented, the TTPP will also be censored at the last date of PSA assessment prior to the start of subsequent therapy.

5.2.2. Estimand

Population:

Subjects with high-risk (defined as PSA Doubling Time (PSADT) ≤10 months) Non-Metastatic Castration-Resistant Prostate Cancer (NM-CRPC).

Variables:

Time to Event: TTPP

Intercurrent Events and Strategies (Table 1):

Analysis	Variable	Intercurrent Events	Strategies
**Primary**	**TTPP**	• Treatment switching: censoring those subjects who are event-free when treatment switching at the date of occurrence of intercurrent event, regardless if the subjects experienced the event of interest afterward<br>• Death: censoring those subjects that are event-free at the last date where it is known the patients have yet to experience the event of interest to estimate the hypothetical estimand as if we had observed all patients until their endpoint event<br>• Subsequent anti-cancer therapies: censoring those subjects who are event-free at the last date where it is known the patients have yet to experience the event of interest prior to the start of occurrence of intercurrent event	• Hypothetical strategy<br>• Hypothetical strategy<br>• Hypothetical strategy

Population level summary:

Survival endpoint of TTPP: log-rank test and hazard ratio between the two treatment groups with stratification if data warranted.

5.2.3. Analysis Methods

The final analysis of TTPP will be performed at approximately 5 months after last subjects dosed.

The distribution of TTPP will be estimated using the Kaplan-Meier (KM) method. Median TTPP, as well as PSA progression free rate at selected timepoints, and the corresponding confidence interval will be reported with Kaplan-Meier curves. The comparison of TTPP between the 2 treatment groups will be based on log-rank test stratified, if data warranted, by the randomization stratification factor (PSADT [>6 months vs. ≤6 months]). The hazard ratio and corresponding confidence interval will be estimated using a Cox proportional hazards model stratified, if data warranted, by aforementioned randomization stratification factor.

The proportional hazard assumption will be assessed graphically by plotting log(-log[estimated survival distribution function]) against log(survival time). The resulting graphs should have approximately parallel lines when the assumption holds. If the proportional hazard assumption is reasonably met, then the HR will be used as an estimate of treatment effect. If the proportional hazard assumption is considered violated, then the statistical comparison between survival distributions remains valid, but treatment effect may only be evaluated using the median time to event in each treatment group.

Non-stratified log-rank test may be performed as sensitivity analyses to evaluate the robustness of TTPP analysis results if stratified test is warranted by the data.

Finally, to assess the consistency of treatment benefit across subgroups for TTPP, a forest plot of estimated hazard ratios, and associated 95% CIs will be provided for subgroups defined in Section 2.4. To estimate the HR, and CI in each subgroup, the Cox proportional hazard model will be univariate (i.e., including only treatment group).

Missingness of PSA assessment due to COVID-19 will be summarized to evaluate COVID-19 impact on disease assessment.

5.3. Secondary Endpoint

- Prostate-specific antigen (PSA) response rate (as defined by PCWG2).

5.3.1. Definition

Prostate-specific antigen (PSA) response rate is defined as the proportion of subjects who achieved at least a 50% decline in PSA value from baseline assessed by a central laboratory according to PCWG2 criteria. The PSA response will be confirmed by a central laboratory measurement obtained 4 or more weeks later. Placebo group subjects who do not have PSA response at the time of treatment switching will be considered as non-responder.

5.3.2. Analysis Methods

PSA response rate will be summarized by treatment group. The relative risk (treatment:control) will be reported along with the associated 2-tailed 95% CIs. The two treatment groups will be compared using the stratified Mantel-Haenszel test if data warranted. Fisher's exact test may be used if the expected counts in some cells are small.

Summary tables and waterfall plots describing change in PSA relative to baseline will be reported at 12 weeks (or earlier for those who switch/discontinue study treatment prior to 12 weeks). PSA response by Week 12 will also be reported by treatment group.

5.4. Exploratory Efficacy Variables

Efficacy Variables

- Metastasis-free survival (MFS)
- Overall survival (OS)
- Time to symptomatic progression

5.4.1. Definition

- Metastasis-free survival (MFS) defined as the time from randomization to first evidence of investigator determined radiographically detectable bone or soft tissue distant metastasis (simply referred to as "metastasis" from this point forward) using the Response Evaluation Criteria in Solid Tumors (RECIST v1.1) or death due to any cause (whichever occurs earlier).[2] For new bone lesions detected on bone scans, a second imaging modality (e.g., CT or MRI) will be required to confirm progression.
- Overall survival (OS) defined as the time from randomization to date of death from any cause
- Time to symptomatic progression will be defined as the time from randomization to documentation in the CRF of any of the following (whichever occurs earlier) + 1 day:
  - Development of a skeletal-related event (SRE): pathologic fracture, spinal cord compression, or need for surgical intervention or radiation therapy to the bone.
  - Pain progression or worsening of disease-related symptoms requiring initiation of a new systemic anti-cancer therapy.
  - Development of clinically significant symptoms due to loco-regional tumor progression requiring surgical intervention or radiation therapy.

  Adverse event, concomitant medication, or survival follow-up CRFs may also be the source of these findings.

  Time to symptomatic progression for patients who do not experience any of the events described above will be censored on the date on which they were last known to be event-free.

5.4.2. Analysis Methods

Exploratory time to event variables will be analyzed descriptively only for apalutamide group. KM method will be applied to graphically describe the distribution. Median event times, the 6-month,

1-year and 2-year event free rates, as appropriate, and their 2-sided 95% confidence intervals will be provided.

6. SAFETY

Safety data will be analyzed using safety analysis set. The safety parameters to be evaluated are the incidence, intensity, and type of adverse events (AE), vital signs, ECG, and clinical laboratory results.

Unless otherwise specified, no statistical inference will be performed in analyzing the safety data.

In this study, control group subjects without distant metastasis will switch to treatment with apalutamide plus ADT after completion of 5 cycles of placebo + ADT treatment (handling by the IWRS). Subjects who have documented PSA progression prior to completion of 5 cycles of study treatment, will cross over to apalutamide at the time of PSA progression. As such, safety data will be presented by the following treatment groups as appropriate.

**Placebo [a]**	**Apalutamide**	**Placebo to Apalutamide [b]**	**Apalutamide Treated [c]**

[a] Placebo subjects treated with Placebo + ADT before cross-over
[b] Placebo subjects treated with apalutamide + ADT after cross-over
[c] Subjects treated with apalutamide + ADT including subjects from original apalutamide arm and subjects crossed over from placebo arm

6.1. Adverse Events

Subjects will be assessed for adverse events while on the study. Adverse events (AEs) will be graded according to the NCI Common Terminology Criteria for Adverse Events (CTCAE) version 4.03 and coded to preferred term and system organ class (SOC) using the MedDRA version 20.0 or later. All AEs reported on or after the date of first dose until 30 days (inclusive) after the last dose of study drug will be considered treatment-emergent and will be summarized by treatment group.

For each treatment group, AE incidence rates will be summarized with frequency and percentage by SOC and preferred term, with all subjects treated in that treatment group as the denominator, unless otherwise specified. In addition, AE incidence rates will also be summarized by severity and relationship to study drug. Treatment-related AEs are those judged by the Investigator to be at least possibly related to the blinded study drug. AEs with missing severity or relationship to study drug will be classified as severe and treatment-related, respectively. Subjects with multiple occurrences of events will only be counted once at the maximum severity to study drug for each preferred team, SOC. Deaths that occur within 30 days after the last dose of study drug are defined as on-study deaths.

Summary tables of the following AEs will be provided:

- Overall summary of AEs: the number and percentage of subjects who experienced any AE including COVID-19, number of subjects with Grade 3/4 AEs, any serious adverse event

(SAE) including COVID-19, any treatment-related AE, treatment related Grade 3/4 AE, any treatment-related SAE, AE leading to treatment discontinuation including COVID-19, related AE leading to treatment discontinuation, AE leading to death including COVID-19, related AE leading to death, and all deaths within 30 days of last dose

- All AEs by SOC and preferred term
- All AEs by SOC, preferred term, and toxicity grade
- Grades 3 or 4 AEs by SOC and preferred term
- Grades 3 or 4 AEs by decreasing frequency of preferred term
- Treatment-related AEs by SOC and preferred term
- Treatment-related AEs by SOC, preferred term, and toxicity grade
- Treatment-related AEs by decreasing frequency of preferred term
- Treatment-related Grades 3 or 4 AEs by SOC and preferred term
- Treatment-related Grades 3 or 4 AEs by decreasing frequency of preferred term
- All AEs that led to death by SOC and preferred term
- All AEs that led to study drug discontinuation by SOC and preferred term. Study drug discontinuation will be determined from the End of Treatment CRF (where reason for termination is “Adverse Event”) and the specific AE will be determined from the AE eCRF page (where action taken is “Drug Withdrawn”)
- All AEs that led to study drug discontinuation by SOC, preferred term, and toxicity grade
- All AEs that lead to study drug dose modification by SOC and preferred term
- All SAEs by SOC and preferred term
- All SAEs by SOC, preferred term, and toxicity grade
- Deaths will be summarized by time period (on-study vs. during follow-up) and cause of death

Subject listings of all Grades 3 or 4 AEs, all SAEs, AEs that led to study drug discontinuation, dose modification, and all deaths will be provided as well.

Narratives will be written for the following subjects in the final clinical study report:

- Subjects who die ≤ 30 days after the last dose of study drug
- Subjects who discontinue study drug due to adverse events
- Subjects who have a serious adverse event
- Subjects who experience a seizure
- Grade 3 or higher adverse events of special interest

6.2. Clinical Laboratory Tests

The number and percentage of subjects with abnormal laboratory values will be summarized. The number and percentage of subjects with parameters with predefined NCI-CTCAE (version 4.03)

toxicity grades of ≥ 3 may also be summarized. For selected key laboratory measurements, changes in toxicity grade from baseline to the worst grade experienced by the subject during the Treatment Phase and the Study will be summarized using shift tables.

A listing of subjects who develop toxicities of Grade ≥ 3 will be provided for each laboratory parameter.

6.3. Vital Signs and Physical Examination Findings

Each vital sign (temperature, blood pressure (systolic and diastolic), respiration rate, and heart rate) at baseline will be summarized and presented by treatment group. The number and percentage of subjects with values beyond clinically important limits (defined in Table 2) will be summarized.

Table 2 Clinically Significant Abnormality For Vital Signs

Parameter	Criteria
Systolic blood pressure	• Absolute result > 160 mmHg and increase from baseline of > 20 mmHg<br>• Absolute result < 90 mmHg and decrease from baseline of > 20 mmHg
Diastolic blood pressure	• Absolute result > 100 mmHg and increase from baseline of > 10 mmHg<br>• Absolute result < 50 mmHg and decrease from baseline of > 10 mmHg
Weight	• 5 - < 10% weight loss from baseline<br>• 10 - < 20% weight loss from baseline<br>• >= 20% weight loss from baseline

Abnormal findings in physical examination will be recorded and summarized as AEs.

6.4. Electrocardiogram

Electrocardiogram parameters will be summarized using descriptive statistics at baseline.

7. PHARMACOKINETICS/PHARMACODYNAMICS

Descriptive statistics including arithmetic mean, SD, geometric mean, coefficient of variation, median, range will be used to summarize plasma apalutamide and N-desmethyl apalutamide concentrations at each sampling time point.

Individual PK parameters and exposure information of apalutamide and N-desmethyl apalutamide will be derived using population PK modelling. Exposure-response (ER) analysis between exposure and selective clinical endpoint will be explored if deemed necessary and with sufficient data. Details will be given in a separate population PK and ER analysis plan. Results of these analysis will be provided in a separate report.

REFERENCES

1. Scher HI, Halabi S, Tannock I, et al. Design and end points of clinical trials for patients with progressive prostate cancer and castrate levels of testosterone: Recommendations of the prostate cancer clinical trials working group. *J Clin Oncol* 2008; 26:1148-1159.
2. Kaplan EL and Meier P. Nonparametric estimation from incomplete observations. J Am Stat Assoc 1958; 53:457-81
3. Eisenhauer EA, Therasse P, Bogaerts J, et al. New response evaluation criteria in solid tumours: Revised RECIST guideline (version 1.1). Eur J Cancer 2009; 45:228-47
