# Supplementary material for: A randomized study of apalutamide in Chinese patients with non-metastatic castration-resistant prostate cancer
Source: iScience. 2025 Jul 18;28(8):113166. doi: 10.1016/j.isci.2025.113166 (PMC12355417; doi:10.1016/j.isci.2025.113166)
Supplement: Data S1. CONSORT checklist, study protocol, and study statistical analysis plan [file mmc2.zip › Study Protocol.pdf]

Janssen Research & Development *

Protocol Title
A Multicenter, Randomized, Double-Blind, Placebo-Controlled, Phase IV Study of Apalutamide in Chinese Participants with Non-Metastatic Castration-Resistant Prostate Cancer (NM-CRPC)

Protocol 56021927PCR4007; Phase IV

AMENDMENT 3

JNJ-56021927 (apalutamide)

*Janssen Research & Development is a global organization that operates through different legal entities in various countries. Therefore, the legal entity acting as the sponsor for Janssen Research & Development studies may vary, such as, but not limited to Janssen Biotech, Inc.; Janssen Products, LP; Janssen Biologics, BV; Janssen-Cilag International NV; Janssen, Inc; Janssen Pharmaceutica NV; Janssen Sciences Ireland UC; or Janssen Research & Development, LLC. The term “sponsor” is used throughout the protocol to represent these various legal entities; the sponsor is identified on the Contact Information page that accompanies the protocol.

Status: Approved
Date: 20 June 2022
Prepared by: Janssen Research & Development, LLC
EDMS number: EDMS-ERI-177074070, 4.0

GCP Compliance: This study will be conducted in compliance with Good Clinical Practice, and applicable regulatory requirements.

Confidentiality Statement
The information in this document contains trade secrets and commercial information that are privileged or confidential and may not be disclosed unless such disclosure is required by applicable law or regulations. In any event, persons to whom the information is disclosed must be informed that the information is privileged or confidential and may not be further disclosed by them. These restrictions on disclosure will apply equally to all future information supplied to you that is indicated as privileged or confidential.

PROTOCOL AMENDMENT SUMMARY OF CHANGES TABLE

DOCUMENT HISTORY	
**Document**	**Date**
COVID-19 Appendix (v 1.0)	20 June 2022
Amendment 3	20 June 2022
Amendment 2	20 February 2020
Amendment 1	7 June 2019
Original Protocol	13 February 2019

Amendment 3 (20 June 2022)

Overall Rationale for the Amendment: The overall reason for the amendment is to 1) change the number of participants to 75 for the final analysis based on CDE consultation, considering that the enrollment of study PCR4007 will become more and more challenging at current stage from both ethical and operational perspectives and the sample size from 111 to 75 participants is still statistically valid 2) remove the interim analysis and revise the role of Independent Data Monitoring Committee (IDMC) from reviewing interim analysis results and making recommendations to reviewing the final efficacy and safety results.

Section Number and Name	Description of Change	Brief Rationale
1.1. Synopsis: OVERALL DESIGN; NUMBER OF PARTICIPANTS; STATISTICAL METHODS: Interim Analysis<br>1.2. Schema<br>4.1. Overall Design<br>4.4. End of Study Definition<br>9.4. Interim Analysis<br>10.2. Appendix 2—COMMITTEES STRUCTURE	• Changed the appropriate number of participants to be enrolled from 111 to 75.<br>• Removed the original section for interim analysis and clarified that no interim analysis is planned for this study.<br>• Changed the role of IDMC from reviewing interim analysis result and making recommendations to reviewing the final efficacy and safety results.<br>• Removed the independent analysis team which was planned to be assembled by sponsor to prepare and provide study data to the IDMC.<br>• Removed the recommendation of IDMC on the study termination beforehand.	To make change as interim analysis is not applicable and 75 participants will be used for final analysis.
1.1. Synopsis: OVERALL DESIGN<br>4.1. Overall Design<br>6.3. Measures to Minimize Bias: Randomization and Blinding: Intervention Allocation: Blinding	• Clarified unblinding will occur "until completion of the study or the sponsor decides to unblind the study at final analysis".<br>• Revised the circumstance of unblinding and decoding of randomization to add "or the sponsor decides to unblind the study at final analysis" and remove the IDMC's intervention.	• To further clarify the conditions of unblinding.<br>• To make revision considering IDMC recommendation for unblinding and IDMC review for interim analysis will not be applicable.
1.1. Synopsis: STATISTICAL METHODS: Sample Size Determination<br>4.2.1. Study-	• Recalculated that approximately 75 participants will provide approximately 28 TTPP events at the time of the final analysis.<br>• Recalculated only 1-2 participants in this	• The event number was estimated based on the number of participants (75), the upper limit of 95% CI in the TTPP HR results of East Asian from SPARTAN (0.11) and accrual period (~ 32 months).

**Section Number and Name**	**Description of Change**	**Brief Rationale**
Specific Ethical Design Considerations<br>9.1. Sample Size Determination	study are expected to have metastatic disease at the first post-baseline scan without prior PSA progression given one third of 75 Chinese participants (about 25 participants) are to be randomly assigned to placebo arm.<br>• Revised the accrual period from 38 months to 32 months and the approximate study duration for the TTPP final analysis from 43 months to 37 months.	• 32-month accrual period was estimated based on the date of FPI (2020.2) and LPI (2022.10), and the study duration was redefined accordingly.
1.1. Synopsis: Efficacy Analysis<br>9.3.1. Efficacy Analysis	• Changed the time point to estimate the PSA progression-free rate from "6 months, 1-year and 2-year" to "at selected timepoint".	• To make revision considering the censoring rule for placebo arm.
1.3. Schedule of Activities (SoA)-footnote 1<br>4.1. Overall Design	Specified the Prescreening Phase will be ended with the wording "till the last participant screening is successful".	• To define when the prescreening is not necessary.
2. INTRODUCTION	Updated the approved countries for ERLEADA from 40 to 75.	To update approved status for ERLEADA.
2.2. Background	• Updated the clinical study status (ongoing or completed) regarding 56021927PCR3003.<br>• Updated the final efficacy analysis data of Study ARN-509-003 (SPARTAN), including the secondary efficacy analysis (time to symptomatic progression, OS, and time to initiation of cytotoxic chemotherapy), PFS2, and patient-reported outcome results.<br>• Updated the safety data of Study ARN-509-003 (SPARTAN), including study disposition, treatment exposure, TEAE, TEAE leading to death/treatment discontinuation/reduction, SAE, and AE of special interest.	To align with the study data in current IB and Study ARN-509-003 (SPARTAN) Clinical Study Report addendum.
2.2.1. Androgen Deprivation Therapy	• Added specification that GnRHa will be provided by the sponsor and should be used "during the treatment phase".	To define the period of GnRHa provided and used.
4.2. Scientific Rationale for Study Design<br>4.2.1. Study-Specific Ethical Design Considerations	• Clarified there were no approved therapies "at the initiation of this study" for the treatment of patients with NM-CRPC, and added "Since then, 2 other products have been conditionally approved or approved for the treatment of patients with NM-CRPC".<br>• Removed the description "ADT alone is the appropriate control in Chinese NM-CRPC patients as continuous administration of ADT constitutes as part of the current *de facto* community standard practice".	To update and show the unmet need at the initiation of this study.
8.3.1. Time Period and Frequency for	Removed safety report form of the eCRF to be used to record SAEs.	To align with the current practice.

**Section Number and Name**	**Description of Change**	**Brief Rationale**
Collecting Adverse Event and Serious Adverse Event Information		
Throughout the protocol	Minor grammatical, formatting, or spelling changes were made.	Minor errors were noted

TABLE OF CONTENTS

PROTOCOL AMENDMENT SUMMARY OF CHANGES TABLE **2**

TABLE OF CONTENTS **5**

LIST OF IN-TEXT TABLES AND FIGURES **7**

1. PROTOCOL SUMMARY **8**
1.1. Synopsis 8
1.2. Schema 13
1.3. Schedule of Activities (SoA) 14

2. INTRODUCTION **17**
2.1. Study Rationale 18
2.2. Background 18
2.2.1. Androgen Deprivation Therapy 24
2.3. Benefit/Risk Assessment 24

3. OBJECTIVES AND ENDPOINTS **25**

4. STUDY DESIGN **26**
4.1. Overall Design 26
4.2. Scientific Rationale for Study Design 28
4.2.1. Study-Specific Ethical Design Considerations 29
4.3. Justification for Dose 29
4.4. End of Study Definition 29

5. STUDY POPULATION **30**
5.1. Inclusion Criteria 30
5.2. Exclusion Criteria 32
5.3. Screen Failures 34

6. STUDY INTERVENTION **34**
6.1. Study Interventions Administered 34
6.2. Preparation/Handling/Storage/Accountability 35
6.3. Measures to Minimize Bias: Randomization and Blinding 36
6.4. Study Intervention Compliance 37
6.5. Concomitant Therapy 38
6.5.1. Suggested Therapy 38
6.5.2. Prohibited Therapy 39
6.5.3. Restricted Concomitant Therapy 40
6.6. Dose Modification 40
6.6.1. Dose Modifications for Rash and Rash Management 41
6.7. Long-term Follow-up 42

7. DISCONTINUATION OF STUDY INTERVENTION AND PARTICIPANT DISCONTINUATION/WITHDRAWAL **43**
7.1. Discontinuation of Study Intervention 43
7.2. Participant Discontinuation/Withdrawal From the Study 44
7.3. Lost to Follow-up 44

8. STUDY ASSESSMENTS AND PROCEDURES **44**
8.1. Efficacy Assessments 45
8.2. Safety Assessments 46
8.2.1. Physical Examination 46
8.2.2. Vital Signs 47
8.2.3. Electrocardiogram (ECG) 47

8.2.4. Clinical Safety Laboratory Assessments ... 47
8.2.5. ECOG Performance Status ... 48
8.3. Adverse Events and Serious Adverse Events ... 48
8.3.1. Time Period and Frequency for Collecting Adverse Event and Serious Adverse Event Information ... 49
8.3.2. Method of Detecting Adverse Events and Serious Adverse Events ... 49
8.3.3. Follow-up of Adverse Events and Serious Adverse Events ... 49
8.3.4. Regulatory Reporting Requirements for Serious Adverse Events ... 49
8.3.5. Pregnancy ... 49
8.4. Treatment of Overdose ... 50
8.5. Pharmacokinetics ... 50
8.5.1. Evaluations ... 50
8.5.2. Analytical Procedures ... 50
8.5.3. Pharmacokinetic Parameters and Evaluations ... 50
8.6. Medical Resource Utilization and Health Economics ... 51

9. STATISTICAL CONSIDERATIONS ... 51
9.1. Sample Size Determination ... 51
9.2. Populations for Analyses ... 51
9.3. Statistical Analyses ... 52
9.3.1. Efficacy Analyses ... 52
9.3.2. Safety Analyses ... 53
9.3.3. Other Analyses ... 54
9.4. Interim Analysis ... 54
9.4.1. Independent Data Monitoring Committee ... 54

10. SUPPORTING DOCUMENTATION AND OPERATIONAL CONSIDERATIONS ... 55
10.1. Appendix 1: Abbreviations ... 55
10.2. Appendix 2: Regulatory, Ethical, and Study Oversight Considerations ... 57
REGULATORY AND ETHICAL CONSIDERATIONS ... 57
FINANCIAL DISCLOSURE ... 60
INFORMED CONSENT ... 60
DATA PROTECTION ... 61
COMMITTEES STRUCTURE ... 62
PUBLICATION POLICY/DISSEMINATION OF CLINICAL STUDY DATA ... 62
DATA QUALITY ASSURANCE ... 63
CASE REPORT FORM COMPLETION ... 64
SOURCE DOCUMENTS ... 64
MONITORING ... 65
ON-SITE AUDITS ... 66
RECORD RETENTION ... 66
STUDY AND SITE CLOSURE ... 67
10.3. Appendix 3: Adverse Events: Definitions and Procedures for Recording, Evaluating, Follow-up, and Reporting ... 68
ADVERSE EVENT DEFINITIONS AND CLASSIFICATIONS ... 68
ATTRIBUTION DEFINITIONS ... 69
SEVERITY CRITERIA ... 69
SPECIAL REPORTING SITUATIONS ... 70
PROCEDURES ... 70
CONTACTING SPONSOR REGARDING SAFETY ... 72
PRODUCT QUALITY COMPLAINT HANDLING ... 72
10.4. Appendix 4: Liver Safety: Suggested Actions and Follow-up Assessments ... 73
10.5. Appendix 5: ECOG-PS Score ... 77
10.6. Appendix 6: Protocol Amendment History ... 78

11. REFERENCES ... 83

INVESTIGATOR AGREEMENT ... 84

LIST OF IN-TEXT TABLES AND FIGURES

TABLES

Table 1: Summary of Secondary Efficacy Analysis (Study ARN-509-003 [SPARTAN])[4] 20
Table 2: Description of Interventions 35
Table 3: Dose Modifications of Apalutamide/Placebo (except for rash, if rash occurs, see Table 4)... 41
Table 4: Dose-modification and Management of Drug-related Rash 42

FIGURES

Figure 1: Kaplan-Meier Plot of Blinded Independent Central Review (BICR) Metastasis-Free Survival (MFS); Intent-to-treat Population (Study ARN-509-003 [SPARTAN])[4] 19
Figure 2: Kaplan-Meier Plot of Time to PSA Progression; Intent-to-treat Population (Study ARN-509-003 [SPARTAN])[4]... 21

1. PROTOCOL SUMMARY

1.1. Synopsis

A Multicenter, Randomized, Double-Blind, Placebo-Controlled, Phase IV Study of Apalutamide in Chinese Participants with Non-Metastatic Castration-Resistant Prostate Cancer (NM-CRPC)

Apalutamide (also known as ARN-509 and JNJ-56021927) is an orally available, small molecule, non-steroidal potent and selective antagonist of the androgen receptor (AR) (anti-androgen). It is currently being developed for the treatment of prostate cancer. The current study is designed to validate the findings of a global study (ARN-509-003 [SPARTAN]) among Chinese patients.

OBJECTIVES AND ENDPOINTS

Objectives	Endpoints
**Primary**	
To compare the improvement in TTPP (as defined by PCWG2) of apalutamide versus (vs) placebo in Chinese participants with high-risk NM-CRPC*	TTPP
**Secondary**	
To evaluate the safety and tolerability of apalutamide	AEs and laboratory abnormalities
To compare the PSA response rate in Chinese participants with high-risk NM-CRPC treated with apalutamide vs placebo*	PSA response rate
To evaluate the PK of apalutamide and N-desmethyl apalutamide	PK: Predose and 2h postdose plasma concentrations after single dose and at steady-state.

Abbreviations: AE: adverse event; NM-CRPC: non-metastatic castration-resistant prostate cancer; PK: pharmacokinetic; PCWG2: prostate cancer working group 2, PSA: prostate-specific antigen; TTPP: time to PSA progression.
*Participants randomized to placebo arm will be crossed over to receive apalutamide treatment either after completion of a maximum 5 cycles of placebo treatment or at the time of having documented PSA progression, whichever comes earlier.

Hypothesis

Participants receiving treatment with apalutamide in conjunction with androgen deprivation therapy (ADT), will have a longer time to prostate-specific antigen (PSA) progression (TTPP) compared with participants receiving placebo plus ADT.

OVERALL DESIGN

This is a Phase IV, randomized, double-blind, placebo-controlled, multicenter study evaluating the efficacy and safety of apalutamide vs placebo in male Chinese participants with high-risk NM-CRPC.

A target of approximately 75 Chinese participants will be randomly assigned in a 2:1 ratio in this study to receive apalutamide or matching placebo. Participants will be stratified by PSA doubling time (PSADT) (>6 months vs ≤6 months)

The study will consist of 4 phases:

- Prescreening Phase (optional)
- Screening Phase (≤35 days prior to randomization)
- Treatment Phase (starting on Day 1 of Cycle 1 until disease progression/unacceptable toxicity, withdrawal of consent, death or termination of the study)
- Follow-up Phase (starting after end-of-treatment visit until death, withdrawal of consent for study participation, or termination of the study).

Apalutamide or matched placebo will be administered orally on a continuous daily dosing schedule at a starting dose of 240 mg per day. Androgen deprivation therapy with gonadotrophin-releasing hormone agonists (GnRHa) will be continued for all patients who have not been surgically castrated.

Each treatment cycle will consist of 28 days. Participants randomized to placebo who do not have any evidence of distant metastasis (according to imaging result at Cycle 5 Day 1, as well as at any other relevant unscheduled visit) will be crossed over and start receiving active therapy of apalutamide after completion of 5 cycles of placebo plus ADT treatment. Participants who have documented PSA progression prior to completion of 5 cycles of study treatment, will cross over to apalutamide at the time of PSA progression. Any participants who develop distant metastasis at any time on study will discontinue study treatment, and may receive subsequent therapy as per standard of care. Around half of the participants in the placebo arm are expected to have documented PSA progression after completion of 5 cycles treatment, as estimated from the ARN-509-003 (SPARTAN) study data, and would therefore, allow sufficient time for the evaluation of TTPP and comparison between apalutamide and placebo.

Prostate-specific antigen will be collected and analyzed by a central laboratory but investigators, participants, and the study team will be blinded to the results until completion of the study or the sponsor decides to unblind the study at final analysis. The central laboratory will analyze the individual PSA data, collected at baseline and post-baseline obtained within the first 5 cycles to flag out documented PSA progression per PCWG2 and the data will be entered into the interactive web response system (IWRS) to trigger the cross-over by an independent team not affiliated with the study team. The cross-over of control arm participants will be handled by IWRS to maintain the blinding of original treatment assignment of individual participant.

Participants will be followed for safety and efficacy as per schedule of activities and will remain on study treatment until development of distant metastases as assessed by investigators or the development of unacceptable toxicity or the end of study defined as metastasis-free survival (MFS) events observed in approximately 60% of the study population or 3 years after the final analysis for the primary endpoint (TTPP), whichever comes first.

All participants will be monitored for safety during the Screening and Treatment Phases, and for up to 30 days after the last dose of study treatment. An End-of-Treatment (EoT) Visit will occur approximately 30 days of the last dose of study treatment. The Posttreatment Follow-up Phase will begin after the EoT visit. For participants entering Posttreatment Follow-up Phase with evidence of distant metastasis, they will be contacted every 4 months (±7 days) to obtain survival status, recording of development of symptomatic progression, initiation of any new systemic anticancer therapies until death, withdrawal of consent for study participation, or termination of the study. For participants entering Posttreatment Follow-up Phase without evidence of distant metastasis, Posttreatment Follow-up visits including disease assessment (computed tomography [CT] or magnetic resonance imaging [MRI] plus bone scans) will continue every 16 weeks (±7 days) until the documentation of disease progression, then participants will be contacted every 4 months (±7 days) to obtain survival status, recording of development of symptomatic progression, initiation of any new systemic anticancer therapies until death, withdrawal of consent for study participation, or termination of the study.

An Independent Data Monitoring Committee (IDMC) will be commissioned for this study to review the efficacy and safety results of final analysis. The IDMC will consist of at least 1 medical oncologist or urologist or both and at least 1 statistician.

NUMBER OF PARTICIPANTS

A target of 75 Chinese male participants will be randomly assigned in a 2:1 ratio in this study to receive apalutamide or matching placebo with ADT.

INTERVENTION GROUPS AND DURATION

Each treatment cycle will consist of 28 days. Participants will be randomly assigned in a 2:1 ratio:

- Investigational group will receive apalutamide 240 mg (4×60 mg tablets) daily.
- Control group will receive placebo daily from Cycle 1 Day 1. Participants who do not have distant metastasis will switch to treatment with apalutamide after completion of 5 cycles of placebo treatment (handling by the IWRS). Participants who have PSA progression prior to completion of 5 cycles of study treatment, will cross over to apalutamide at the time of PSA progression.

Description of Interventions

**Arm Name**	Arm A	Arm B
**Intervention Name**	Apalutamide	Placebo
**Type**	A drug	A matching placebo to active drug
**Dose Formulation**	Immediate release tablet	Immediate release tablet
**Unit Dose Strength(s)**	4×60 mg	4×60 mg
**Dosage Level(s)**	240 mg daily	240 mg daily
**Route of Administration**	Oral	Oral
**Use**	Experimental	Placebo-Comparator
**Packaging and Labeling**	120-ct, 160°cc HDPE bottles with CRC and include desiccant.	120-ct, 160°cc HDPE bottles with CRC and include desiccant.
**Child-resistant packaging**	Yes	Yes
**Delivery instructions**	Apalutamide: QD without regard to food	Apalutamide: QD without regard to food
**Names/Aliases**	JNJ-56021927	NA

ADT: androgen deprivation therapy, being either surgical castration or gonadotrophin-releasing hormone agonists (GnRHa), will be continued as background therapy for all participants. ADT with GnRHa will be continued for all patients who have not been surgically castrated.; CRC: child-resistant closures; HDPE: high density polyethylene; QD: once daily.

EFFICACY EVALUATIONS

The efficacy evaluations include the following:

- Serum PSA evaluation (for TTPP, as defined by PCWG2)
- Disease assessment (CT or MRI, bone scans)
- Survival status, subsequent anticancer therapy, and skeletal-related events (SREs).

Serum PSA evaluation (for TTPP, as defined by PCWG2): During the treatment phase, PSA will be analyzed at the central laboratory. Results will be kept blinded to the participants, the Investigators, and the study team, in order to preserve the double-blind nature of this study. On Cycle 1 Day 1 only, the PSA evaluation does not need to be repeated if the Screening tests were done within 4 days of Cycle 1 Day 1. For all participants, at the time of full unblinding of the study, PSA data submitted to the central laboratory will no longer be blinded.

Disease assessment (CT or MRI, bone scans): Disease assessments will be performed at baseline (screening: scans obtained prior to signing informed consent as part of the patient’s standard of care may be allowed, provided the timing of the scans fall within the screening window), and at 16-week intervals thereafter. Imaging studies will include a CT scan of the chest, abdomen, and pelvis, plus a bone scan. At Screening, there will be an additional CT of the brain to rule out the presence of central nervous system (CNS) metastases. For new bone lesions detected on bone scans, a second imaging modality (eg, CT or MRI) will be required to confirm progression. The same method of assessment and the same technique should be used at Screening and during follow-up. Intravenous (IV) contrast is required when not

medically contraindicated. Participants who have a contraindication to IV contrast may have MRI exams of the brain, abdomen, and pelvis performed in lieu of CTs and a non-contrast CT of the chest. Tumor evaluation by positron emission tomography (PET) scan or by ultrasound or by prostate-specific membrane antigen (PSMA)-PET will not replace for standard chest, abdomen, and pelvis CT or MRI scans or bone scans as required by the study, but the CT portion of a PET/CT with sufficient quality which could meet the study requirement may be submitted in lieu of a dedicated CT. The radiographic disease progression (appearance of distant metastasis) will be determined by the investigator. Participants who discontinue treatment before documented disease progression should continue with disease assessments during Posttreatment Follow-up Phase every 16 weeks (±7 days) until disease progression.

Survival status, subsequent anticancer therapy, and SREs: These assessments will be performed during the Treatment and Posttreatment Follow-up Phase.

SAFETY EVALUATIONS

Adverse events (AEs) will be reported and followed by the investigator. Any clinically relevant changes occurring during the study must be recorded on the electronic case report form (eCRF). Any clinically significant abnormalities persisting at the end of the study/early withdrawal will be followed by the investigator until resolution or until a clinically stable condition is reached.

The study will include the following evaluations of safety and tolerability according to the time points as scheduled: physical examination, vital signs, electrocardiogram (ECG), clinical safety assessments (including laboratory analyses), and Eastern Cooperative Oncology Group (ECOG) performance status (PS).

STATISTICAL METHODS

Sample Size Determination

The pivotal ARN-509-003 (SPARTAN) study suggested that East Asian participants (and study participants as a whole) treated with ADT only will have a median TTPP of 3.7 months. It is estimated that approximately 75 participants will provide approximately 28 TTPP events at the time of the final analysis (approximately 5 months after last participant receiving first dose) which yields a power exceeding 95% for detecting a hazard ratio (HR) of 0.11 (median TTPP of 3.7 months for the placebo arm versus 33.6 months for the apalutamide arm) at a 2-tailed level of significance of 0.05.

Efficacy Analysis

The final analysis of TTPP will be performed at approximately 5 months after last participant receiving the first dose. Time to PSA progression will be tested using Stratified log-rank test, stratified by PSADT (>6 months vs ≤6 months), at a 2-tailed significance level of 0.05. Kaplan-Meier method will be used to estimate the median TTPP, as well as the PSA progression-free rate at selected timepoints, and their 95% CIs. The estimation of HR and its 95% CI will be based on the stratified Cox proportional-hazard model. Non-stratified log-rank tests will also be provided as sensitivity analysis.

Prostate-specific antigen response rate will be evaluated using the Mantel-Haenszel test or the exact test if the frequencies are small. Time-to-event-based exploratory analyses (MFS, overall survival [OS], and time to symptomatic progression) will be performed descriptively, considering the early cross-over of control arm and the expected long medians for apalutamide arm suggested by study ARN-509-003 (SPARTAN).

Pharmacokinetic Analyses

Descriptive statistics will be used to summarize plasma apalutamide and its active metabolite N-desmethyl apalutamide concentrations at each sampling time point. Population PK analysis of plasma

concentration-time data of apalutamide and N-desmethyl apalutamide will be performed using nonlinear mixed effects modeling. The population PK analysis results will be presented in a separate report.

Exposure-Response Analyses

Exposure-response analysis between exposure and selective clinical endpoint will be explored if deemed necessary and with sufficient data. The exposure-response analysis results will be presented in a separate report.

Safety Analyses

Participants who receive at least 1 dose of study drug will be analyzed for safety. The safety parameters to be evaluated are the incidence, intensity, and type of AEs, vital signs measurements, and clinical laboratory results. Exposure to study drug and reasons for discontinuation of study treatment will be tabulated.

Interim Analysis

No interim analysis is planned for this study.

1.2. Schema

Figure 1: Schematic Overview of the Study

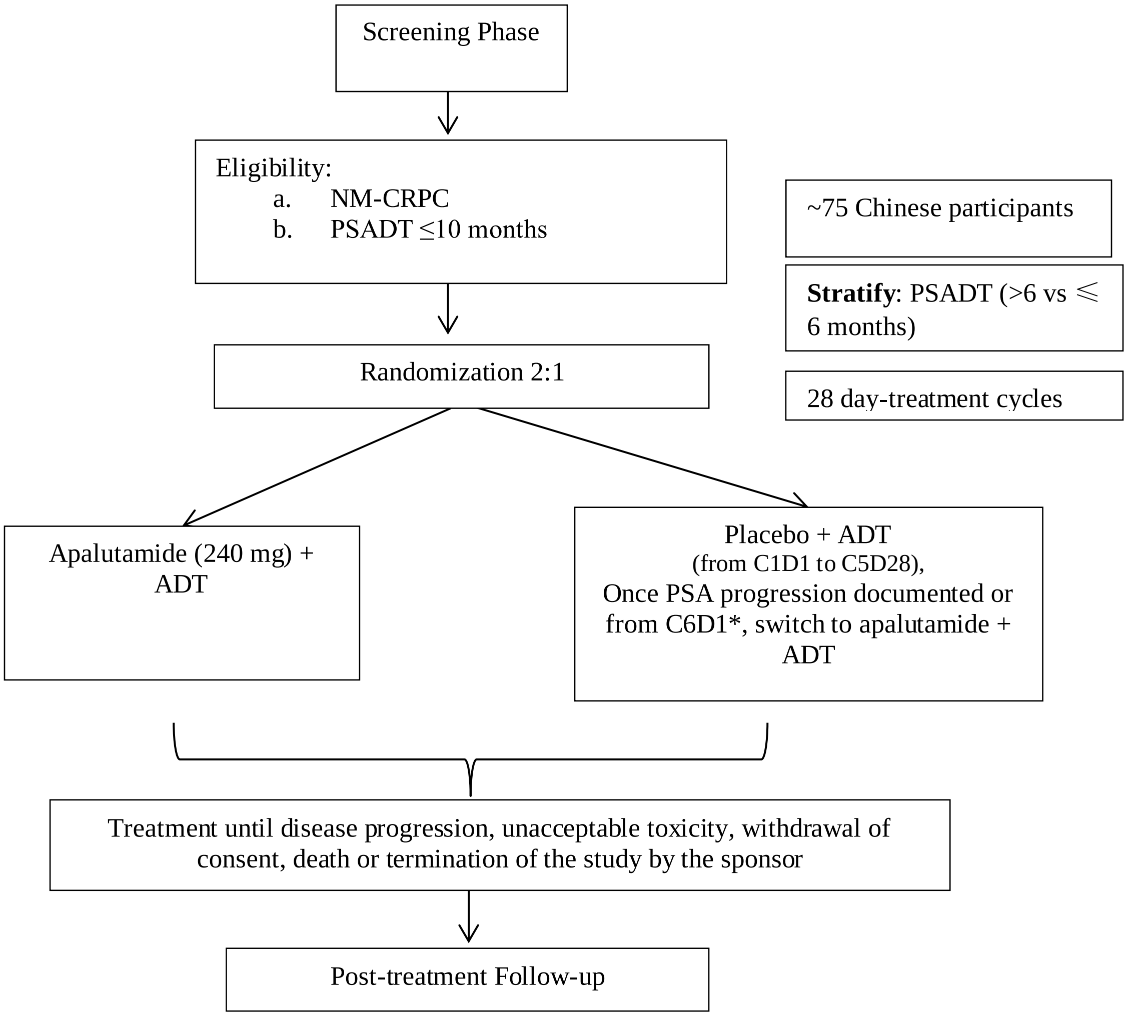

Abbreviations: ADT: androgen deprivation therapy; C: cycle; D: day; NM-CRPC: non-metastatic castration-resistant prostate cancer; PSA: prostate-specific antigen; PSADT: PSA doubling time.

Notes:

*Participants randomized to the placebo arm who do not have evidence of distant metastasis (according to imaging result at Cycle 5 Day 1 as well as any other relevant unscheduled visit) will be crossed over and start receiving active therapy with apalutamide after completion of 5 cycles of placebo plus ADT treatment. Participants who have documented PSA progression prior to completion of 5 cycles of study treatment, will cross over to apalutamide at the time of documented PSA progression. Participants who develop distant metastatic disease at any time of the study will discontinue study treatment.

1.3. Schedule of Activities (SoA)

	Comments	Prescreening[1]	Screening	Treatment Phase (One cycle = 4 weeks [28 days])[2]				Posttreatment follow-up[3]
**Activities and Forms to be Completed**			**≤35 Days Prior to Randomization**	**Cycle 1 Day 1**	**D1 C1-C6, D1 of every 2 cycles starting at C7 to C13, then D1 of every 4 cycles, unless otherwise specified**	**Every 4 cycles**	**End-of-Treatment (~ 30 days of the last dose of study treatment)**	
**Screening**								
Informed Consent		X	X					
Medical/Oncological History			X					
Inclusion/Exclusion Criteria[4]			X					
Randomization	Participants will be randomly assigned to the treatment arms; randomize no more than 1 week before dosing.		X					
**Study Drug Administration**								
Apalutamide/Matched Placebo Administration[5]	C1D1 must be within 1 week of randomization			X	X			
Study Drug Compliance					X		X	
**Laboratory Studies**								
Hematology			X	X	X		X	
Serum Chemistry panel/tests			X	X	X		X	
Testosterone			X	X		X	X	
Thyroid Stimulating Hormone[6]			X	X		X	X	
Fasting Lipid Panel			X	X		X	X	
HBsAg, anti-HCV			X					
HBV-DNA and HCV-RNA			X[7]					
**Efficacy**								
Serum PSA Evaluation	During treatment phase: Blinded; submit to central lab	X	X	X	X[8]		X	
CT brain			X[9]					

	Comments	Prescreening[1]	Screening	Treatment Phase (One cycle = 4 weeks [28 days])[2]				Posttreatment follow-up[3]
**Activities and Forms to be Completed**			**≤35 Days Prior to Randomization**	**Cycle 1 Day 1**	**D1 C1-C6, D1 of every 2 cycles starting at C7 to C13, then D1 of every 4 cycles, unless otherwise specified**	**Every 4 cycles**	**End-of-Treatment (~ 30 days of the last dose of study treatment)**	
CT chest, abdomen, and pelvis[10]	May occur up to 8 days before cycles requiring images. Unscheduled assessments can occur at any time as needed if signs of disease progression are observed.		X[9]			X	X	X
Bone scans	May occur up to 8 days before cycles requiring images. Unscheduled assessments can occur at any time as needed if signs of disease progression are observed.		X[9]			X	X	X
Survival and SREs				X	X	X	X	X
Subsequent anticancer therapy				X	X	X	X	X
**Safety**								
Physical Examination	The screening physical examination will also include a full review of medical or oncological history and a complete examination of all organ systems. Limited symptom directed physical examinations and weight assessment will be conducted at other visits.		X	X	X		X	
Vital Signs	Body temperature, pulse/heart rate, respiratory rate, and BP are measured at the screening. Only BP is recorded at other visits.		X	X	X		X	
12-lead ECG	At screening, and as clinically indicated.		X					
Adverse Events		X[11]	Continuous from informed consent until 30 days after the last dose of study drugs					
Concomitant Medications			Continuous until 30 days after the last dose of study drugs					
ECOG-PS			X	X	X		X	

	Comments	Prescreening[1]	Screening	Treatment Phase (One cycle = 4 weeks [28 days])[2]				Posttreatment follow-up[3]
Activities and Forms to be Completed			≤35 Days Prior to Randomization	Cycle 1 Day 1	D1 C1-C6, D1 of every 2 cycles starting at C7 to C13, then D1 of every 4 cycles, unless otherwise specified	Every 4 cycles	End-of-Treatment (~ 30 days of the last dose of study treatment)	
**MRU**								
Medical Resource Utilization				Continuous				
**Population PK**								
PK sample[12]				C1D1 predose, 2h postdose	C2D1 predose, C3D1 predose and 2h postdose, C6D1 predose			

Abbreviations: ADT: androgen deprivation therapy; BP: blood pressure; C1D1: Cycle 1 Day 1; CT: computed tomography; DNA: ribonucleic acid; ECG: electrocardiogram; ECOG: Eastern Cooperative Oncology Group; HBsAg: hepatitis B virus surface antigen; HBV: hepatitis B virus; HCV: hepatitis C virus; MRI: magnetic resonance imaging; MRU: medical resource utilization; PSA: prostate-specific antigen; PK: pharmacokinetics; PS: performance status; QD: once daily; RNA: ribonucleic acid; SRE: skeletal-related event.

Footnotes:

1. Prescreening: For this optional Prescreening Phase, a separate written informed consent will be obtained from the participant (or participant's legal representative). Optional prescreening PSA evaluations will be performed by the local laboratories. This Prescreening Phase will allow additional time to obtain the required number of PSA values for determining eligibility till the last participant screening is successful. The participant must then sign another informed consent before any additional study-related procedures are.
2. During the Treatment Phase, study visits may occur within -3 ~ +2-day window.
3. Posttreatment Follow-up: Obtain survival status, collect information of development of symptomatic progression, initiation of any new systemic anticancer therapies until death, withdrawal of consent for study participation, or termination of the study (every 4 months ±7 days via clinic visit, telephone contact or an alternative contact method per institution policy/practice), whichever comes first. In addition, if participants discontinued study treatment prior to documented disease progression (distant metastases), CT of the chest, abdomen, and pelvis, or MRI, plus bone scan will be obtained and evaluated for disease progression in Posttreatment Follow-up visits (every 16 weeks ±7 days), until documentation of disease progression, then participants will be contacted every 4 months (±7 days).
4. Minimum criteria for the availability of documentation supporting the eligibility criteria are described in Source Documentation in Appendix 2: Regulatory, Ethical, and Study Oversight Considerations. Check clinical status again before first dose of study medication.
5. Apalutamide/Matched Placebo: Participants will receive oral daily apalutamide or matched placebo daily, starting on Cycle 1 Day 1. Participants in the placebo arm who are without evidence of distant metastasis (according to imaging result at Cycle 5 Day 1 as well as any other relevant unscheduled visit) will start receiving active therapy with apalutamide, at the time of having documented PSA progression or after completion of 5 cycles of placebo plus ADT treatment. One cycle consists of 28 days.
6. TSH testing will include total T3, free T4 (direct) and total T4 if TSH is not within normal range.
7. Maybe performed at screening to rule out active infection.
8. PSA evaluation for Cycle 3 Day 1, Cycle 4 Day 1, and Cycle 5 Day 1 should be collected no later than 1 week before each scheduled visit, eg, no late than Cycle 2 Day 21, Cycle 3 Day 21, and Cycle 4 Day 21 respectively. This will allow availability of laboratory results for determination of PSA progression status by each scheduled visit day (eg, Cycle 3 Day 1, Cycle 4 Day 1, and Cycle 5 Day 1) so that active treatment can be dispensed without delaying to the next scheduled visit if PSA progression has been confirmed for participants on Placebo arm.
9. Scans obtained prior to signing informed consent as part of the patient's standard of care may be allowed, provided the timing of the scans fall within the screening window.
10. Intravenous (IV) contrast is required when not medically contraindicated. Participants who have a contraindication to IV contrast may have MRI exams of the brain, abdomen, and pelvis performed in lieu of CTs and a non-contrast CT of the chest.
11. During this Prescreening Phase, the SAE reporting would be limited to SAEs related to the PSA blood draws.
12. PK Samples for population PK Analyses: The Day 1 Cycle 1 and Day 1 Cycle 3 samples will be collected at predose and 2h postdose. All samples on Day 1 of Cycles 2 and 6 will be collected prior to study drug administration. Date and time will be recorded for the doses administered 1 day preceding and during the PK sampling day. For all participants, PK samples will no longer be collected at the time of unblinding. PK sampling window: predose within 4.8 hours prior to dosing; 2h postdose (±10 minutes).

2. INTRODUCTION

Prostate cancer (PC) is the sixth most common cancer in men in China. There has been a marked increase in the number of PC cases diagnosed in recent years with an estimated new PC cases and deaths of 60,300 and 26,600, respectively in 2015.[1] Treatment aimed at eradicating the primary tumor, typically with surgery or radiation, is unsuccessful in ~30% of men, who develop recurrent disease that usually manifests first as a rise in plasma prostate-specific antigen (PSA).[11] Given that PC cells depend on the androgen receptor (AR) for their proliferation and survival, the standard treatment for patients with recurrent disease is androgen deprivation therapy (ADT) with a gonadotropin releasing hormone analog (GnRHa) with or without an anti-androgen.

The results are predictable, with a decline in PSA followed by tumor regression, a period of stability in which tumor burden and PSA remain stable, followed by rising PSA and regrowth as a castration-resistant prostate cancer (CRPC). When conventional imaging in CRPC shows no evidence of metastatic disease, it is termed non-metastatic castration-resistant prostate cancer (NM-CRPC).

Patients with NM-CRPC at high risk for metastases are characterized by rapidly rising PSA despite castration levels of testosterone (ie, a PSA doubling time [PSADT] of ≤10 months). Men with PC in this setting have a significant risk for the development of distant metastases and PC-specific death. Once the disease progresses to mCRPC, patients may develop symptoms of their disease and the prognosis is poor. Delaying of metastases for as long as possible is therefore the goal of therapy in NM-CRPC.

This is a post approval commitment (PAC) study being conducted to provide safety and efficacy data of apalutamide in Chinese participants with high-risk (defined as PSADT ≤10 months) NM-CRPC.

ERLEADA™ (apalutamide, also known as JNJ-56021927 and ARN-509) is an orally available, potent, new generation and selective AR antagonist that acts by inhibiting the action of androgen, nuclear translocation of the AR and DNA binding to androgen response elements. The safety and efficacy of apalutamide in the treatment of patients with NM-CRPC is supported by the data from Study ARN-509-003 (SPARTAN), a multicenter, randomized, double-blind, placebo-controlled study of apalutamide plus ADT or placebo plus ADT in 1207 men with NM-CRPC, designed to address this area of unmet medical need. Till now, ERLEADA has been approved in more than 75 countries including China since the first approval in the United States in February 2018 for the treatment of patients with NM-CRPC.[4,2]

For the most comprehensive nonclinical and clinical information regarding apalutamide, refer to the latest version of the Investigator's Brochure (IB) for apalutamide.[7]

The term "sponsor" used throughout this document refers to the entities listed in the Contact Information page(s), which will be provided as a separate document.

2.1. Study Rationale

This is a PAC study being conducted to provide safety and efficacy data of apalutamide as comparing to placebo in Chinese participants with high-risk (defined as PSADT ≤10 months) NM-CRPC.

2.2. Background

Nonclinical Studies

For details on safety pharmacology, toxicology, pharmacokinetics (PK), and metabolism profile in a preclinical setting, please refer to the IB.[7]

Clinical Studies

There are 2 ongoing (56021927PCR3003 [ATLAS] and 56021927PCR3011 [PROTEUS]) and 3 completed (ARN-509-003 [SPARTAN],[2] 56021927PCR3001 [ACIS] and 56021927PCR3002 [TITAN]), double-blind, placebo-controlled Phase 3 clinical studies.,.

Human Pharmacokinetics

Apalutamide was rapidly absorbed, and the median time to reach the maximum observed plasma concentration (t_{max}) was 2 hours (range: 1 to 5 hours under fasting conditions). The increases in plasma C_{max} values and in the area under the plasma concentration curve (AUC) were linear and dose proportional. Plasma apalutamide concentrations declined slowly, with a mean effective half-life value at steady-state of 3 days.

Based on population PK analysis for the ARN-509-003 (SPARTAN) study, there were no clinically relevant differences in exposure between White (Caucasian or Hispanic or Latino), Black (of African heritage or African American), Asian (non-Japanese), or Japanese participants.[4] More details please refer to IB.[7]

Efficacy/Safety Studies

Study ARN-509-003 (SPARTAN):

This multicenter, randomized, double-blind, placebo-controlled study was designed to demonstrate superiority in the metastasis-free survival (MFS) of men with high-risk NM-CRPC (ie, a PSADT of ≤10 months) treated with apalutamide versus [vs] placebo.[2] Upon blinded independent central review (BICR) confirmed development of distant metastatic disease, the sponsor offered ZYTIGA as an option for the first subsequent treatment after discontinuation of study drug, as long as the participant consented and resided in a country in which abiraterone acetate plus prednisone (or prednisolone) were indicated for the treatment of mCRPC before chemotherapy. Secondary efficacy parameters included time to metastasis, progression-free survival (PFS), time to symptomatic progression, overall survival (OS), and time to initiation of cytotoxic chemotherapy.

The primary data included 1,207 randomized participants (806 participants in the apalutamide arm and 401 participants in the placebo arm). Participants were stratified by PSADT (>6 months

vs ≤6 months, loco-regional disease (N0 or N1), and bone-sparing agent use (yes or no). As of the final analysis CCO (1 February 2020), the median survival follow-up time for all participants was 52 months.[7]

The clinical cutoff date for the primary analysis occurred on 19 May 2017. The independent data monitoring committee (IDMC) concluded that these improvements constituted compelling evidence of clinical benefit in the apalutamide group, and unanimously recommended unblinding the study and offering participants randomized to placebo group an option to cross over to receive apalutamide.

Treatment with apalutamide resulted in a statistically significant improvement in the primary endpoint, MFS, compared to placebo (Figure 1). Treatment with apalutamide significantly decreased the risk of distant metastasis or death by 72% compared with placebo (hazard ratio [HR]=0.280; 95% CI: 0.227, 0.346; p<0.0001). The median MFS was 40.5 months for the apalutamide arm and 16.2 months for the placebo arm.[4]

Figure 1: Kaplan-Meier Plot of Blinded Independent Central Review (BICR) Metastasis-Free Survival (MFS); Intent-to-treat Population (Study ARN-509-003 [SPARTAN])[4]

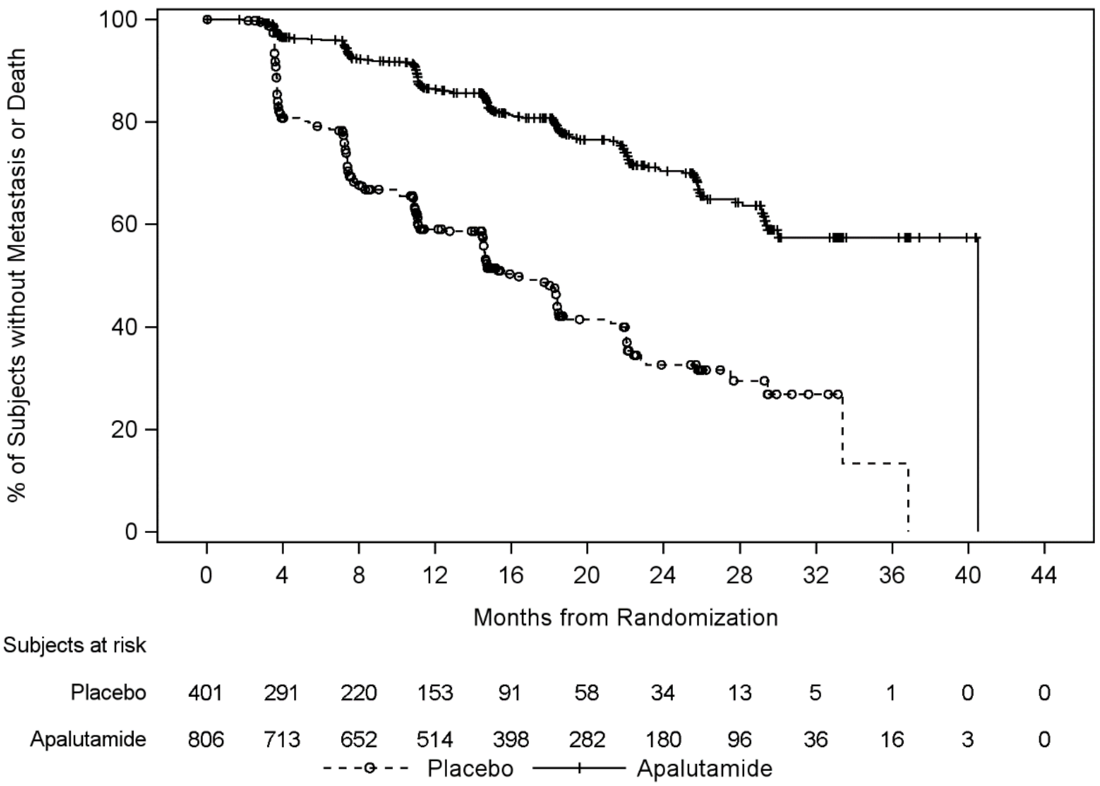

All secondary efficacy endpoints in Study ARN-509-003 (SPARTAN) favored treatment with apalutamide compared with placebo, based on HR analysis (Table 1). A confirmed PSA response was observed in 90% of participants in the apalutamide arm and 2.2% of participants in the placebo arm (p<0.0001); total response (confirmed and unconfirmed) was observed in 93% and 3.5% of apalutamide- and placebo-treated participants, respectively.[4]

Table 1: Summary of Secondary Efficacy Analysis (Study ARN-509-003 [SPARTAN])[4]

Endpoint [# of events]	Apalutamide (N=806) Median (month)	Placebo (N=401) Median (month)	HR (95% CI)	p-value*
TTM [366][a]	40.5	16.6	0.271 (0.219, 0.335)	< 0.0001
PFS [404] [a]	40.5	14.7	0.291 (0.238, 0.356)	< 0.0001
Time to symptomatic progression [264][b]	NE	NE	0.567 (0.443, 0.725)	< 0.0001
OS [428] [b]	73.9	59.9	0.784 (0.643, 0.956)	0.0161
Time to initiation of cytotoxic chemotherapy [258] [b]	NE	NE	0.629 (0.489, 0.808)	0.0002

CI: confidence interval; CCO: clinical cutoff date; PFS: progression-free survival; NE: not estimable; OS: overall survival; TTM: time to metastasis.
*p-value from stratified log-rank test.
a Source: Study ARN-509-003 (SPARTAN) Clinical Study Report (CCO: 19 May 2017): Tables 18 and 20.
b Source: Study ARN-509-003 (SPARTAN) Clinical Study Report addendum (CCO: 1 February 2020): Table 8, 10, and Attachment TEFPC 16.

Prostate-specific antigen progression was documented for 192 participants in the apalutamide arm (24%) and 334 participants in the placebo arm (83%). Treatment with apalutamide significantly decreased the risk of PSA progression by 94% compared with placebo (HR=0.064; 95% CI: 0.052, 0.080; p<0.0001). The median time to PSA progression was not estimable for the apalutamide arm and was 3.7 months in the placebo arm (Figure 2). The lower limit of the 95% CI for the median time to PSA progression for the apalutamide arm was 36.8 months.[4]

Figure 2: Kaplan-Meier Plot of Time to PSA Progression; Intent-to-treat Population (Study ARN-509-003 [SPARTAN])[4]

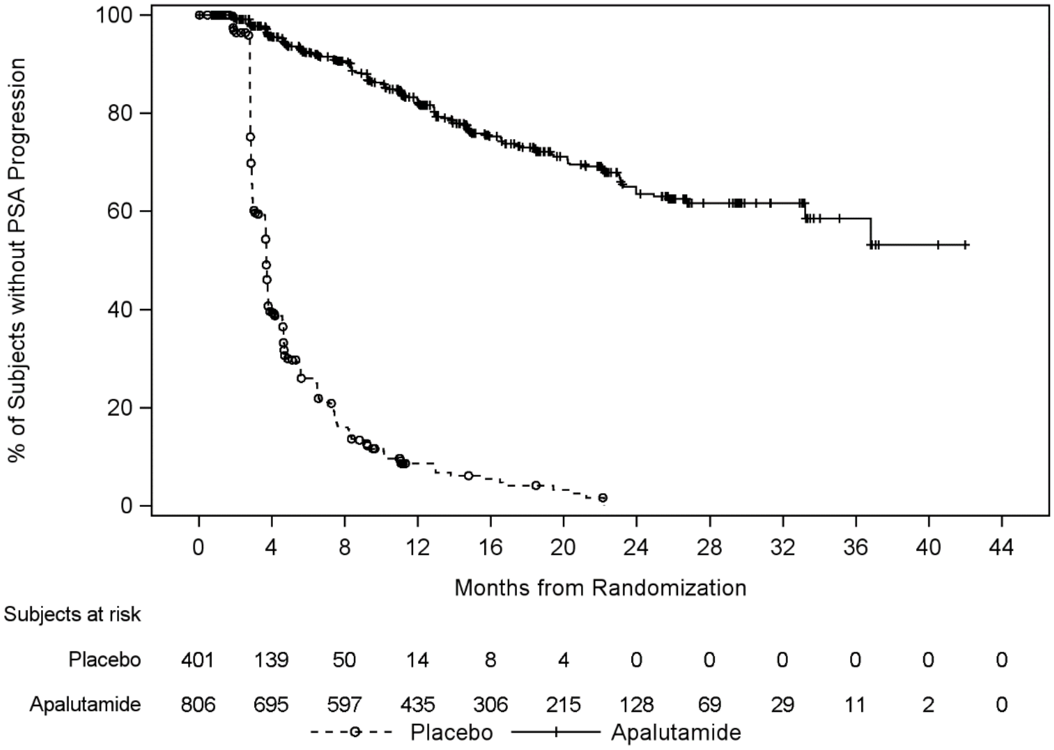

[GEFPC13.RTF] [JNJ-56021927\ARN-509-003\DBR_IA\RE_IA\PROD\GEFPC13.SAS] 03AUG2017, 19:57

The second progression-free survival during or after the first subsequent therapy (PFS2) is defined as the time from randomization to investigator-assessed disease progression (PSA, radiographic, symptomatic, or any combination) or death with the first subsequent therapy. The data for the PFS2 analysis were highly censored: 60% censored in the apalutamide arm and 53% censored in the placebo arm. The median PFS2 was longer for the apalutamide arm (55.6 months) compared with the placebo arm (41.2 months). This result represents a 44% reduction in the risk of disease progression with the next line of therapy or death in favor of the apalutamide arm (HR=0.565; 95% CI: 0.471, 0.677).[5]

Patient-reported outcome results indicated that there was no detriment to overall health-related quality of life with the addition of apalutamide to ADT in this population of generally asymptomatic NM-CRPC participants. Differences were observed between arms in FACT-P total and most subscale scores, generally showing less decline in HRQoL for participants in the apalutamide arm in later cycles, though these differences in group mean were all less than the threshold for clinically meaningful change in individual scores. Differences in least square mean changes from baseline which are greater than zero indicate less decline in the apalutamide arm compared with the placebo arm. Mean changes from baseline in EQ-5D-3L VAS score were calculated using mixed models for repeated measures. Differences were seen as scores decline for the placebo arm but remained stable for the apalutamide arm, with this difference exceeding a $p<0.05$ at Cycles 21 and 25, though the difference between arms remained relatively small with a

difference in group means well below the minimal important difference (MID) threshold for individual differences.[5]

The Phase 3 Study ARN-509-003 (SPARTAN) included safety data from 1201 participants with NM-CPRC who received at least 1 dose of either apalutamide or placebo (803 participants in the apalutamide arm and 398 participants in the placebo arm, and 76 participants who crossed over from placebo to apalutamide). As of the clinical cutoff date for the final analysis of 1 February 2020, 30% of participants in the apalutamide arm were continuing study treatment, no participants continued placebo treatment after study unblinding and being offered to cross over. Forty-six of 76 participants (61%) who crossed over continued to receive apalutamide as of the clinical cutoff date for this analysis. The median exposure in the apalutamide arm was nearly 3-times longer (32.9 months) than in the placebo arm (11.5 months).[2,4]

TEAEs leading to death were reported for 24 (3.0%) participants in the apalutamide arm and 2 (0.5%) participants in the placebo arm. Of the 24 participants in the apalutamide arm reported with TEAEs with an outcome of death, for 3 participants, progressive disease was their primary cause of death. All deaths except one were considered unrelated by the investigator: 1 participant in the apalutamide arm died after a TEAE that was considered drug-related by the investigator (acute myocardial infarction). The percentage of participants reported with TEAEs leading to treatment discontinuation was higher in the apalutamide arm (15%) compared with the placebo arm (7.3%), with TEAEs in the system organ class (SOC) of Skin and Subcutaneous Tissue Disorders resulting in the highest incidence of treatment discontinuations. Skin rash as the grouped term was the most commonly reported TEAE leading to treatment discontinuations and was the main reason for the difference in treatment discontinuations between the treatment arms. TEAEs leading to dose reduction were reported for 10% of participants in the apalutamide arm and 1.8% of participants in the placebo arm, TEAEs in the SOC of Skin and Subcutaneous Tissue Disorders and the SOC of General Disorders and Administration Site Conditions are the leading cause for dose reductions. Serious adverse reactions occurred in 36% of apalutamide arm -treated participants and 25% in participants receiving placebo. Frequently reported SAEs that occurred at a higher incidence in the apalutamide arm (>2%) than the placebo arm were fracture as a grouped term (5.4% apalutamide versus 1.0% placebo), ischemic heart disease as a grouped term (2.9% versus 1.8%), hematuria (2.6% versus 2.0%), urinary tract infection (2.1% versus 0.8%). Frequently reported SAEs that occurred at a higher incidence in the placebo arm than the apalutamide arm were urinary retention (1.6% apalutamide versus 4.8% placebo), hydronephrosis (1.6% versus 2.0%), and urinary tract obstruction (0.7% versus 1.0%); these events are frequent in the setting of disease progression.

The most frequently reported treatment-emergent adverse events (TEAEs) (ie, occurring in ≥15% of participants in either arm) were fatigue (33% apalutamide vs 21% placebo), hypertension (28% apalutamide vs 21% placebo), skin rash as the grouped term (26% apalutamide vs 6.3% placebo), diarrhea (23% apalutamide vs 15% placebo), nausea (20% apalutamide vs 16% placebo), fall (22% apalutamide vs 9.5% placebo), weight decreased (20% apalutamide vs 6.5% placebo), arthralgia (20% apalutamide vs 8.3% placebo), back pain (18% apalutamide versus 15% placebo), fracture as the grouped term (18% apalutamide versus 7.5% placebo), and hot flush (15% apalutamide

versus 8.5% placebo). When adjusted for exposure, skin rash, fall, weight decreased, arthralgia, and fracture were more frequent for the apalutamide arm.

Falls

In study ARN-509-003 (SPARTAN),[2] falls occurred in 22% of participants in the apalutamide arm and 9.5% of participants in the placebo arm. Among the 76 participants in the cross-over placebo-to-apalutamide arm, 11% of participants were reported with a TEAE of fall. Most falls were minor without resultant injury or only requiring noninvasive intervention, and very rarely were reason for treatment interruption or discontinuation.

Fracture

In study ARN-509-003 (SPARTAN),[2] most TEAEs of fracture were of Grade 1 or 2 severity, nonserious, and did not result in treatment modification or interruption. The preferred term rib fracture accounted for most events of fracture and was reported at a higher incidence in the apalutamide arm (7.5% versus 4.3% in the placebo arm). The use of bone-sparing agents at study entry did not appear to be protective.[4]

Seizure

In study ARN-509-003 (SPARTAN),[2]A total of 5 events of seizure were reported in the apalutamide arm and none in the placebo arm, nor in the cross-over placebo-to-apalutamide arm), all were of Grade 1 or 2 severity. Events of seizure were reported as serious TEAEs, and as mandated by the protocol, led to discontinuation of apalutamide therapy.

Skin Rash

In study ARN-509-003 (SPARTAN),[2] rash associated with apalutamide was most commonly described as macular or maculo-papular, with or without associated symptoms such as pruritus. Most TEAEs of skin rash were of Grade 1 or 2 severity. Grade 3 events of skin rash were reported for 5.2% of participants in the apalutamide arm. The exposure-adjusted incidence of skin rash in the apalutamide arm was lower at the final analysis compared with the first interim analysis, suggesting that skin rash is a relatively early event and that the risk for this event diminishes over time during apalutamide therapy. There were no reported Stevens-Johnson syndrome (SJS) or toxic epidermal necrolysis (TEN) cases. Skin rashes were manageable with dose interruption, dose reduction, topical and systemic therapy. No TEAE of skin rash led to death. As noted in the first interim analysis, the onset of skin rash typically occurred within the first 3 months of apalutamide treatment, with a median time of 82 days to the first occurrence of rash of any grade. Skin rash was a serious event for 2 participants in the apalutamide arm.

Ischemic Heart Disease

Events of ischemic heart disease were reported for participants in both arms of Study ARN-509-003 (5.5% in the apalutamide arm, 2.8% in the placebo arm), they were primarily for participants with a history of ischemic heart disease or risk factors. Based on similar risk factors and a higher frequency in the apalutamide arm, ischemic cerebrovascular disorder was also considered an ADR for apalutamide.

Grade 3 or higher events of ischemic heart disease were reported for 2.6% of participants in the apalutamide arm, 1.8% of participants in the placebo arm, and 2.6% in the placebo-apalutamide arm. Grade 5 events of ischemic heart disease were reported in 2 participants in the apalutamide arm and 1 participant in the placebo-apalutamide arm.

Ischemic Cerebrovascular Disorders

Based on the final analysis of Study ARN-509-003, ischemic cerebrovascular disorders (grouped term) was identified as a new AE of special interest and ADR.

Grade 3 events of ischemic cerebrovascular disorders were reported for 1.4% of participants in the apalutamide arm, Grade 4 events were reported for 1 participant (0.1%) in the apalutamide arm. Ischemic cerebrovascular disorders leading to dose reduction or interruption were reported for 0.6% of participants in the apalutamide arm, discontinuation of apalutamide therapy was reported for 0.9% of participants in the apalutamide arm.

2.2.1. Androgen Deprivation Therapy

All participants will be on a stable and continuous regimen of ADT (beginning on Cycle 1 Day 1). Androgen Deprivation Therapy with continuous GnRHa will be continued for all patients who have not been surgically castrated. GnRHa will be provided by the sponsor and should be used according to the approved prescribing information in China during the treatment phase.

2.3. Benefit/Risk Assessment

Based on the results from Study ARN-509-003 (SPARTAN), the addition of apalutamide to an ADT regimen resulted in statistically significant and clinically meaningful improvements in clinical outcomes compared with ADT alone. A significant improvement was demonstrated in favor of the apalutamide arm for the primary endpoint of MFS as well as for the key secondary endpoints of time to metastasis (TTM) and PFS. The safety results from this study demonstrated an acceptable safety profile for apalutamide plus ADT for the treatment of patients with NM-CRPC. Collectively, the results of Study ARN-509-003 (SPARTAN) demonstrate a favorable benefit/risk profile for apalutamide plus ADT for treatment of NM-CRPC in the target population of this PAC study.[2,4]

More detailed information about the known and expected benefits and risks of apalutamide may be found in the IB.

3. OBJECTIVES AND ENDPOINTS

OBJECTIVES

Primary Objective

The primary objective is to compare the improvement in time to PSA progression (TTPP, as defined by Prostate Cancer Working Group 2 [PCWG2]) of apalutamide vs placebo in Chinese participants with high-risk NM-CRPC.[a]

Secondary Objectives

The secondary objectives are:

- To evaluate the safety and tolerability of apalutamide
- To compare the PSA response rate in Chinese participants with high-risk NM-CRPC treated with apalutamide vs placebo
- To evaluate the PK of apalutamide and N-desmethyl apalutamide

Exploratory Objectives

The exploratory objectives are:

- To evaluate the MFS of Chinese participants with high-risk NM-CRPC treated with apalutamide
- To evaluate the OS of Chinese participants with high-risk NM-CRPC treated with apalutamide
- To evaluate the time to symptomatic progression in Chinese participants with high-risk NM-CRPC treated with apalutamide

ENDPOINTS

Primary Endpoint

- TTPP

Secondary Endpoints

- Incidence of AEs and laboratory abnormalities
- PSA response rate
- PK: Predose and 2 hours postdose plasma concentrations after single dose and at steady-state.

[a] Participants randomized to placebo arm will be crossed over to receive apalutamide treatment either after completion a maximum 5 cycles placebo treatment or at the time of having documented PSA progression, whichever comes earlier.

Exploratory Variables

- MFS
- OS
- Time to symptomatic progression:
  - Development of a skeletal-related event (SRE)
  - Pain progression or worsening of disease-related symptoms requiring initiation of a new systemic anticancer therapy.
  - Development of clinically significant symptoms due to loco-regional tumor progression requiring surgical intervention or radiation therapy.

Refer to Section 8, Study Assessments and Procedures for evaluations related to endpoints.

HYPOTHESIS

Participants receiving treatment with apalutamide in conjunction with ADT will have a longer TTPP compared with participants receiving placebo plus ADT.

4. STUDY DESIGN

4.1. Overall Design

This is a Phase IV, randomized, double-blind, placebo-controlled, multicenter study in evaluating the efficacy and safety of apalutamide vs placebo in male Chinese participants with high-risk NM-CRPC.

A target of 75 Chinese participants will be randomly assigned in a 2:1 ratio in this study to receive apalutamide or matching placebo. Participants will be stratified by PSADT (>6 months vs ≤6 months).

The study will consist of 4 phases:

- Prescreening Phase (optional)
- Screening Phase (≤35 days prior to randomization)
- Treatment Phase (starting on Day 1 of Cycle 1 until disease progression/ unacceptable toxicity, withdrawal of consent, death or termination of the study)
- Follow-up Phase (starting after end-of-treatment visit until death, withdrawal of consent for study participation, or termination of the study).

For an optional Prescreening Phase, a separate written informed consent will be obtained from the participant (or participant's legal representative). Optional prescreening PSA evaluations will be performed by the local laboratories. This Prescreening Phase will allow additional time to obtain the required number of PSA values for determining eligibility till the last participant screening is successful. The participant must then sign another informed consent before any additional study-related procedures are conducted.

Apalutamide or matched placebo will be administered orally on a continuous daily dosing schedule at a starting dose of 240 mg per day. Cycle 1 Day 1 must occur within 1 week of randomization. Androgen deprivation therapy with continuous GnRHa will be continued for all patients who have not been surgically castrated.

Each treatment cycle will consist of 28 days. Participants randomized to placebo, who do not have any evidence of distant metastasis (according to imaging result at Cycle 5 Day 1, as well as at any other relevant unscheduled visit) will be crossed over and start receiving active therapy of apalutamide after completion of 5 cycles of placebo plus ADT treatment. Participants who have PSA progression prior to completion of 5 cycles of study treatment, will cross over to apalutamide at the time of PSA progression. Any participant who develops distant metastatic disease will discontinue study treatment and may receive subsequent therapy according to standard of care. Around half of the participants in the placebo arm are expected to have documented PSA progression after completion of 5 cycles treatment, as estimated from the ARN-509-003 study data, and would therefore, allow sufficient time for the evaluation of TTPP and comparison between apalutamide and placebo.

During the treatment phase, PSA will be collected and analyzed by a central laboratory but investigators, participants, and the study team will be blinded to the results until completion of the study or the sponsor decides to unblind the study at final analysis. The central laboratory will analyze the individual PSA data, collected at baseline and post-baseline obtained within the first 5 cycles to flag out documented PSA progression per PCWG2 and the data will be entered into the interactive web response system (IWRS) to trigger the cross-over by an independent team not affiliated with the study team. The cross-over of control arm participants will be handled by IWRS to maintain the blinding of original treatment assignment of individual participant.

Participants will be followed for safety and efficacy as per Schedule of Activities (SoA) and will remain on study treatment until development of distant metastases as assessed by investigators or the development of unacceptable toxicity or the end of study defined as the time at which the MFS events are observed in approximately 60% of the study population or 3 years after the final analysis for the primary endpoint (TTPP), whichever comes first.

All participants will be monitored for safety during the Screening and Treatment Phases, and for up to 30 days after the last dose of study treatment. An End-of-Treatment (EoT) Visit will occur approximately 30 days of the last dose of study treatment. The Posttreatment Follow-up Phase will begin after the EoT visit. For participants entering Posttreatment Follow-up Phase with evidence of distant metastasis, they will be contacted every 4 months (±7 days) to obtain survival status, recording of development of symptomatic progression, initiation of any new systemic anticancer therapies until death, withdrawal of consent for study participation, or termination of the study. For participants entering Posttreatment Follow-up Phase without evidence of distant metastasis, Posttreatment Follow-up visits including disease assessment (computed tomography [CT] or magnetic resonance imaging [MRI], plus bone scans) will continue every 16 weeks (±7 days) until the documentation of disease progression, then participants will be contacted every 4 months (±7 days) to obtain survival status, recording of development of symptomatic

progression, initiation of any new systemic anticancer therapies until death, withdrawal of consent for study participation, or termination of the study.

An IDMC will be commissioned for this study to review the efficacy and safety results of final analysis. Refer to Committees Structure in Appendix 2: Regulatory, Ethical, and Study Oversight Considerations for details. The IDMC will consist of at least 1 medical oncologist or urologist or both and at least 1 statistician. Complete details regarding the IDMC's responsibilities, authorities, and procedures will be documented in the IDMC charter.

A diagram of the study design is provided in Section 1.2, Schema.

4.2. Scientific Rationale for Study Design

Short PSADT has been consistently associated with reduced time to first metastasis and death. Thus, the selected patient population represents 1 at a high risk for development of (distant) metastasis and PC-specific death.[6,10,9,2]

In China, there were no approved therapies at the initiation of this study for the treatment of patients with NM-CRPC. In clinical practice, continuous administration of ADT (with or without a first-generation anti-androgen) was the most often prescribed treatment thus constituted as part of the current *de facto* community standard practice. Therefore, randomization to either apalutamide or placebo was justified in this setting at the time of study initiation as the participants randomized to the control arm will be receiving the current standard of care in China. Since then, 2 other products have been conditionally approved or approved for the treatment of patients with NM-CRPC.

Randomization will be used to minimize bias in the assignment of participants to intervention groups, to increase the likelihood that known and unknown participant attributes (eg, demographic and baseline characteristics) are evenly balanced across intervention groups, and to enhance the validity of statistical comparisons across intervention groups. Blinded intervention will be used to reduce potential bias during data collection and evaluation of clinical endpoints.

Participants randomized to the placebo arm and without any evidence of distant metastasis will be allowed to early switch to receive apalutamide plus ADT at the time they have documented PSA progression or after completion of 5 cycles of placebo plus ADT treatment (refer to section 4.1). will minimize the exposure of participants randomized to the placebo arm to less effective treatment, as compelling evidence of efficacy and safety profile of apalutamide has been demonstrated in Study ARN-509-003 (SPARTAN).[4] Participants who develop distant metastatic disease prior to C6 will discontinue study treatment and may receive subsequent therapy as per standard of care for mCRPC.

Up to 5 cycles of placebo plus ADT treatment will allow an objective comparison of efficacy, measured by primary endpoint of TTPP, between treatment with apalutamide and placebo in Chinese high-risk NM-CRPC participants. Around half of the participants in the placebo arm are expected to have documented PSA progression by Cycle 5, as estimated from the ARN-509-003

(SPARTAN) study data, and would therefore, allow sufficient time for the evaluation of TTPP and comparison between apalutamide and placebo.[4]

Time to PSA progression is considered to be an appropriate primary endpoint in this PAC study as the clinical benefit observed in the primary and secondary endpoints of study ARN-509-003 (SPARTAN) was associated with significant improvements in TTPP. The proposed design using TTPP as primary endpoint will allow early read out and early switch of placebo participants to receive apalutamide, thereby improving participant well-being and study feasibility from operation perspective.[4]

4.2.1. Study-Specific Ethical Design Considerations

Potential participants will be fully informed of the risks and requirements of the study, and during the study, participants will be given any new information that may affect their decision to continue participation. They will be told that their consent to participate in the study is voluntary and may be withdrawn at any time with no reason given and without penalty or loss of benefits to which they would otherwise be entitled. Only participants who are fully able to understand the risks, benefits, and potential adverse events (AEs) of the study, and provide their consent voluntarily will be enrolled.

The primary ethical concern is participants randomized to the placebo arm will be exposed to less effective treatment (ADT alone). However, the study design allows for participants to cross over to the active arm as soon as PSA progression is determined, thus minimizing the exposure of participants to less effective treatment by allowing an early switch to apalutamide plus ADT using TTPP as the primary endpoint. Further, on the SPARTAN study about 6.2% of the placebo participants were found to have metastatic disease at the first post-baseline scan without prior PSA progression and only 1-2 participants in this study are expected to have the same situation given one third of 75 Chinese participants (about 25 participants) are to be randomly assigned to placebo arm. Therefore, the likelihood that a participant would develop metastatic disease before having the opportunity to cross over to apalutamide is small.[4]

4.3. Justification for Dose

The therapeutic dose was selected to be 240 mg/day given orally. The determination of the dose of 240 mg daily was based on an integrated approach, comparing the dose required for maximum tumor regression in mouse xenograft models,[10] clinical PK and pharmacodynamics (inhibition of 18-fluorodihydrotestosterone uptake and PSA response), and assessing the safety and tolerability at doses up to 480 mg.[3,8] For details pertaining to dose-modification, please refer Section 6.6.

4.4. End of Study Definition

The end of study is considered as the last scheduled study assessment shown in Schedule of Activities (SoA) for the last participant in the study. A participant will be considered to have completed the study if he has died before the end of the study. Participants lost to follow-up or withdrawn consent before the end of the study will not be considered as completing the study.

The final data from the study site will be sent to the sponsor (or designee) after completion of the final participant assessment at that study site, in the time frame specified in the Clinical Trial Agreement.

The end of trial will be defined as the time at which the MFS events are observed in approximately 60% of the study population or 3 years after the final analysis for the primary endpoint TTPP, whichever comes first.

5. STUDY POPULATION

Screening for eligible participants will be performed within ≤35 days before administration of the study intervention. Refer to Section 5.3, Screen Failures for conditions under which the repeat of any screening procedures are allowed.

The inclusion and exclusion criteria for enrolling participants in this study are described below. If there is a question about these criteria, the investigator must consult with the appropriate sponsor representative and resolve any issues before enrolling a participant in the study. Waivers are not allowed.

For a discussion of the statistical considerations of participant selection, refer to Section 9.1, Sample Size Determination.

5.1. Inclusion Criteria

Each potential participant must satisfy all of the following criteria to be enrolled in the study:

1. Male (according to their reproductive organs and functions assigned by chromosomal complement).

2. ≥18 years of age (or the legal age of consent in the jurisdiction in which the study is taking place).

3. Histologically or cytologically confirmed adenocarcinoma of the prostate without neuroendocrine differentiation or small cell features, with high risk for development of metastases, defined as PSADT ≤10 months. PSADT is calculated using at least 3 PSA values obtained during continuous ADT.

4. Castration-resistant PC demonstrated during continuous ADT, defined as 3 PSA rises at least 1 week apart, with the last PSA >2 ng/mL.

5. Surgically or medically castrated, with testosterone levels of <50 ng/dL. If the participant is medically castrated, continuous dosing with GnRHa must have been initiated at least 4 weeks prior to randomization and must be continued throughout the study to maintain castrate levels of testosterone.

6. Participants who received a first-generation anti-androgen (eg, bicalutamide, flutamide, nilutamide) must have at least a 4-week washout prior to randomization AND must

show continuing disease progression (an increase in PSA) after washout.

7. At least 4 weeks must have elapsed from the use of 5-α reductase inhibitors (eg, dutasteride, finasteride), estrogens (irrespective of dose used), and any other anticancer therapy prior to randomization, including chemotherapy given in the adjuvant/neoadjuvant setting (eg, clinical trial).

8. At least 4 weeks must have elapsed from major surgery or radiation therapy prior to randomization.

9. Eastern Cooperative Oncology Group (ECOG) - Performance Status (PS) grade 0 or 1 (Appendix 5: ECOG-PS Score).

10. Resolution of all acute toxic effects of prior therapy or surgical procedure to Grade 1 or baseline prior to randomization.

11. Adequate organ function as defined by the following criteria:

    – Serum aspartate aminotransferase (AST; serum glutamic oxaloacetic transaminase [SGOT]) and serum alanine aminotransferase (ALT; serum glutamic pyruvic transaminase [SGPT]) ≤2.5x upper limit of normal (ULN).
    – Total bilirubin ≤1.5 x ULN; participants with Gilbert syndrome can be enrolled if conjugated bilirubin is within normal limits (≤1.5 x ULN or direct bilirubin ≤ULN for participants with total bilirubin levels >1.5 x ULN).
    – Serum creatinine ≤2 x ULN.
    – Absolute neutrophil count (ANC) ≥1500/μL.
    – Platelets ≥100,000/μL.
    – Hemoglobin ≥9.0 g/dL.
    – Administration of growth factors or blood transfusions will not be allowed within 4 weeks of the hematology labs required to confirm eligibility.

12. Must sign an informed consent form (ICF; or their legally acceptable representative must sign) indicating that he understands the purpose of, and procedures required for, the study and is willing to participate in the study.

13. Willingness and ability to comply with scheduled visits, treatment plans, laboratory and radiographic assessments, and other study procedures, including ability to swallow study drug tablets, and long-term survival follow-up visits.

14. Willing and able to adhere to the lifestyle restrictions specified in this protocol.

5.2. Exclusion Criteria

Any potential participant who meets any of the following criteria will be excluded from participating in the study:

1. Presence of distant metastases, including central nervous system (CNS) and vertebral or meningeal involvement, or history of distant metastases. Exception: Pelvic lymph nodes <2 cm in short axis (N1) located below the iliac bifurcation are allowed.

2. Symptomatic loco-regional disease requiring medical intervention, such as moderate or severe urinary obstruction or hydronephrosis, due to primary tumor (eg, tumor obstruction of bladder trigone).

3. Prior treatment with second generation anti-androgens (eg, enzalutamide).

4. Prior treatment with CYP17 inhibitors (eg, abiraterone acetate, orteronel, galerterone, ketoconazole, aminoglutethimide) for PC.

5. Prior treatment with radiopharmaceutical agents (eg, Strontium-89), immunotherapy (eg, sipuleucel-T), or any other investigational agent for NM-CRPC.

6. Prior chemotherapy for PC, except if administered in the adjuvant/neoadjuvant setting.

7. History of seizure or condition that may pre-dispose to seizure (eg, prior stroke within 1 year prior to randomization, brain arteriovenous malformation, Schwannoma, meningioma, or other benign CNS or meningeal disease which may require treatment with surgery or radiation therapy).

8. Concurrent therapy with any of the following (all must have been discontinued or substituted for at least 4 weeks prior to randomization):

   – Medications known to lower the seizure threshold.
   – Herbal and non-herbal products that may decrease PSA levels.
   – Spironolactone.
   – Systemic (oral/IV/IM) corticosteroids. Short term use (≤4 weeks) of corticosteroids during the study is allowed if clinically indicated, but it will be tapered off as soon as possible.
   – Any other experimental treatment on another clinical trial.
   – Agents indicated for the prevention of SREs in participants with solid tumors (eg, denosumab [XGEVA®]), zoledronic acid [ZOMETA®]). Treatment with bone-sparing agents for the treatment of osteoporosis (eg, denosumab [PROLIA®], zoledronic acid [ACLASTA®]) is not exclusionary.

   Refer to Section 6.5, Concomitant Therapy for details regarding prohibited and

restricted therapy during the study.

9. History of severe/unstable angina, myocardial infarction, symptomatic congestive heart failure, arterial or venous thromboembolic events (eg, pulmonary embolism, cerebrovascular accident including transient ischemic attacks) or clinically significant ventricular arrhythmias within 6 months prior to randomization.

10. History of malignancy within 5 years before screening (exceptions are squamous and basal cell carcinomas of the skin, superficial bladder cancer.

11. Uncontrolled hypertension (systolic blood pressure (BP) ≥160 mm Hg or diastolic BP ≥100 mm Hg). Participants with a history of uncontrolled hypertension are allowed provided BP is controlled by anti-hypertensive treatment.

12. Gastrointestinal disorder affecting absorption.

13. Active infection, such as human immunodeficiency virus, or active hepatitis.

14. Received an investigational intervention (including investigational vaccines) or used an invasive investigational medical device within 4 weeks before the planned first dose of study intervention or is currently enrolled in an investigational study.

15. Men who are sexually active with women of childbearing potential except:

    - agree to use a condom with spermicidal foam/gel/film/cream/suppository
    - agree to not donate sperm during the study and for at least 3 months after the last dose of study drug
    - do not plan to father a child during the study or within 3 months after the last dose of study drug

16. Any condition for which, in the opinion of the investigator, participation would not be in the best interest of the participant (eg, compromise the well-being) or that could prevent, limit, or confound the protocol-specified assessments.

17. Employee of the investigator or study site, with direct involvement in the proposed study or other studies under the direction of that investigator or study site, as well as family members of the employees or the investigator.

18. Known allergies, hypersensitivity, or intolerance to apalutamide or its excipients (refer to IB).

NOTE: Investigators should ensure that all study enrollment criteria have been met at screening. If a participant's clinical status changes (including any available laboratory results or receipt of additional medical records) after screening but before the first dose of study intervention is given such that he or she no longer meets all eligibility criteria, then the participant should be excluded from participation in the study. Section 5.3, Screen Failures, describes options for retesting. The

required source documentation to support meeting the enrollment criteria are noted in Appendix 2: Regulatory, Ethical, and Study Oversight Considerations.

5.3. Screen Failures

Participant Identification, Enrollment, and Screening Logs

The investigator agrees to complete a participant identification and enrollment log to permit easy identification of each participant during and after the study. This document will be reviewed by the sponsor study-site contact for completeness.

The participant identification and enrollment log will be treated as confidential and will be filed by the investigator in the study file. To ensure participant confidentiality, no copy will be made. All reports and communications relating to the study will identify participants by participant identification and age at initial informed consent. In cases where the participant is not randomized into the study, the date seen and age at initial informed consent will be used.

Individuals who do not meet the criteria for participation in this study (screen failure) may be rescreened once. Rescreening is at the discretion of the investigator. Participants who are to be rescreened must sign a new ICF before rescreening. Rescreening and subsequent randomization activities must be conducted in accordance with all protocol-defined windows and timelines. Rescreened participants should be assigned a new participant number.

6. STUDY INTERVENTION

6.1. Study Interventions Administered

Treatment will begin on Day 1 of Cycle 1 and each treatment cycle will consist of 28 days. Participants will be randomly assigned in a 2:1 ratio to receive either apalutamide or matching placebo, as below (refer to Table 2). ADT (surgical castration or GnRHa) will be continued in all participants.

- Investigational group will receive apalutamide 240 mg (4×60 mg tablets) daily.
- Control group will receive placebo daily from Cycle 1 Day 1. Participants who do not have distant metastasis will switch to treatment with apalutamide after completion of 5 cycles of placebo treatment (handling by the IWRS). Participants who have PSA progression prior to completion of 5 cycles of study treatment, will cross over to apalutamide at the time of PSA progression.

Study drug intake will occur on an outpatient basis on non-PK sampling days. On PK sampling days, which include Cycle 1 Day 1, Cycle 2 Day 1, Cycle 3 Day 1, and Cycle 6 Day 1, intake of apalutamide must occur at the study site under supervision of site personnel after the predose PK samples are collected. On PK sampling days, participants will be fasted at least 2 hours prior to dosing, and will continue fasting (except for approved snack) until completion of the 2-hour post-dose assessments on Cycle 1 Day 1 and Cycle 3 Day 1. On non-PK sampling days, apalutamide may be taken with or without food. A modified dosing schedule may be required on

PK sampling days. Study-site personnel will instruct participants on how to store study drug for at-home use as indicated for this protocol.

Table 2: Description of Interventions

Arm Name	Arm A	Arm B
**Intervention Name**	Apalutamide	Placebo
**Type**	A drug	A matching placebo to active drug
**Dose Formulation**	Immediate release tablet	Immediate release tablet
**Unit Dose Strength(s)**	4×60 mg	4×60 mg
**Dosage Level(s)**	240 mg daily	240 mg daily
**Route of Administration**	Oral	Oral
**Use**	Experimental	Placebo-Comparator
**Packaging and Labeling**	120-ct, 160°cc HDPE bottles with CRC and include desiccant.	120-ct, 160°cc HDPE bottles with CRC and include desiccant.
**Child-resistant packaging**	Yes	Yes
**Delivery instructions**	Apalutamide: QD without regard to food	Apalutamide: QD without regard to food
**Names/Aliases**	JNJ-56021927	NA

ADT: androgen deprivation therapy, being either surgical castration or gonadotrophin-releasing hormone agonists (GnRHa), will be continued as background therapy for all participants. ADT with GnRHa will be continued for all patients who have not been surgically castrated. CRC: child-resistant closures; HDPE: high density polyethylene; QD: once daily.

Study intervention administration must be captured in the source documents and the electronic case report form (eCRF). Study-site personnel will instruct participants on how to store study intervention for at-home use as indicated for this protocol.

Apalutamide will be manufactured and provided under the responsibility of the sponsor. Refer to the IB for a list of excipients.

For a definition of study intervention overdose, refer to Section 8.4, Treatment of Overdose.

6.2. Preparation/Handling/Storage/Accountability

All study intervention must be stored at controlled temperatures ranging from 15°C to 30°C.

Refer to the pharmacy manual/study-site investigational product and procedures manual for additional guidance on study intervention preparation, handling, and storage.

The dispensing of study intervention to the participant, and the return of study intervention from the participant (if applicable), must be documented on the intervention accountability form. Participants or their legally acceptable representatives where applicable, must be instructed to return all original containers, whether empty or containing study intervention. All study intervention will be stored and disposed of according to the sponsor's instructions. Study-site personnel must not combine contents of the study intervention containers.

The participants must return unused study intervention to the study site.

Study intervention must be handled in strict accordance with the protocol and the container label,

and must be stored at the study site in a limited-access area or in a locked cabinet under appropriate environmental conditions. Unused study intervention, and study intervention returned by the participant, must be available for verification by the sponsor's study-site monitor during on-site monitoring visits. The return to the sponsor of unused study intervention, or used returned study intervention for destruction, will be documented on the intervention return form. When the study site is an authorized destruction unit and study intervention supplies are destroyed on-site, this must also be documented on the intervention return form.

Potentially hazardous materials such as used ampules, needles, syringes and vials containing hazardous liquids, should be disposed of immediately in a safe manner and therefore will not be retained for intervention accountability purposes.

Study intervention should be dispensed under the supervision of the investigator or a qualified member of the study-site personnel, or by a hospital/clinic pharmacist. Study intervention will be supplied only to participants participating in the study. Returned study intervention must not be dispensed again, even to the same participant. Whenever a participant brings his study intervention to the study site for pill count, this is not seen as a return of supplies. Study intervention may not be relabeled or reassigned for use by other participants. The investigator agrees neither to dispense the study intervention from, nor store it at, any site other than the study sites agreed upon with the sponsor.

6.3. Measures to Minimize Bias: Randomization and Blinding

Intervention Allocation

Procedures for Randomization

Central randomization will be implemented in this study. Participants will be randomly assigned to 1 of 2 intervention groups no more than 1 week prior to dosing and will be based on a computer -generated randomization schedule prepared before the study under the supervision of the sponsor. The randomization will be balanced by using randomly permuted blocks and will be stratified by PSADT (>6 months vs ≤6 months). The IWRS will assign a unique intervention code, which will dictate the intervention assignment and matching study intervention kit for the participant. The requestor must use his own user identification and personal identification number when contacting the IWRS, and will then give the relevant participant details to uniquely identify the participant.

To ensure an accurate and consistent determination of PSADT across all sites, the IWRS will also provide PSADT calculations (using a linear regression model of the normal logarithm of PSA and time) based on at least 3 PSA values obtained during continuous ADT. All available consecutive PSA values obtained within 24 months prior to randomization beginning with the earliest value chosen for the PSADT calculation must be entered in the IWRS. The first and last PSA values used in the calculation must be separated by at least 8 weeks.

Those same PSA values will be used during Screening to determine whether the patient is eligible for the study (inclusion criterion 3). To pre-screen participants for possible enrollment

into the study, PSADT can be calculated using the Memorial Sloan-Kettering Cancer Center (MSKCC) PSADT prediction tool, available at the following website:

http://nomograms.mskcc.org/Prostate/PsaDoublingTime.aspx

Blinding

The investigator will not be provided with randomization codes. The codes will be maintained within the IWRS, which has the functionality to allow the investigator to break the blind for an individual participant.

Prostate-specific antigen will be collected and analyzed by a central laboratory but investigators, participants, and the study team will be blinded to the results until the full unblinding of the study. Control arm participants without distant metastasis will switch to treatment with apalutamide plus ADT at the time PSA progression (per PCWG2) is documented or participants have completed 5 cycles treatment of placebo plus ADT. The central laboratory will analyze the individual PSA data, collected at baseline and post-baseline obtained within the first 5 cycles to flag out documented PSA progression per PCWG2 and the data will be entered into the IWRS to trigger the cross-over by an independent team not affiliated with the study team. The cross-over of control arm participants will be handled by IWRS to maintain the blinding of original treatment assignment of individual participant.

Under normal circumstances, the blind should not be broken until completion of the study or the sponsor decides to unblind the study at final analysis. Otherwise, the blind should only be broken if, in a specific emergency, the treatment or course of action would be dictated by knowing the treatment status of the participant. It is recommended that the investigator contact the sponsor or its designee if possible to discuss the particular situation before breaking the blind. Telephone contact with the sponsor or its designee will be available 24 hours per day, 7 days per week. In the event that the blind is broken, the sponsor must be informed as soon as possible. The date, time, and reason for the unblinding must be documented in the IWRS, in the appropriate section of the eCRF, and in the source document. The documentation received from the IWRS indicating the code break must be retained with the participant’s source documents in a secure manner.

Randomization codes will be disclosed fully only if the study is completed and the clinical database is closed or the sponsor decides to unblind the study at final analysis.

6.4. Study Intervention Compliance

At each clinic visit, participants will be asked to return any remaining study drug from the previous dosing cycle as well as all used and unused study drug containers.

Treatment compliance will be defined as the number of tablets taken divided by the expected number of tablets and reported as percentage. In case of dose reductions, the expected number of tablets will reflect the new dose level. Tablets that are not returned will be considered to have been taken, unless otherwise specified in the eCRF.

The investigator or designated study site personnel will be responsible for providing additional instruction to any participant who is not compliant with taking the study drug. In the absence of toxicity, if the dosing compliance is not 100%, then investigators or designated study site personnel should re-instruct participants regarding proper dosing procedures and the participant may continue study treatment.

The study site must maintain accurate records demonstrating dates and amount of study drug received, to whom dispensed (participant-by-participant accounting), and accounts of any study drug accidentally or deliberately destroyed. At the end of the study, reconciliation must be made between the amount of study drug supplied, dispensed, and subsequently destroyed or returned to sponsor or its representative.

6.5. Concomitant Therapy

Prestudy therapies administered up to 35 days before first dose of study intervention must be recorded at screening.

Concomitant therapies must be recorded throughout the study beginning with start of the first dose of study intervention to 30 days after the last dose of study intervention.

All therapies (prescription or over the counter medications, including vaccines, vitamins, herbal supplements; non-pharmacologic therapies such as electrical stimulation, acupuncture, special diets, exercise regimens) different from the study intervention must be recorded in the eCRF. Recorded information will include a description of the type of therapy, duration of use, dosing regimen, route of administration, and indication. Modification of an effective preexisting therapy should not be made for the explicit purpose of entering a participant into the study.

Continuous treatment with a GnRHa or surgical castration is mandatory. The choice of GnRHa is at the discretion of the investigator. Dose and dose schedule (without interruption) will be consistent with the prescribing information and will only be adjusted if clinically indicated to maintain castrate concentrations of testosterone.

Salvage radiation for loco-regional pelvic disease and surgical procedures (eg, transurethral resection of the prostate [TURP], urethral and ureteral stent placement) to treat localized progression or symptoms are allowed. Participants receiving these therapies may continue on study drug.

6.5.1. Suggested Therapy

Fracture, fall and hypothyroidism are known risks associated with apalutamide. Participants will be evaluated for fracture and fall risk. Monitor and manage participants at risk for fractures according to established treatment guidelines and consider use of bone targeted agents. Participants are strongly encouraged to obtain an adequate intake of dietary calcium (at least 1,000 mg per day, including supplements if necessary) and vitamin D [at least 800-1,000 international units (or according to the product label-prescribing information in the country of residence) per day for adults 50 years of age and older] and to engage in regular exercise to maintain muscle strength and bone density.

Thyroid replacement therapy, when clinically indicated, will be initiated or dose-adjusted.

6.5.2. Prohibited Therapy

Concurrent enrollment in another investigational drug or device study is prohibited during the Treatment Phase.

As a class effect, AR antagonists have been associated with seizures due to an off-target mechanism of action (gamma amino butyric acid chloride channel inhibition). Drugs known to lower the seizure threshold or cause seizures are prohibited and a representative list is included below:

- Atypical antipsychotics (eg, clozapine, olanzapine, risperidone, ziprasidone)
- Bupropion
- Lithium
- Meperidine (pethidine)
- Phenothiazine antipsychotics (eg, chlorpromazine, mesoridazine, thioridazine)
- Tricyclic antidepressants (eg, amitriptyline, desipramine, doxepin, imipramine, maprotiline, mirtazapine)
- Aminophylline/theophylline.

The following medications are prohibited while on study until the End-of-Treatment Visit or 30 days after last dose of study drug. The sponsor must be notified in advance (or as soon as possible thereafter) of any instances in which prohibited therapies are administered. Chemotherapeutic, biologic, or other agents with anti-tumor effect against PC.

- Anti-androgens (except as prescribed in the protocol)
- 5-α reductase inhibitors
- Estrogens
- Progestational agents (eg, cyproterone acetate)
- Androgens
- Oral ketoconazole
- Spironolactone
- Bone targeted agents indicated for the treatment of metastatic PC (bisphosphonates or denosumab) are prohibited (NOTE: bone targeted agents indicated for osteoporosis are allowed).

The sponsor must be notified in advance (or as soon as possible thereafter) of any instances in which prohibited therapies are administered.

6.5.3. Restricted Concomitant Therapy

Highlights of drug interaction with apalutamide are summarized below. Refer to Sections 4.3.4 and 5.10 of the IB and associated addenda for complete details on the drug interaction potential of apalutamide, which include examples of medication that 1) may influence the effect of apalutamide, and 2) their effects may be influenced by apalutamide.

- Medications that inhibit CYP2C8 or CYP3A4: Co-administration of a strong CYP2C8 or CYP3A4 inhibitor is predicted to increase the steady-state exposure of the active moieties (sum of unbound apalutamide plus the potency-adjusted unbound N-desmethyl apalutamide). No initial dose adjustment is necessary however, consider reducing the apalutamide dose based on individual tolerability. Mild or moderate inhibitors of CYP2C8 or CYP3A4 are not expected to affect the exposure of apalutamide.
- Effect of apalutamide on drug metabolizing enzymes: Apalutamide is a strong inducer of CYP3A4 and CYP2C19, and a weak inducer of CYP2C9 in humans. Concomitant use of apalutamide with medications that are primarily metabolized by CYP3A4, CYP2C19, or CYP2C9 can result in lower exposure to these medications. Substitution for these medications is recommended when possible or evaluate for loss of efficacy if medication is continued. Concomitant administration of apalutamide with medications that are substrates of UGT can result in decreased exposure. Use caution if substrates of UGT must be co-administered with apalutamide and evaluate for loss of efficacy.
- Effect of apalutamide on drug transporters: Apalutamide was clinically shown to be a weak inducer of P-gp, BCRP, and OATP1B1. Concomitant use of apalutamide with medications that are substrates of P-gp, BCRP, or OATP1B1 can result in lower exposure of these medications. Use caution if substrates of P-gp, BCRP or OATP1B1 must be co-administered with apalutamide and evaluate for loss of efficacy if medication is continued.
- Corticosteroids (Oral, IV, or IM): due to possible resistance mechanisms, which may be contributed by glucocorticoid receptor signaling, concurrent use of corticosteroids during the study is not recommended; short term use (≤4 weeks) will be allowed if clinically indicated, however, its use must be tapered off as soon as possible.

The sponsor must be notified in advance (or as soon as possible thereafter) of any instances in which prohibited therapies are administered.

6.6. Dose Modification

Any dose/dosage adjustment should be overseen by medically-qualified study-site personnel (principal or sub-investigator unless an immediate safety risk appears to be present).

General principles on dose modifications (except for rash, see Section 6.6.1 for details):

- Grade 1 or Grade 2 toxicities should be managed symptomatically without dose adjustments. Appropriate medical treatment should be used.
- In the event of a Grade 3 or higher toxicity, study drug should be held as detailed in Table 3 and Table 4.

- For the first occurrence of a Grade 3 or higher toxicity, dose reductions are allowed at investigator's clinical judgment; if the Grade 3 or higher event recurs, dose reduction is mandatory.
- If Grade 3 or higher toxicity does not resolve to Grade 1 or baseline within 2 cycles, the participant should be discontinued from treatment or the investigator's rationale to continue treatment must be discussed with the sponsor.
- The investigator's rationale to re-escalate treatment must be discussed with and approved by the sponsor's medical monitor on an individual basis prior to implementation.
- Instructions for dose modifications are provided as guidance and should not replace the investigator's own clinical judgment.

Table 3 summarizes apalutamide/placebo dose modifications for drug-related toxicities (except rash). Dose modifications for drug-related rash are summarized in Table 4.

Table 3: Dose Modifications of Apalutamide/Placebo (except for rash, if rash occurs, see Table 4)

**Severity**	**Number of apalutamide/placebo tablets**
Grade 1 or 2	No change or hold until return to baseline
≥Grade 3	Hold until Grade 1 or baseline, resume at full dose; dose reductions are allowed at investigator's clinical judgment
Recurrence ≥Grade 3	Hold until Grade 1 or baseline; reduce 1 dose level; 2 dose reductions are allowed for recurrent treatment-related toxicity (180 mg [3 tablets]) and 120 mg [2 tablets]). Discontinue if toxicity persists after 2 dose reductions.
First occurrence of seizure of any grade	Discontinue
**Note: Adverse events are graded according to NCI-CTCAE Version 4.03**	

Abbreviation Key: NCI-CTCAE: National Cancer Institute-Common Terminology Criteria for Adverse Events.

6.6.1. Dose Modifications for Rash and Rash Management

Dose modifications for rash are allowed and are summarized in Table 4. If the skin rash has any component of desquamation, mucosal involvement, or pustules, stop dosing with study treatment, refer to a dermatologist for evaluation, and a skin biopsy is recommended (in addition to the interventions listed in Table 4) (and complete the skin rash eCRF). If the skin rash is Grade 3 or higher, asking the participant to consent to documentation by a photograph and further evaluation by a dermatologist will also be considered (and complete the skin rash eCRF). Completion of the skin rash eCRF will also be required if the skin rash leads to permanent discontinuation of study drug. If the study drug needs to be held for more than 14 days, the investigator will discuss with the sponsor's medical monitor. Apalutamide/placebo can be held up to 28 days in the event of rash. If treatment interruption for any AE grade is longer than 28 days, resumption of treatment may still be feasible. Please reach out to the sponsor to discuss further management. Dose re-escalation for rash is allowed for apalutamide/placebo at the discretion of the investigator.

Table 4: Dose-modification and Management of Drug-related Rash

Severity	Intervention
**Grade 1**	• Continue apalutamide/placebo at current dose<br>• Initiate dermatological treatment[a]<br>o Topical steroid cream AND<br>o Oral Antihistamines<br>• Monitor for change in severity[a]
**Grade 2 (or symptomatic Grade 1)[b]**	• Hold apalutamide/placebo for up to 28 days<br>• Initiate dermatological treatment[a]<br>o Topical steroid cream AND<br>o Oral Antihistamines<br>• Monitor for change in severity[a]<br>o If rash or related symptoms improve, reinitiate apalutamide/placebo when rash is Grade ≤1. Consider dose reduction at half the dose
**Grade ≥3[d]**	• Hold apalutamide/placebo for up to 28 days<br>• Initiate dermatological treatment[a]<br>o Topical steroid cream AND<br>o Oral Antihistamines AND<br>o Consider short course of oral steroids[c]<br>• Reassess after 2 weeks (by site staff), and if the rash is the same or has worsened, initiate oral steroids (if not already done) and refer the participant to a dermatologist<br>o Reinitiate apalutamide/placebo at half the dose[a] when rash is Grade ≤1.<br>• If after 28 days, rash has not resolved to Grade ≤1, contact the sponsor to discuss further management and possible discontinuation of study drug

Note: Rash may be graded differently according to the type of rash and associated symptoms. For example, maculo-papular rash is graded by body surface area covered and not severity of the rash. Please consult NCI-CTCAE Version 4.03 for specific grading criteria for other types of rash.

a Obtain bacterial/viral cultures if infection is suspected

b Participant presents with other rash related symptoms such as pruritus, stinging, or burning

c If a participant previously started oral corticosteroids, continue for at least 1 week after resumption of reduced dose of apalutamide/placebo. If the proposed total oral steroid use will exceed 28 days, contact the sponsor.

d If there is blistering or mucosal involvement, stop apalutamide/placebo dosing immediately and contact the sponsor

Abbreviations: NCI-CTCAE: National Cancer Institute-Common Terminology Criteria for Adverse Events.

6.7. Long-term Follow-up

Investigators will recontact the participant to obtain posttreatment follow-up information regarding the participant's safety or survival status as noted in the informed consent form (refer to Informed Consent in Appendix 2: Regulatory, Ethical, and Study Oversight Considerations).

Participants will be instructed that study intervention will not be made available to them after they have completed/discontinued study intervention and that they should return to their primary physician to determine standard of care.

7. DISCONTINUATION OF STUDY INTERVENTION AND PARTICIPANT DISCONTINUATION/WITHDRAWAL

7.1. Discontinuation of Study Intervention

Discontinuing treatment will not result in an automatic withdrawal from the study. Participants who discontinue treatment, but do not withdraw consent, should complete the EoT Visit within 30 days of the last dose of study drug and continue the scheduled assessments according to the Posttreatment Follow-up Phase in the Schedule of Activities (SoA).

A participant's study intervention must be discontinued if:

- Documented distant metastases
- The investigator believes that for safety reasons or tolerability reasons (eg, AE) it is in the best interest of the participant to discontinue study intervention
- Initiation of any subsequent prostate anticancer therapy or procedure not specified in the protocol during treatment phases
- Seizure of any grade or Grade 4 neurotoxicity (see Section 6.6);
- Other study drug toxicity (see Section 6.6)
- Withdrawal of consent for continued treatment.

All participants discontinuing study treatment will enter the Posttreatment Follow-up Phase. For participants entering Posttreatment Follow-up Phase with evidence of distant metastasis, they will be followed for survival status, the development of symptomatic progression, initiation of any new systemic anticancer therapies until death, withdrawal of consent for study participation, or termination of the study (every 4 months ±7 days via clinic visit, telephone contact or an alternative contact method per institution policy/practice), whichever comes first. In addition, if participants discontinued study treatment prior to documented disease progression (distant metastases), CT of the chest, abdomen, and pelvis, or MRI, plus bone scan will be obtained and evaluated for disease progression in Posttreatment Follow-up visits (every 16 weeks ±7 days), until documentation of disease progression, then participants will be contacted every 4 months (±7 days) to obtain survival status, recording of development of symptomatic progression, initiation of any new systemic anticancer therapies until death, withdrawal of consent for study participation, or termination of the study.

When a participant withdraws before completing the study, the reason for withdrawal is to be documented in the eCRF and in the source document. Study drug assigned to the withdrawn participant may not be assigned to another participant. Participants who withdraw will not be replaced.

Participants will be followed for at least 30 days after the last dose of study drug. If a participant is withdrawn from treatment due to an AE, please refer to Appendix 3: Adverse Events: Definitions and Procedures for Recording, Evaluating, Follow-up, and Reporting.

7.2. Participant Discontinuation/Withdrawal From the Study

A participant will be withdrawn from the study for any of the following reasons:

- Loss of follow-up
- Withdrawal of consent for subsequent data collection
- Study is terminated by the sponsor

When a participant withdraws from the study, the reason for withdrawal is to be documented in the eCRF and in the source document. Study intervention assigned to the withdrawn participant may not be assigned to another participant.

7.3. Lost to Follow-up

If a participant is lost to follow-up, every reasonable effort must be made by the study-site personnel to contact the participant and determine the reason for discontinuation/withdrawal. The measures taken to follow-up must be documented. Refer to Section 7.2, Participant Discontinuation/Withdrawal From the Study.

8. STUDY ASSESSMENTS AND PROCEDURES

Overview

Schedule of Activities (SoA) summarizes the frequency and timing of efficacy, PK, and safety measurements, and medical resource utilization (MRU) applicable to this study. During the Treatment Phase, study visits may occur within -3 ~ +2-day window. Visits for ^{99m}Tc bone scans or CT/MRI may occur up to 8 days before cycles requiring images. Unscheduled assessments can occur at any time as needed if signs of disease progression are observed.

Blood collections for PK should be kept as close to the specified time as possible (see Section 6.1). Other measurements may be done earlier than specified timepoints if needed. Actual dates and times of assessments will be recorded in the source documentation and eCRF.

For each participant, the maximum amount of blood drawn from each participant in this study will not exceed 13 mL per visit.

Repeat or unscheduled samples may be taken for safety reasons or for technical issues with the samples.

Sample Collection and Handling

The actual dates and times of sample collection must be recorded in the eCRF or laboratory requisition form.

Refer to the Schedule of Activities (SoA) for the timing and frequency of all sample collections.

Instructions for the collection, handling, storage, and shipment of samples are found in the laboratory manual that will be provided. Collection, handling, storage, and shipment of samples

must be under the specified, and where applicable, controlled temperature conditions as indicated in the laboratory manual.

Study-Specific Materials

The investigator will be provided with the following supplies:

- IB
- Pharmacy manual/study-site investigational product and procedures manual
- Laboratory manual
- National Cancer Institute - Common Terminology Criteria for Adverse Events (NCI-CTCAE) Version 4.03
- IWRS Manual
- electronic data capture (eDC) Manual
- eSource Manual
- Sample ICF.

8.1. Efficacy Assessments

Efficacy evaluations will be conducted as specified in the Schedule of Activities (SoA). The efficacy evaluations include the following:

- Serum PSA evaluation (for TTPP, as defined by PCWG2): PSA will be analyzed at the central laboratory. Results will be kept blinded to the participants, the Investigators, and the study team, in order to preserve the double-blind nature of this study. On Cycle 1 Day 1 only, the PSA evaluation does not need to be repeated if the Screening tests were done within 4 days of Cycle 1 Day 1.

  PSA evaluation for Cycle 3 Day 1, Cycle 4 Day 1, and Cycle 5 Day 1 should be collected no later than 1 week before each scheduled visit, eg, no later than Cycle 2 Day 21, Cycle 3 Day 21, and Cycle 4 Day 21 respectively. This will allow availability of laboratory results for determination of PSA progression status by each scheduled visit day (eg, Cycle 3 Day 1, Cycle 4 Day 1, and Cycle 5 Day 1) so that active treatment can be dispensed without delaying to the next scheduled visit if PSA progression has been confirmed for participants on Placebo arm.

  For all participants, at the time of unblinding, PSA data submitted to the central laboratory will no longer be blinded.

- Disease assessment (CT or MRI, bone scans): Disease assessments will be performed at baseline (screening: scans obtained prior to signing informed consent as part of the patient's standard of care may be allowed, provided the timing of the scans fall within the screening window), and at 16-week intervals thereafter.

  Imaging studies will include a CT scan of the chest, abdomen, and pelvis, plus a bone scan. At Screening, there will be an additional CT of the brain to rule out the presence of CNS metastases. For new bone lesions detected on bone scans, a second imaging modality (eg, CT or MRI) will be required to confirm progression.

The same method of assessment and the same technique should be used at Screening and during follow-up. Intravenous (IV) contrast is required when not medically contraindicated. Participants who have a contraindication to IV contrast may have MRI exams of brain, abdomen, and pelvis performed in lieu of CTs and a non-contrast CT of the chest. Tumor evaluation by positron emission tomography (PET) scan or by ultrasound or by prostate-specific membrane antigen (PSMA)-PET will not replace for standard chest, abdomen, and pelvis CT or MRI scans or bone scans as required by the study, but the CT portion of a PET/CT with sufficient quality which could meet the study requirement may be submitted in lieu of a dedicated CT.

The radiographic disease progression (appearance of distant metastasis) will be determined by the investigator. Participants who discontinue treatment before documented disease progression should continue with disease assessments during Posttreatment Follow-up Phase every 16 weeks (±7 days) until disease progression.

- Survival status, subsequent anticancer therapy, and SREs: These assessments will be performed during the Treatment and Posttreatment Follow-up Phase.

8.2. Safety Assessments

Details regarding the IDMC are provided in Committees Structure in Appendix 2: Regulatory, Ethical, and Study Oversight Considerations.

Adverse events will be reported and followed by the investigator as specified in Section 8.3, Adverse Events and Serious Adverse Events and Appendix 3: Adverse Events: Definitions and Procedures for Recording, Evaluating, Follow-up, and Reporting.

Any clinically relevant changes occurring during the study must be recorded on the AE section of the eCRF.

Any clinically significant abnormalities persisting at the end of the study/early withdrawal will be followed by the investigator until resolution or until a clinically stable condition is reached.

The study will include the following evaluations of safety and tolerability according to the time points provided in the Schedule of Activities (SoA).

8.2.1. Physical Examination

The screening physical examination will include, at a minimum, the general appearance of the participant, height, weight, and examination of the skin, ears, nose, throat, lungs, heart, abdomen, extremities, musculoskeletal system, lymphatic system, and nervous system. The screening physical examination will also include a full review of medical/oncological history and a complete examination of all organ systems. Screening physical examination abnormalities will be recorded in the eCRF under medical history, and post-baseline physical examination abnormalities will be recorded in the eCRF as AEs. After screening, limited symptom directed physical examinations and weight assessment will be conducted as per the Schedule of Activities (SoA).

8.2.2. Vital Signs

Temperature, pulse/heart rate, respiratory rate, and Blood pressure (BP) will be assessed during screening. At all other visits specified in the Schedule of Activities (SoA), only BP will be measured.

Blood pressure and pulse/heart rate measurements should be preceded by at least 5 minutes of rest in a quiet setting without distractions (eg, television, cell phones).

8.2.3. Electrocardiogram (ECG)

During the collection of ECGs, participants should be in a quiet setting without distractions (eg, television, cell phones). Participants should rest in a supine position for at least 5 minutes before ECG collection and should refrain from talking or moving arms or legs. If blood sampling or vital sign measurement is scheduled for the same time point as ECG recording, the procedures should be performed in the following order: ECG(s), vital signs, blood draw.

Standard 12-lead ECGs will be recorded at screening and as clinically indicated during study treatment. Clinically significant abnormalities noted at screening will be included in the medical/oncological history. Participants with ECG findings suspicious for a previously undiagnosed myocardial infarction will be evaluated and reassessed for eligibility.

8.2.4. Clinical Safety Laboratory Assessments

Blood samples for serum chemistry and hematology will be collected. The investigator must review the laboratory results, document this review, and record any clinically relevant changes occurring during the study in the AE section of the eCRF. The laboratory reports must be filed with the source documents.

The following tests will be performed by the central laboratory:

- Hematology Panel

-hemoglobin
-white blood cell (WBC) count
-platelet count

- Serum Chemistry Panel

-sodium
-potassium
-creatinine
-glucose (fasting)
-aspartate aminotransferase
-alanine aminotransferase
-bilirubin*

- high density lipoprotein-cholesterol (HDL-C) (fasting)
- low density lipoprotein-cholesterol (LDL-C) (fasting)
- triglycerides (fasting)

*: direct and indirect (at screening only if Gilbert's syndrome is suspected) and total bilirubin

- Other Laboratory Tests

-Testosterone
-TSH
- total T3, free T4 (direct) and Total T4 if TSH is not within normal limit

Hepatitis B surface antigen (HBsAg) and anti-hepatitis C virus (HCV) antibody **(at Screening only).** Hepatitis B virus (HBV)-DNA and HCV-ribonucleic acid (RNA) tests may be performed to rule out active infection at Screening as needed per the Investigator's discretion and approval from the sponsor.

In the event of additional safety monitoring, unscheduled laboratory assessments may be performed as required (and may be done locally when an immediate clinical decision needs to be made).

8.2.5. ECOG Performance Status

The ECOG-PS scale will be used to grade changes in the participant's daily living activities (Appendix 5: ECOG-PS Score). The frequency of ECOG-PS assessment is provided in Schedule of Activities (SoA).

8.3. Adverse Events and Serious Adverse Events

Timely, accurate, and complete reporting and analysis of safety information from clinical studies are crucial for the protection of participants, investigators, and the sponsor, and are mandated by regulatory agencies worldwide. The sponsor has established Standard Operating Procedures in conformity with regulatory requirements worldwide to ensure appropriate reporting of safety information; all clinical studies conducted by the sponsor or its affiliates will be conducted in accordance with those procedures.

Adverse events will be reported by the participant (or, when appropriate, by a caregiver, surrogate, or the participant's legally acceptable representative) for the duration of the study.

For further details on AEs and serious adverse events (SAEs) (Definitions and Classifications; Attribution Definitions; Severity Criteria; Special Reporting Situations; Procedures) as well as product quality complaints (PQC), refer to Appendix 3: Adverse Events: Definitions and Procedures for Recording, Evaluating, Follow-up, and Reporting.

8.3.1. Time Period and Frequency for Collecting Adverse Event and Serious Adverse Event Information

All Adverse Events

All AEs and special reporting situations, whether serious or nonserious, will be reported from the time a signed and dated ICF is obtained up to 30 days after the last dose of study intervention. Serious adverse events, including those spontaneously reported to the investigator within 30 days after the last dose of study intervention, must be reported using the Serious Adverse Event Form. The sponsor will evaluate any safety information that is spontaneously reported by an investigator beyond the time frame specified in the protocol.

Serious Adverse Events

All SAEs occurring during the study must be reported to the appropriate sponsor contact person by study-site personnel within 24 hours of their knowledge of the event.

Information regarding SAEs will be transmitted to the sponsor using the SAE Form, which must be completed and reviewed by a physician from the study site, and transmitted to the sponsor within 24 hours. The initial and follow-up reports of an SAE should be transmitted electronically or by facsimile (fax).

8.3.2. Method of Detecting Adverse Events and Serious Adverse Events

Care will be taken not to introduce bias when detecting AEs or SAEs. Open-ended and nonleading verbal questioning of the participant is the preferred method to inquire about AE occurrence.

8.3.3. Follow-up of Adverse Events and Serious Adverse Events

Adverse events, including pregnancy, will be followed by the investigator as specified in Appendix 3: Adverse Events: Definitions and Procedures for Recording, Evaluating, Follow-up, and Reporting.

8.3.4. Regulatory Reporting Requirements for Serious Adverse Events

The sponsor assumes responsibility for appropriate reporting of AEs to the regulatory authorities. The sponsor will also report to the investigator (and the head of the investigational institute where required) all suspected unexpected serious adverse reactions (SUSARs). The investigator (or sponsor where required) must report SUSARs to the appropriate Independent Ethics Committee/Institutional Review Board (IEC/IRB) that approved the protocol unless otherwise required and documented by the IEC/IRB.

8.3.5. Pregnancy

All initial reports of pregnancy in partners of male participants must be reported to the sponsor by the study-site personnel within 24 hours of their knowledge of the event using the appropriate pregnancy notification form. Abnormal pregnancy outcomes (eg, spontaneous abortion, fetal death, stillbirth, congenital anomalies, ectopic pregnancy) are considered SAEs and must be reported using the SAE Form.

Follow-up information regarding the outcome of the pregnancy and any postnatal sequelae in the infant will be required.

8.4. Treatment of Overdose

Not applicable, as overdose for apalutamide has not been defined, and there is no specific antidote to apalutamide.

8.5. Pharmacokinetics

Plasma samples will be used to evaluate the PK of apalutamide and its active metabolite N-desmethyl apalutamide. Participant confidentiality will be maintained.

8.5.1. Evaluations

Venous blood samples of approximately 2 mL will be collected for measurement of plasma concentrations of apalutamide and N-desmethyl apalutamide at approximately the same time for each sampling time point specified in the Schedule of Activities (SoA).

The dates and times for the doses administered 1 day preceding and during the PK sample day, and actual PK sampling dates and times must be recorded. Additional information about the collection, handling, and shipment of biological samples can be found in the Laboratory Manual. Collected samples must be stored under the specified and controlled conditions for the temperatures indicated in the laboratory manual.

8.5.2. Analytical Procedures

Pharmacokinetics

Plasma will be analyzed to determine concentrations of apalutamide and N-desmethyl apalutamide using a validated, specific, and sensitive liquid chromatography-tandem mass spectrometry method by or under the supervision of the sponsor.

8.5.3. Pharmacokinetic Parameters and Evaluations

Parameters

Based on the individual plasma concentration-time data, using the actual dose taken and the actual sampling times, PK parameters and exposure information of apalutamide and N-desmethyl apalutamide will be derived using population PK modelling. Details will be given in a separate population PK analysis plan and the results of the analysis will be presented in a separate report.

Exposure-Response Analysis

Exposure-response analysis between exposure and selective clinical endpoint will be explored if deemed necessary and with sufficient data.

8.6. Medical Resource Utilization and Health Economics

Medical resource utilization data, associated with medical encounters, will be collected in the eCRF by the Investigator and study-site personnel for all participants throughout the study. Protocol-mandated procedures, tests, and encounters are excluded. The data collected may be used to conduct exploratory economic analyses and will include:

- Number and duration of medical care encounters, including surgeries, and other selected procedures (inpatient and outpatient)
- Duration of hospitalization (total days length of stay, including duration by wards; eg, intensive care unit)
- Number and character of diagnostic and therapeutic tests and procedures.

Outpatient medical encounters and treatments (including physician or emergency room visits, tests and procedures, and medications).

9. STATISTICAL CONSIDERATIONS

Statistical analysis will be done by the sponsor or under the authority of the sponsor. A general description of the statistical methods to be used to analyze the efficacy and safety data is outlined below. Specific details will be provided in the Statistical Analysis Plan (SAP).

9.1. Sample Size Determination

Participants will be randomized in a 2:1 ratio to receive apalutamide or placebo. Participants randomized to the placebo arm and without any evidence of distant metastasis (according to imaging result at Cycle 5 Day 1, as well as any other relevant unscheduled visit) will start receiving active therapy of apalutamide at the time they have documented PSA progression (per PCWG2) or after completion of 5 cycles of placebo plus ADT treatment (handling by the IWRS).

It is assumed that failure distribution of the primary endpoint TTPP follows an exponential distribution with a constant hazard rate. The pivotal ARN-509-003 (SPARTAN) study suggested that East Asian participants (and study participants as a whole) treated with ADT only will have a median TTPP of 3.7 months.[4] It is estimated that approximately 75 participants will provide approximately 28 TTPP events at the time of the final analysis (approximately 5 months after last participant receiving first dose) which yields a power exceeding 95% for detecting an HR of 0.11 (median TTPP of 3.7 months for the placebo arm vs 33.6 months for the apalutamide arm) at a 2-tailed level of significance of 0.05. With a 32-month accrual period and an additional 5-months of follow-up, the study duration for the TTPP final analysis will be approximately 37 months.

9.2. Populations for Analyses

For purposes of analysis, the following populations are defined:

Population	Description
Intent-to-treat (ITT) Population	All eligible participants who are randomized into the study, with study drug assignments designated according to initial randomization, regardless of whether participants receive study drug or receive a different drug from that to which they were randomized to will be included in

	the analyses of all efficacy and clinical benefit endpoints and participant characteristics
Pharmacokinetic Population	All participants who receive at least 1 dose of study drug and have at least 1 available plasma concentration
Safety	All participants who receive at least 1 dose of study drug, with treatment assignments designated according to actual study treatment received will be the primary population for evaluating safety and treatment compliance and administration.

9.3. Statistical Analyses

9.3.1. Efficacy Analyses

All continuous variables will be summarized using number of participants (n), mean, standard deviation (SD), median, minimum, and maximum. Discrete variables will be summarized with n and percent. All efficacy endpoints will be analyzed using the ITT Population. Kaplan-Meier method and Cox model, including stratification factor at baseline, will be used to estimate the time-to-event variables and to obtain the HR along with the associated confidence intervals (CIs). Stratified log-rank test will be used as the primary analysis method to test the treatment effect for time-to-event variables. A participant without an event at the time of the analysis will be censored at the last known date the participant did not have a documented record of the corresponding event. Detailed censoring rules will be provided in the SAP. Response rate variable, ie, PSA response rate, will be evaluated using the Mantel-Haenszel test or the exact test if the frequencies are small. The stratification variable will be PSADT (>6 months vs ≤6 months) as documented in the IWRS.

The final analysis of TTPP will be performed at approximately 5 months after last participant receiving the first dose. TTPP will be tested using Stratified log-rank test, stratified by PSADT (>6 months vs ≤6 months), at a 2-tailed significance level of 0.05. The TTPP for control arm will be censored at the date of treatment switching for participants who switched treatment and had not PSA progressed at that time. For participants who received anticancer subsequent therapy before documented PSA progression, the TTPP will be censored at the last date of PSA assessment prior to the start of subsequent therapy. Kaplan-Meier method will be used to estimate the median TTPP, as well as the PSA progression-free rate at selected timepoints, and their 95% CIs. The estimation of HR and its 95% CI will be based on the stratified Cox proportional-hazard model. Non-stratified log-rank tests will also be provided as sensitivity analysis.

The analyses for exploratory time-to-event variables (MFS, OS, and time to symptomatic progression) will be performed descriptively, considering the early cross-over of control arm and the expected long medians for apalutamide arm suggested by study ARN-509-003 (SPARTAN), for participants who randomized to apalutamide arm.[2]

The MRU data may be used to conduct exploratory economic analyses separately.

Criteria

Not applicable.

9.3.2. Safety Analyses

Participants who receive at least 1 dose of study drug will be analyzed for safety. The safety parameters to be evaluated are the incidence, intensity, and type of AEs, vital signs measurements, and clinical laboratory results. Exposure to study drug and reasons for discontinuation of study treatment will be tabulated.

Adverse Events

The verbatim terms used in the eCRF by investigators to identify AEs will be coded using the Medical Dictionary for Regulatory Activities, Version 21.0 or later. and will be graded according to the NCI-CTCAE, Version 4.03. Treatment-emergent AEs are AEs that occur or worsen on or after first dose of study drug through 30 days after the last dose of study drug. All reported intervention-emergent AEs will be included in the analysis. For each AE, the percentage of participants who experience at least 1 occurrence of the given event will be summarized by intervention group. In addition, comparisons between intervention groups will be provided if appropriate.

Treatment-emergent AEs will be summarized by system organ class, preferred term, toxicity grade, and relationship to study drug. Serious adverse events and deaths will also be summarized. Summaries, listings, datasets, or participant narratives may be provided, as appropriate, for those participants who die, who discontinue intervention due to an AE, or who experience a severe or a SAE.

Clinical Laboratory Tests

Laboratory data will be summarized by type of laboratory test. Descriptive statistics will be calculated for each laboratory analyte at baseline and for observed values and changes from baseline at each scheduled time point. A listing of participants with any laboratory results outside the reference ranges will be provided. A listing of participants with any markedly abnormal laboratory results will also be provided. Normal ranges will be used to identify values that are outside the normal ranges and abnormal laboratory results will be graded according to the NCI-CTCAE Version 4.03. A shift summary of baseline grade by maximum post-baseline CTCAE grade will be presented. For each laboratory parameter, the baseline laboratory value will be defined as the last laboratory value collected on or prior to the date of the first dose of study drug. Participants who develop toxicities of Grade ≥3 will be listed.

Electrocardiogram

Electrocardiogram data will be summarized by ECG parameter at screening (baseline) only.

Vital Signs

Descriptive statistics of BP (systolic and diastolic) values and changes from baseline will be summarized at each scheduled time point. Descriptive statistics of other vital signs (body temperature, heart rate, respiratory rate) at baseline will also be summarized. The percentage of participants with values beyond clinically important limits will be summarized.

Physical Examination

Abnormal findings in physical examination will be recorded and summarized as AEs. Weight will be summarized.

9.3.3. Other Analyses

Pharmacokinetic Analyses

All plasma concentrations below the lowest quantifiable concentration or missing data will be labeled as such in the concentration data presentations or SAS dataset. Concentrations below the lower quantifiable concentration will be treated as zero in the summary statistics. Participants will be excluded from the PK analysis if their data do not allow for accurate assessment of the PK (eg, incomplete administration of the study drug; missing information of dosing and sampling times). All participants and samples excluded from the analysis will be clearly documented in the Clinical Study Report.

Descriptive statistics including arithmetic mean, SD, geometric mean, coefficient of variation, median, minimum, and maximum will be used to summarize plasma apalutamide and N-desmethyl apalutamide concentrations at each sampling time point.

Mean plasma apalutamide and N-desmethyl apalutamide concentration-time profile will be plotted after the first dose of study intervention. And individual plasma concentration-time profile may also be plotted.

Population PK analysis of plasma concentration-time data of apalutamide and N-desmethyl apalutamide will be performed using nonlinear mixed effects modeling. Details will be given in a population PK analysis plan and the results of the analysis will be presented in a separate report.

Exposure-Response Analyses

Exposure-response analysis between exposure and selective clinical endpoint (eg, incidence of rash) will be explored if deemed necessary and with sufficient data.

9.4. Interim Analysis

No interim analysis is planned for this study.

9.4.1. Independent Data Monitoring Committee

An IDMC will be established as noted in Committees Structure in Appendix 2: Regulatory, Ethical, and Study Oversight Considerations. The IDMC will be commissioned to review the efficacy and safety data at the final analysis. The IDMC will consist of at least 1 medical oncologist or urologist or both and at least 1 statistician. The IDMC's responsibilities, authorities, and procedures will be documented in the IDMC charter.

10. SUPPORTING DOCUMENTATION AND OPERATIONAL CONSIDERATIONS

10.1. Appendix 1: Abbreviations

ADT	androgen deprivation therapy
AEs	adverse events
ALT	alanine aminotransferase
ANC	absolute neutrophil count
AR	androgen receptor
AST	aspartate aminotransferase
AUC	area uder the plasma concentration
BICR	blinded independent central review
BP	blood pressure
BSA	body surface area
Cis	confidence intervals
CNS	central nervous system
CRF	case report form(s) (electronic as appropriate for this study)
CRPC	Castration-resistant prostate cancer
CT	computed tomography
ECG	electrocardiogram
ECOG-PS	Eastern Cooperative Oncology Group – performance status
eDC	electronic data capture
EoT	end-of-treatment
FACT-G	Functional Assessment Cancer Therapy-General
FACT-P	Functional Assessment of Cancer Therapy-Prostate
GCP	Good Clinical Practice
GnRHa	gonadotropin releasing hormone analog
HBsAg	hepatitis B surface antigen
HCV	hepatitis C virus
HDL-C	high density lipoprotein-cholesterol
HBV	hepatitis B virus
HR	hazard ratio
IB	investigator's brochure
ICF	informed consent form
ICH	International Council for Harmonisation
IEC	Independent Ethics Committee
IDMC	independent data monitoring committee
IRB	Institutional Review Board
IWRS	interactive web response system
LDL-C	low density lipoprotein-cholesterol
MedDRA	Medical Dictionary for Regulatory Activities
MFS	metastasis-free survival
MSKCC	Memorial Sloan-Kettering Cancer Center
MRI	magnetic resonance imaging
MRU	medical resource utilization
NCI-CTCAE	National Cancer Institute-Common Terminology Criteria for Adverse Events
NM-CRPC	Non-Metastatic Castration-Resistant Prostate Cancer
OS	overall survival
PAC	post approval commitment
PC	prostate cancer
PCWG2	Prostate Cancer Working Group 2
PFS	progression-free survival
PFS2	progression-free survival with the first subsequent therapy
PK	pharmacokinetic(s)
PQC	Product Quality Complaint
PSA	prostate-specific antigen
PSADT	PSA doubling time

SAP	statistical analysis plan
SD	standard deviation
SGOT	serum glutamic oxaloacetic transaminase
SGPT	serum glutamic pyruvic transaminase
SoA	schedule of activities
SRE	skeletal-related events
SUSAR	suspected unexpected serious adverse reaction
TEAE	Treatment-emergent adverse event
TSH	thyroid stimulating hormone
TTM	time to metastasis
TTPP	time to prostate-specific antigen progression
TURP	transurethral resection of the prostate
ULN	upper limit of normal
VAS	visual analog scale
Versus	vs

Definitions of Terms

Electronic source system	Contains data traditionally maintained in a hospital or clinic record to document medical care or data recorded in a eCRF as determined by the protocol. Data in this system may be considered source documentation.

10.2. Appendix 2: Regulatory, Ethical, and Study Oversight Considerations

REGULATORY AND ETHICAL CONSIDERATIONS

Investigator Responsibilities

The investigator is responsible for ensuring that the study is performed in accordance with the protocol, current International Council for Harmonisation (ICH) guidelines on Good Clinical Practice (GCP), and applicable regulatory and country-specific requirements.

Good Clinical Practice is an international ethical and scientific quality standard for designing, conducting, recording, and reporting studies that involve the participation of human participants. Compliance with this standard provides public assurance that the rights, safety, and well-being of study participants are protected, consistent with the principles that originated in the Declaration of Helsinki, and that the study data are credible.

Protocol Amendments

Neither the investigator nor the sponsor will modify this protocol without a formal amendment by the sponsor. All protocol amendments must be issued by the sponsor, and signed and dated by the investigator. Protocol amendments must not be implemented without prior IEC/IRB approval, or when the relevant competent authority has raised any grounds for non-acceptance, except when necessary to eliminate immediate hazards to the participants, in which case the amendment must be promptly submitted to the IEC/IRB and relevant competent authority. Documentation of amendment approval by the investigator and IEC/IRB must be provided to the sponsor. When the change(s) involve only logistic or administrative aspects of the study, the IEC/IRB (where required) only needs to be notified.

During the course of the study, in situations where a departure from the protocol is unavoidable, the investigator or other physician in attendance will contact the appropriate sponsor representative listed in the Contact Information page(s), which will be provided as a separate document. Except in emergency situations, this contact should be made before implementing any departure from the protocol. In all cases, contact with the sponsor must be made as soon as possible to discuss the situation and agree on an appropriate course of action. The data recorded in the eCRF and source documents will reflect any departure from the protocol, and the source documents will describe this departure and the circumstances requiring it.

Regulatory Approval/Notification

This protocol and any amendment(s) must be submitted to the appropriate regulatory authorities in each respective country, if applicable. A study may not be initiated until all local regulatory requirements are met.

Required Prestudy Documentation

The following documents must be provided to the sponsor before shipment of study intervention to the study site:

- Protocol and amendment(s), if any, signed and dated by the principal investigator
- A copy of the dated and signed (or sealed, where appropriate per local regulations), written IEC/IRB approval of the protocol, amendments, ICF, any recruiting materials, and if applicable, participant compensation programs. This approval must clearly identify the specific protocol by title and number and must be signed (or sealed, where appropriate per local regulations) by the chairman or authorized designee.
- Name and address of the IEC/IRB, including a current list of the IEC/IRB members and their function, with a statement that it is organized and operates according to GCP and the applicable laws and regulations. If accompanied by a letter of explanation, or equivalent, from the IEC/IRB, a general statement may be substituted for this list. If an investigator or a member of the study-site personnel is a member of the IEC/IRB, documentation must be obtained to state that this person did not participate in the deliberations or in the vote/opinion of the study.
- Regulatory authority approval or notification, if applicable
- Signed and dated statement of investigator (eg, Form FDA 1572), if applicable
- Documentation of investigator qualifications (eg, curriculum vitae)
- Completed investigator financial disclosure form from the principal investigator, where required
- Signed and dated clinical trial agreement, which includes the financial agreement
- Any other documentation required by local regulations

The following documents must be provided to the sponsor before enrollment of the first participant:

- Completed investigator financial disclosure forms from all subinvestigators, if applicable
- Documentation of subinvestigator qualifications (eg, curriculum vitae)
- Name and address of any local laboratory conducting tests for the study, and a dated copy of current laboratory normal ranges for these tests, if applicable
- Local laboratory documentation demonstrating competence and test reliability (eg, accreditation/license), if applicable

Independent Ethics Committee or Institutional Review Board

Before the start of the study, the investigator (or sponsor where required) will provide the IEC/IRB with current and complete copies of the following documents (as required by local regulations):

- Final protocol and, if applicable, amendments
- Sponsor-approved ICF (and any other written materials to be provided to the participants)
- Investigator's Brochure (or equivalent information) and amendments/addenda
- Sponsor-approved participant recruiting materials

- Information on compensation for study-related injuries or payment to participants for participation in the study, if applicable
- Investigator's curriculum vitae or equivalent information (unless not required, as documented by the IEC/IRB)
- Information regarding funding, name of the sponsor, institutional affiliations, other potential conflicts of interest, and incentives for participants
- Any other documents that the IEC/IRB requests to fulfill its obligation

This study will be undertaken only after the IEC/IRB has given full approval of the final protocol, amendments (if any, excluding the ones that are purely administrative, with no consequences for participants, data or study conduct, unless required locally), the ICF, applicable recruiting materials, and participant compensation programs, and the sponsor has received a copy of this approval. This approval letter must be dated and must clearly identify the IEC/IRB and the documents being approved.

During the study the investigator (or sponsor where required) will send the following documents and updates to the IEC/IRB for their review and approval, where appropriate:

- Protocol amendments (excluding the ones that are purely administrative, with no consequences for participants, data or study conduct)
- Revision(s) to ICF and any other written materials to be provided to participants
- If applicable, new or revised participant recruiting materials approved by the sponsor
- Revisions to compensation for study-related injuries or payment to participants for participation in the study, if applicable
- New edition(s) of the Investigator's Brochure and amendments/addenda
- Summaries of the status of the study at intervals stipulated in guidelines of the IEC/IRB (at least annually)
- Reports of AEs that are serious, unlisted/unexpected, and associated with the study intervention
- New information that may adversely affect the safety of the participants or the conduct of the study
- Deviations from or changes to the protocol to eliminate immediate hazards to the participants
- Report of deaths of participants under the investigator's care
- Notification if a new investigator is responsible for the study at the site
- Development Safety Update Report and Line Listings, where applicable
- Any other requirements of the IEC/IRB

For all protocol amendments (excluding the ones that are purely administrative, with no consequences for participants, data or study conduct), the amendment and applicable ICF

revisions must be submitted promptly to the IEC/IRB for review and approval before implementation of the change(s).

At least once a year, the IEC/IRB will be asked to review and reapprove this study, where required.

At the end of the study, the investigator (or sponsor where required) will notify the IEC/IRB about the study completion (if applicable, the notification will be submitted through the head of investigational institution).

Other Ethical Considerations

For study-specific ethical design considerations, refer to Section 4.2.1.

FINANCIAL DISCLOSURE

If applicable, investigators and subinvestigators will provide the sponsor with sufficient, accurate financial information in accordance with local regulations to allow the sponsor to submit complete and accurate financial certification or disclosure statements to the appropriate regulatory authorities. Investigators are responsible for providing information on financial interests during the course of the study and for 1 year after completion of the study.

Refer to Required Prestudy Documentation (above) for details on financial disclosure.

INFORMED CONSENT

Each participant (or a legally acceptable representative) must give written consent according to local requirements after the nature of the study has been fully explained. The ICF(s) must be signed before performance of any study-related activity. The ICF(s) that is/are used must be approved by both the sponsor and by the reviewing IEC/IRB and be in a language that the participant can read and understand. The informed consent should be in accordance with principles that originated in the Declaration of Helsinki, current ICH and GCP guidelines, applicable regulatory requirements, and sponsor policy.

Before enrollment in the study, the investigator or an authorized member of the study-site personnel must explain to potential participants or their legally acceptable representatives the aims, methods, reasonably anticipated benefits, and potential hazards of the study, and any discomfort participation in the study may entail. Participants will be informed that their participation is voluntary and that they may withdraw consent to participate at any time. They will be informed that choosing not to participate will not affect the care the participant will receive for the treatment of his disease. Participants will be told that alternative treatments are available if they refuse to take part and that such refusal will not prejudice future treatment. Finally, they will be told that the investigator will maintain a participant identification register for the purposes of long-term follow-up if needed and that their records may be accessed by health authorities and authorized sponsor personnel without violating the confidentiality of the participant, to the extent permitted by the applicable law(s) or regulations. By signing the ICF the participant or legally acceptable representative is authorizing such access, which includes

permission to obtain information about his survival status. It also denotes that the participant agrees to allow his study physician to recontact the participant for the purpose of obtaining consent for additional safety evaluations, and subsequent disease-related treatments, if needed.

The participant or legally acceptable representative will be given sufficient time to read the ICF and the opportunity to ask questions. After this explanation and before entry into the study, consent should be appropriately recorded by means of either the participant's or his legally acceptable representative's personally dated signature. After having obtained the consent, a copy of the ICF must be given to the participant.

If the participant or legally acceptable representative is unable to read or write, an impartial witness should be present for the entire informed consent process (which includes reading and explaining all written information) and should personally date and sign the ICF after the oral consent of the participant or legally acceptable representative is obtained.

When prior consent of the participant is not possible and the participant's legally acceptable representative is not available, enrollment procedures should be described in the protocol with documented approval/favorable opinion by the IEC/IRB to protect the rights, safety, and well-being of the participant and to ensure compliance with applicable regulatory requirements. The participant or legally acceptable representative must be informed about the study as soon as possible and give consent to continue.

DATA PROTECTION

Privacy of Personal Data

The collection and processing of personal data from participants enrolled in this study will be limited to those data that are necessary to fulfill the objectives of the study.

These data must be collected and processed with adequate precautions to ensure confidentiality and compliance with applicable data privacy protection laws and regulations. Appropriate technical and organizational measures to protect the personal data against unauthorized disclosures or access, accidental or unlawful destruction, or accidental loss or alteration must be put in place. Sponsor personnel whose responsibilities require access to personal data agree to keep the identity of participants confidential.

The informed consent obtained from the participant (or his legally acceptable representative) includes explicit consent for the processing of personal data and for the investigator/institution to allow direct access to his original medical records (source data/documents) for study-related monitoring, audit, IEC/IRB review, and regulatory inspection. This consent also addresses the transfer of the data to other entities and to other countries.

The participant has the right to request through the investigator access to his personal data and the right to request rectification of any data that are not correct or complete. Reasonable steps will be taken to respond to such a request, taking into consideration the nature of the request, the conditions of the study, and the applicable laws and regulations.

Exploratory PK research is not conducted under standards appropriate for the return of data to participants. In addition, the sponsor cannot make decisions as to the significance of any findings resulting from exploratory research. Therefore, exploratory research data will not be returned to participants or investigators, unless required by law or local regulations. Privacy and confidentiality of data generated in the future on stored samples will be protected by the same standards applicable to all other clinical data.

COMMITTEES STRUCTURE

Independent Data Monitoring Committee

An IDMC will be established to review the efficacy and safety data at the final analysis. This committee will consist of at least 1 medical expert in the relevant therapeutic area and at least 1 statistician; committee membership responsibilities, authorities, and procedures will be documented in its charter.

PUBLICATION POLICY/DISSEMINATION OF CLINICAL STUDY DATA

All information, including but not limited to information regarding apalutamide or the sponsor's operations (eg, patent application, formulas, manufacturing processes, basic scientific data, prior clinical data, formulation information) supplied by the sponsor to the investigator and not previously published, and any data, including exploratory research data, generated as a result of this study, are considered confidential and remain the sole property of the sponsor. The investigator agrees to maintain this information in confidence and use this information only to accomplish this study,and will not use it for other purposes without the sponsor's prior written consent.

The investigator understands that the information developed in the study will be used by the sponsor in connection with the continued development of apalutamide, and thus may be disclosed as required to other clinical investigators or regulatory agencies. To permit the information derived from the clinical studies to be used, the investigator is obligated to provide the sponsor with all data obtained in the study.

The results of the study will be reported in a Clinical Study Report generated by the sponsor and will contain data from all study sites that participated in the study as per protocol. Recruitment performance or specific expertise related to the nature and the key assessment parameters of the study will be used to determine a coordinating investigator for the study. Results of exploratory analyses performed after the Clinical Study Report has been issued will be reported in a separate report and will not require a revision of the Clinical Study Report.

Study participant identifiers will not be used in publication of results. Any work created in connection with performance of the study and contained in the data that can benefit from copyright protection (except any publication by the investigator as provided for below) shall be the property of the sponsor as author and owner of copyright in such work.

Consistent with Good Publication Practices and International Committee of Medical Journal Editors (ICMJE) guidelines, the sponsor shall have the right to publish such primary (multicenter)

data and information without approval from the investigator. The investigator has the right to publish study-site-specific data after the primary data are published. If an investigator wishes to publish information from the study, a copy of the manuscript must be provided to the sponsor for review at least 60 days before submission for publication or presentation. Expedited reviews will be arranged for abstracts, poster presentations, or other materials. If requested by the sponsor in writing, the investigator will withhold such publication for up to an additional 60 days to allow for filing of a patent application. In the event that issues arise regarding scientific integrity or regulatory compliance, the sponsor will review these issues with the investigator. The sponsor will not mandate modifications to scientific content and does not have the right to suppress information. For multicenter study designs and substudy approaches, secondary results generally should not be published before the primary endpoints of a study have been published. Similarly, investigators will recognize the integrity of a multicenter study by not submitting for publication data derived from the individual study site until the combined results from the completed study have been submitted for publication, within 18 months after the study end date, or the sponsor confirms there will be no multicenter study publication. Authorship of publications resulting from this study will be based on the guidelines on authorship, such as those described in the ICMJE Recommendations for the Conduct, Reporting, Editing and Publication of Scholarly Work in Medical Journals, which state that the named authors must have made a significant contribution to the conception or design of the work; or the acquisition, analysis, or interpretation of the data for the work; and drafted the work or revised it critically for important intellectual content; and given final approval of the version to be published; and agreed to be accountable for all aspects of the work in ensuring that questions related to the accuracy or integrity of any part of the work are appropriately investigated and resolved.

Registration of Clinical Studies and Disclosure of Results

The sponsor will register and disclose the existence of and the results of clinical studies as required by law.

DATA QUALITY ASSURANCE

Data Quality Assurance/Quality Control

Steps to be taken to ensure the accuracy and reliability of data include the selection of qualified investigators and appropriate study sites, review of protocol procedures with the investigator and study-site personnel before the study, and periodic monitoring visits by the sponsor, and direct transmission of clinical laboratory data from a central laboratory into the sponsor's database. Written instructions will be provided for collection, handling, storage, and shipment of samples.

Guidelines for eCRF completion will be provided and reviewed with study-site personnel before the start of the study. The sponsor will review eCRF for accuracy and completeness during on-site monitoring visits and after transmission to the sponsor; any discrepancies will be resolved with the investigator or designee, as appropriate. After upload of the data into the study database they will be verified for accuracy and consistency with the data sources.

CASE REPORT FORM COMPLETION

Case report forms are prepared and provided by the sponsor for each participant in electronic format. All data relating to the study must be recorded in eCRF. All eCRF entries, corrections, and alterations must be made by the investigator or authorized study-site personnel. The investigator must verify that all data entries in the eCRF are accurate and correct.

The study data will be transcribed by study-site personnel from the source documents onto an electronic eCRF, if applicable. Study-specific data will be transmitted in a secure manner to the sponsor.

Worksheets may be used for the capture of some data to facilitate completion of the eCRF. Any such worksheets will become part of the participant's source documents. Data must be entered into eCRF in English. The eCRF must be completed as soon as possible after a participant visit and the forms should be available for review at the next scheduled monitoring visit.

All participative measurements (eg, pain scale information or other questionnaires) will be completed by the same individual who made the initial baseline determinations whenever possible.

If necessary, queries will be generated in the eDC tool. If corrections to a eCRF are needed after the initial entry into the eCRF, this can be done in either of the following ways:

- Investigator and study-site personnel can make corrections in the eDC tool at their own initiative or as a response to an auto query (generated by the eDC tool).
- Sponsor or sponsor delegate can generate a query for resolution by the investigator and study-site personnel.

SOURCE DOCUMENTS

At a minimum, source documents consistent in the type and level of detail with that commonly recorded at the study site as a basis for standard medical care must be available for the following: participant identification, eligibility, and study identification; study discussion and date of signed informed consent; dates of visits; results of safety and efficacy parameters as required by the protocol; record of all AEs and follow-up of AEs; concomitant medication; intervention receipt/dispensing/return records; study intervention administration information; and date of study completion and reason for early discontinuation of study intervention or withdrawal from the study, if applicable.

The author of an entry in the source documents should be identifiable.

Specific details required as source data for the study and source data collection methods will be reviewed with the investigator before the study and will be described in the monitoring guidelines (or other equivalent document).

The following data will be recorded directly into the eCRF and will be considered source data:

- Race
- BP and pulse/heart rate
- Height and weight
- Details of physical examination

The minimum source documentation requirements for Section 5.1, Inclusion Criteria and Section 5.2, Exclusion Criteria that specify a need for documented medical history are as follows:

- Referral letter from treating physician or
- Complete history of medical notes at the site
- Discharge summaries.

Inclusion and exclusion criteria not requiring documented medical history must be verified at a minimum by participant interview or other protocol required assessment (eg, physical examination, laboratory assessment) and documented in the source documents.

An eSource system may be utilized, which contains data traditionally maintained in a hospital or clinic record to document medical care (eg, electronic source documents) as well as the clinical study-specific data fields as determined by the protocol. This data is electronically extracted for use by the sponsor. If eSource is utilized, references made to the eCRF in the protocol include the eSource system but information collected through eSource may not be limited to that found in the eCRF.

MONITORING

The sponsor will use a combination of monitoring techniques central, remote, or on-site monitoring to monitor this study.

The sponsor will perform on-site monitoring visits as frequently as necessary. The monitor will record dates of the visits in a study site visit log that will be kept at the study site. The first post-initiation visit will be made as soon as possible after enrollment has begun. At these visits, the monitor will compare the data entered into the eCRF with the source documents (eg, hospital/clinic/physician's office medical records). The nature and location of all source documents will be identified to ensure that all sources of original data required to complete the eCRF are known to the sponsor and study-site personnel and are accessible for verification by the sponsor study-site contact. If electronic records are maintained at the study site, the method of verification must be discussed with the study-site personnel.

Direct access to source documents (medical records) must be allowed for the purpose of verifying that the recorded data are consistent with the original source data. Findings from this review will be discussed with the study-site personnel. The sponsor expects that, during monitoring visits, the relevant study-site personnel will be available, the source documents will be accessible, and a suitable environment will be provided for review of study-related documents.

The monitor will meet with the investigator on a regular basis during the study to provide feedback on the study conduct.

In addition to on-site monitoring visits, remote contacts can occur. It is expected that during these remote contacts, study-site personnel will be available to provide an update on the progress of the study at the site.

Central monitoring will take place for data identified by the sponsor as requiring central review.

ON-SITE AUDITS

Representatives of the sponsor's clinical quality assurance department may visit the study site at any time during or after completion of the study to conduct an audit of the study in compliance with regulatory guidelines and company policy. These audits will require access to all study records, including source documents, for inspection. Participant privacy must, however, be respected. The investigator and study-site personnel are responsible for being present and available for consultation during routinely scheduled study-site audit visits conducted by the sponsor or its designees.

Similar auditing procedures may also be conducted by agents of any regulatory body, either as part of a national GCP compliance program or to review the results of this study in support of a regulatory submission. The investigator should immediately notify the sponsor if he or she has been contacted by a regulatory agency concerning an upcoming inspection.

RECORD RETENTION

In compliance with the ICH/GCP guidelines, the investigator/institution will maintain all eCRF and all source documents that support the data collected from each participant, as well as all study documents as specified in ICH/GCP Section 8, Essential Documents for the Conduct of a Clinical Trial, and all study documents as specified by the applicable regulatory requirement(s). The investigator/institution will take measures to prevent accidental or premature destruction of these documents.

Essential documents must be retained until at least 2 years after the last approval of a marketing application in an ICH region and until there are no pending or contemplated marketing applications in an ICH region or until at least 2 years have elapsed since the formal discontinuation of clinical development of the investigational product. These documents will be retained for a longer period if required by the applicable regulatory requirements or by an agreement with the sponsor. It is the responsibility of the sponsor to inform the investigator/institution as to when these documents no longer need to be retained.

If the responsible investigator retires, relocates, or for other reasons withdraws from the responsibility of keeping the study records, custody must be transferred to a person who will accept the responsibility. The sponsor must be notified in writing of the name and address of the new custodian. Under no circumstance shall the investigator relocate or dispose of any study documents before having obtained written approval from the sponsor.

If it becomes necessary for the sponsor or the appropriate regulatory authority to review any documentation relating to this study, the investigator/institution must permit access to such reports.

STUDY AND SITE CLOSURE

Study Termination

The sponsor reserves the right to close the study site or terminate the study at any time for any reason at the sole discretion of the sponsor. Study sites will be closed upon study completion. A study site is considered closed when all required documents and study supplies have been collected and a study-site closure visit has been performed.

The investigator may initiate study-site closure at any time, provided there is reasonable cause and sufficient notice is given in advance of the intended termination.

Reasons for the early closure of a study site by the sponsor or investigator may include but are not limited to:

- Failure of the investigator to comply with the protocol, the requirements of the IEC/IRB or local health authorities, the sponsor's procedures, or GCP guidelines
- Inadequate recruitment of participants by the investigator
- Discontinuation of further study intervention development

10.3. Appendix 3: Adverse Events: Definitions and Procedures for Recording, Evaluating, Follow-up, and Reporting

ADVERSE EVENT DEFINITIONS AND CLASSIFICATIONS

Adverse Event

An AE is any untoward medical occurrence in a clinical study participant administered a medicinal (investigational or non-investigational) product. An AE does not necessarily have a causal relationship with the intervention. An AE can therefore be any unfavorable and unintended sign (including an abnormal finding), symptom, or disease temporally associated with the use of a medicinal (investigational or non-investigational) product, whether or not related to that medicinal (investigational or non-investigational) product. (Definition per ICH).

This includes any occurrence that is new in onset or aggravated in severity or frequency from the baseline condition, or abnormal results of diagnostic procedures, including laboratory test abnormalities.

Note: The sponsor collects AEs starting with the signing of the ICF (refer to All AEs under Section 8.3.1, Time Period and Frequency for Collecting Adverse Events and Serious Adverse Events Information, for time of last AE recording).

Serious Adverse Event

A SAE based on ICH and EU Guidelines on Pharmacovigilance for Medicinal Products for Human Use is any untoward medical occurrence that at any dose:

- Results in death
- Is life-threatening
  (The participant was at risk of death at the time of the event. It does not refer to an event that hypothetically might have caused death if it were more severe.)
- Requires inpatient hospitalization or prolongation of existing hospitalization
- Results in persistent or significant disability/incapacity
- Is a congenital anomaly/birth defect
- Is a suspected transmission of any infectious agent via a medicinal product
- Is Medically Important*

*Medical and scientific judgment should be exercised in deciding whether expedited reporting is also appropriate in other situations, such as important medical events that may not be immediately life-threatening or result in death or hospitalization but may jeopardize the participant or may require intervention to prevent 1 of the other outcomes listed in the definition above. These should usually be considered serious.

Unlisted (Unexpected) Adverse Event/Reference Safety Information

An AE is considered unlisted if the nature or severity is not consistent with the applicable product reference safety information. For apalutamide, the expectedness of an AE will be determined by whether or not it is listed in the Investigator's Brochure. For ADT (GnRHa choice based on investigator's decision), the expectedness of an AE will be determined by whether or not it is listed in the specific GnRHa package insert.

Adverse Event Associated With the Use of the Intervention

An AE is considered associated with the use of the intervention if the attribution is possible, probable, or very likely by the definitions listed below (see Attribution Definitions).

ATTRIBUTION DEFINITIONS

Not Related

An AE that is not related to the use of the intervention.

Doubtful

An AE for which an alternative explanation is more likely, eg, concomitant treatment(s), concomitant disease(s), or the relationship in time suggests that a causal relationship is unlikely.

Possible

An AE that might be due to the use of the intervention. An alternative explanation, eg, concomitant treatment(s), concomitant disease(s), is inconclusive. The relationship in time is reasonable; therefore, the causal relationship cannot be excluded.

Probable

An AE that might be due to the use of the intervention. The relationship in time is suggestive (eg, confirmed by dechallenge). An alternative explanation is less likely, eg, concomitant treatment(s), concomitant disease(s).

Very Likely

An AE that is listed as a possible adverse reaction and cannot be reasonably explained by an alternative explanation, eg, concomitant treatment(s), concomitant disease(s). The relationship in time is very suggestive (eg, it is confirmed by dechallenge and rechallenge).

SEVERITY CRITERIA

The severity assessment for an AE or SAE should be completed using NCI-CTCAE Version 4.03. Any AE or SAE not listed in NCI-CTCAE Version 4.03 will be graded according to investigator clinical judgment by using the standard grades as follows:

Grade 1 (Mild): Awareness of symptoms that are easily tolerated, causing minimal discomfort and not interfering with everyday activities.

Grade 2 (Moderate): Sufficient discomfort is present to cause interference with normal activity.

Grade 3 (Severe): Extreme distress, causing significant impairment of functioning or incapacitation. Prevents normal everyday activities.

Grade 4: Life-threatening or disabling AE

Grade 5: Death related to the AE.

The investigator should use clinical judgment in assessing the severity of events not directly experienced by the participant (eg, laboratory abnormalities).

SPECIAL REPORTING SITUATIONS

Safety events of interest on a sponsor study intervention in an interventional study that may require expedited reporting or safety evaluation include, but are not limited to:

- Overdose of a sponsor study intervention
- Suspected abuse/misuse of a sponsor study intervention
- Accidental or occupational exposure to a sponsor study intervention
- Any failure of expected pharmacologic action (ie, lack of effect) of a sponsor study intervention
- Unexpected therapeutic or clinical benefit from use of a sponsor study intervention
- Medication error involving a sponsor product (with or without participant/patient exposure to the sponsor study intervention, eg, name confusion)
- Exposure to a sponsor study intervention from breastfeeding

Special reporting situations should be recorded in the eCRF. Any special reporting situation that meets the criteria of an SAE should be recorded on the SAE page of the eCRF.

PROCEDURES

All Adverse Events

All AEs, regardless of seriousness, severity, or presumed relationship to study intervention, must be recorded using medical terminology in the source document and the eCRF. Whenever possible, diagnoses should be given when signs and symptoms are due to a common etiology (eg, cough, runny nose, sneezing, sore throat, and head congestion should be reported as "upper respiratory infection"). Investigators must record in the eCRF their opinion concerning the relationship of the AE to study therapy. All measures required for AE management must be recorded in the source document and reported according to sponsor instructions.

For all studies with an outpatient phase, including open-label studies, the participant must be provided with a "wallet (study) card" and instructed to carry this card with them for the duration of the study indicating the following:

- Study number
- Statement, in the local language(s), that the participant is participating in a clinical study
- Investigator's name and 24-hour contact telephone number
- Local sponsor's name and 24-hour contact telephone number (for medical staff only)
- Site number
- Participant number
- Any other information that is required to do an emergency breaking of the blind.

Serious Adverse Events

All SAE that have not resolved by the end of the study, or that have not resolved upon discontinuation of the participant's participation in the study, must be followed until any of the following occurs:

- The event resolves
- The event stabilizes
- The event returns to baseline, if a baseline value/status is available
- The event can be attributed to agents other than the study intervention or to factors unrelated to study conduct
- It becomes unlikely that any additional information can be obtained (participant or health care practitioner refusal to provide additional information, lost to follow-up after demonstration of due diligence with follow-up efforts)

Suspected transmission of an infectious agent by a medicinal product will be reported as a SAE. Any event requiring hospitalization (or prolongation of hospitalization) that occurs during the course of a participant's participation in a study must be reported as a SAE, except hospitalizations for the following:

- Hospitalizations not intended to treat an acute illness or AE (eg, social reasons such as pending placement in long-term care facility)
- Surgery or procedure planned before entry into the study (must be documented in the eCRF). Note: Hospitalizations that were planned before the signing of the ICF, and where the underlying condition for which the hospitalization was planned has not worsened, will not be considered SAEs. Any AE that results in a prolongation of the originally planned hospitalization is to be reported as a new SAE.
- For convenience the investigator may choose to hospitalize the participant for the duration of the intervention period.

Disease progression should not be recorded as an AE or SAE term; instead, signs and symptoms of clinical sequelae resulting from disease progression/lack of efficacy will be reported if they fulfill the SAE definition (refer to AE Definitions and Classifications in Appendix 3: Adverse Events: Definitions and Procedures for Recording, Evaluating, Follow-up, and Reporting).

CONTACTING SPONSOR REGARDING SAFETY

The names (and corresponding telephone numbers) of the individuals who should be contacted regarding safety issues or questions regarding the study are listed in the Contact Information page(s), which will be provided as a separate document.

PRODUCT QUALITY COMPLAINT HANDLING

A PQC is defined as any suspicion of a product defect related to manufacturing, labeling, or packaging, ie, any dissatisfaction relative to the identity, quality, durability, or reliability of a product, including its labeling or package integrity. A PQC may have an impact on the safety and efficacy of the product. Timely, accurate, and complete reporting and analysis of PQC information from studies are crucial for the protection of participants, investigators, and the sponsor, and are mandated by regulatory agencies worldwide. The sponsor has established procedures in conformity with regulatory requirements worldwide to ensure appropriate reporting of PQC information; all studies conducted by the sponsor or its affiliates will be conducted in accordance with those procedures.

Procedures

All initial PQCs must be reported to the sponsor by the study-site personnel within 24 hours after being made aware of the event.

If the defect is combined with a SAE, the study-site personnel must report the PQC to the sponsor according to the SAE reporting timelines (refer to Section 8.3.1, Time Period and Frequency for Collecting Adverse Event and Serious Adverse Event Information). A sample of the suspected product should be maintained for further investigation if requested by the sponsor.

Contacting Sponsor Regarding Product Quality

The names (and corresponding telephone numbers) of the individuals who should be contacted regarding product quality issues are listed in the Contact Information page(s), which will be provided as a separate document.

10.4. Appendix 4: Liver Safety: Suggested Actions and Follow-up Assessments

Guideline Algorithm for Monitoring, Assessment & Evaluation of Abnormal Liver Tests in Participants with no Underlying Liver Disease and normal baseline ALT, AST, Alkaline Phosphatase and Bilirubin

Although this algorithm is still applicable across all populations, it has been developed assuming normal liver function at baseline. For populations with preexisting liver disease and/or AT increases at baseline, product teams are strongly encouraged to consult with Hepatic Safety Group for further guidance particularly for discontinuation criteria.

NOTE: "Liver tests" or "LT's" is the proper name for what are often called "liver function tests" or "LFT's"

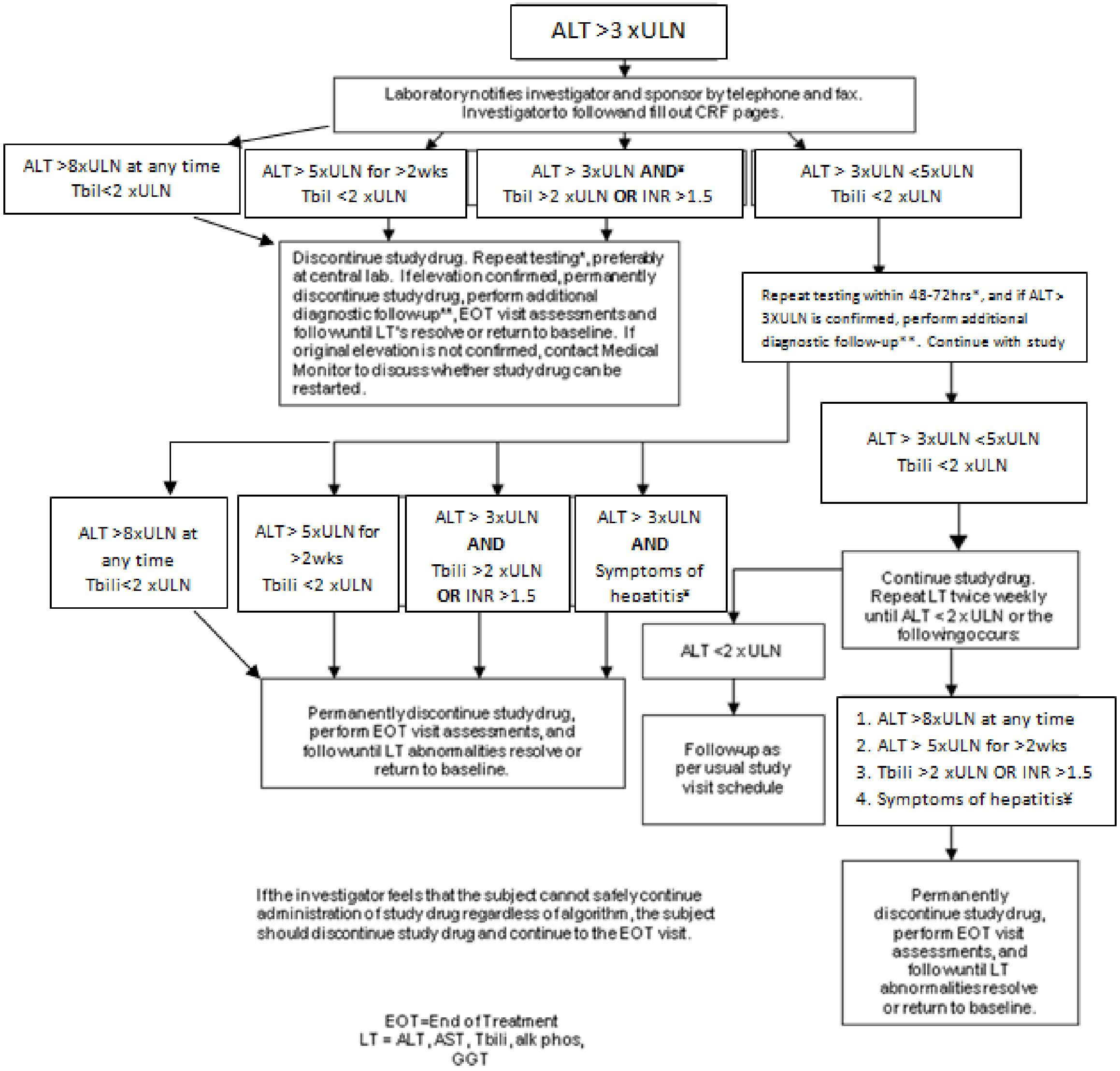

*Repeat testing within 48-72 hours in patients with initial ALT elevations, particularly if these are not events reported previously with the drug. If ALT transient elevations have been already established as part of the safety profile, the required frequency of retesting can be decreased

¥ OR ALT>3xULN with the appearance of fatigue, nausea, vomiting, right upper quadrant pain or tenderness, fever, rash, and/or eosinophilia (>5%)

****SEE NEXT PAGE FOR TESTS AND EVALUATIONS TO BE OBTAINED**

THE COMPLETE WORK-UP BELOW (ITEMS 1-5) SHOULD BE PERFORMED IN EVERY SITUATION WHERE "" APPEARS ABOVE. ITEMS 6-7 ARE OPTIONAL, TO BE CONSIDERED ON CASE BY CASE BASIS. ALL STUDIES SHOULD BE REPORTED WITH APPROPRIATE SOURCE DOCUMENTATION.**
THE STUDY MEDICAL MONITOR SHOULD BE NOTIFIED WHEN THE ABNORMALITIES ARE DETECTED AND PROVIDED WITH AN UPDATE OF THE RESULTS OF THE DIAGNOSTIC WORK-UP

The following definition of patterns of Drug Induced Liver Injury (DILI) is used when directing the work-up for potential DILI based on elevations of common laboratory tests (LT):

Histopathology	LT	Ratio (ALT/ULN)/(Alk Phos/ULN)
**Hepatocellular**	**ALT ≥ 3 × ULN**	**≥ 5**
**Cholestatic**	**ALT ≥ 3 × ULN**	**≤ 2**
**Mixed**	**ALT ≥ 3 × ULN and AP ≥ 2 × ULN**	**> 2 to < 5**

1. Obtain detailed history of present illness (abnormal LT's) including (if not already obtained at baseline) height, weight, body mass index (BMI). Assess for abdominal pain, nausea, vomiting, scleral icterus, jaundice, dark urine, pruritus, rash, fever, and lymphadenopathy. Assess for history of prior abnormal liver tests, liver disease including viral hepatitis, obesity, metabolic syndrome, congestive heart failure (CHF), occupational exposure to hepatotoxins, diabetes mellitus (DM), gallstone disease or family history of gallstone or liver disease. Specifically record history of alcohol use, other meds including acetaminophen, non-steroidal anti-inflammatory drugs (NSAID), over the counter (OTC) herbal supplements, vitamins, nutritional supplements, traditional Chinese medicines, and street drugs; and document whether or not there has been any recent change in any other prescription drugs and start-stop dates. Obtain travel history to endemic areas for hepatitis A, hepatitis E. Ask for history of any prior blood transfusions and when they were performed. Perform physical exam, obtain vital signs and BMI, and document presence or absence of scleral icterus, palpable liver including size, degree of firmness or tenderness, palpable spleen including size, ascites, and stigmata of chronic liver disease (spider angiomata, gynecomastia, palmar erythema, testicular atrophy). Allow free text in case report form for other relevant history and physical information.
2. Mandatory liver ultrasound with consideration of further imaging (eg, computerized tomography [CT], magnetic resonance imaging [MRI], magnetic resonance cholangiopancreatography (MRCP), endoscopic retrograde cholangiopancreatography (ERCP), Doppler studies of hepatic vessels, etc., if indicated based on ultrasound findings or clinical situation).
3. If total bilirubin (Tbili) is >2xULN, request fractionation to document the fraction that is direct bilirubin and to rule out indirect hyperbilirubinemia indicative of Gilbert's syndrome, hemolysis or other causes of indirect hyperbilirubinemia. Complete blood count (CBC) with

white blood count (WBC) and eosinophil count platelet count, international normalized ratio (INR), and total protein and albumin (compute globulin fraction) should also be documented. If INR is abnormal, prothrombin time (PT), partial thromboplastin time (PTT) should be obtained and these values should be followed until normal, along with documentation of whether parenteral vitamin K was given along with the effect of such treatment on INR.

4. If initial LTs and ultrasound do not suggest Gilbert's syndrome, biliary tract disease or obstruction, viral hepatitis serology should be obtained including anti-hepatitis A virus immunoglobin M (anti-HAV IgM), anti-HAV total, hepatitis B surface antigen (HBsAg), anti-HBs, anti-HB core total, anti-HB core IgM, anti-hepatitis C virus (anti-HCV), anti-hepatitis E virus IgM (anti-HEV IgM) (even if has not traveled to an endemic area for hepatitis E), Epstein-Barr virus (EBV) and Cytomegalovirus (CMV) screen.
   - If patient is immunosuppressed, test for HCV-RNA and HEV-RNA.
   - If HBsAg or anti-HB core IgM or anti-HB core IgG positive, also get HBV-DNA to detect active HepB, especially in patients who are immunosuppressed.
   - If all other hepatitis B serologic tests are negative and anti-HBc total is the only positive test, HBV-DNA should be obtained to detect reactivation of hepatitis B.
5. Assuming that the history, physical, and initial imaging and laboratory has not revealed a cause of elevated LTs, screen for other causes of liver disease including: Total protein and albumin (estimate globulin fraction and obtain quantitative immunoglobulins if elevated), antinuclear antibody (ANA), anti-liver kidney microsomal antibody type 1 (anti-LKM1), anti-liver-kidney microsomal antibodies (anti-LKM antibodies), anti-smooth muscle antibodies (ASMA), erythrocyte sedimentation rate (ESR), and C-reactive protein (CRP). If the pattern of laboratory abnormalities is not hepatocellular, but cholestatic or a mixed pattern (see definitions in table above), then gamma-glutamyl transferase (GGT), anti-mitochondrial antibody (AMA) and anti-neutrophil cytoplasmic antibody (pANCA) should also be tested. If there is an indication by history or elevated baseline LTs that there may be an underlying chronic liver disease possibly exacerbated by exposure to the study intervention in the clinical trial or making the participant more susceptible to DILI, test iron/Total iron binding capacity (TIBC) and ferritin (hemochromatosis), and alpha-1-antitrypsin level. If patient is <50 years of age, ceruloplasmin should also be tested to screen for Wilson's disease. If patient is sick enough to be hospitalized and is under age 50, a slit lamp examination to detect Kayser-Fleischer rings and a 24-hour urine collection for copper should be measured. Consider serum ethanol and/or acetaminophen level and urine drug screen as clinically appropriate.
6. A liver biopsy should be considered if autoimmune hepatitis remains a competing etiology and if immunosuppressive therapy is contemplated.

   A liver biopsy may be considered:

   - if there is unrelenting rise in liver biochemistries or signs of worsening liver function despite stopping the suspected offending agent.
   - if peak ALT level has not fallen by >50% at 30-60 days after onset in cases of hepatocellular DILI, or if peak Alk P has not fallen by >50% at 180 days in cases of cholestatic DILI despite stopping the suspected offending agent.
   - in cases of DILI where continued use or re-exposure to the implicated agent is expected.

– if liver biochemistry abnormalities persist beyond 180 days to evaluate for the presence of chronic liver diseases and chronic DILI.

7. If pertinent, copies of hospital discharge summary, radiology, pathology and autopsy reports should be obtained.

Abbreviations

AlkP	alkaline phosphatase
ALT	alanine aminotransferase
AMA	anti-mitochondrial antibody
ANA	antinuclear antibody
Anti-LKM1	anti-liver kidney microsomal antibody type 1
ASMA	anti-smooth muscle antibodies
AST	aspartate aminotransferase
BMI	body mass index
CBC	complete blood count
CHF	congestive heart failure
CMV	cytomegalovirus
CRP	C-reactive protein
CT	computerized tomography
DM	diabetes mellitus
DNA	deoxyribonucleic acid
EBV	Epstein-Barr virus
ERCP	endoscopic retrograde cholangiopancreatography
ESR	erythrocyte sedimentation rate
EOI	end of intervention
GGT	gamma-glutamyltransferase
HAV	hepatitis A virus
HBsAg	hepatitis B surface antigen
HCV	hepatitis C virus
HepB	hepatitis B virus
HEV	hepatitis E virus
IgM	immunoglobin M
INR	international normalized ratio
LT/LFT	liver tests/liver function tests
MRI	magnetic resonance imaging
MRCP	magnetic resonance cholaniopancreatography
NSAID	non-steroidal anti-inflammatory drugs
OTC	over the counter
PT	prothrombin time
PTT	partial thromboplastin time
RNA	ribonucleic acid
Tbili	total bilirubin
TIBC	total iron binding capacity
ULN	upper limit of normal
WBC	white blood count

10.5. Appendix 5: ECOG-PS Score

ECOG Grade Scale (with Karnofsky conversion)

0	Fully active, able to carry on all pre-disease performance without restriction. (Karnofsky 90-100)
1	Restricted in physically strenuous activity but ambulatory and able to carry out work on a light or sedentary nature, eg. light housework, office work. (Karnofsky 70-80)
2	Ambulatory and capable of all self-care but unable to carry out any work activities. Up and about more than 50% of waking hours. (Karnofsky 50-60)
3	Capable of only limited self-care; confined to bed or chair more than 50% of waking hours. (Karnofsky 30-40)
4	Completely disabled. Cannot carry on any self-care. Totally confined to bed or chair. (Karnofsky 10-20)
5	Dead. (Karnofsky 0)

10.6. Appendix 6: Protocol Amendment History

Amendment 1 (7 June 2019)

Overall Rationale for the Amendment: The overall reason for the amendment is to extend the time cutoff for the earliest prostate-specific antigen (PSA) values used in the calculation of PSA doubling time (PSADT) to allow patients who may have had one or more of the required PSAs collected outside the previously defined 6 months window who are otherwise eligible. Other changes include clarification of the way of ADT provided and used, and the window for planned visit and imaging.

Section Number and Name	Description of Change	Brief Rationale
1.1 Synopsis: OBJECTIVES AND ENDPOINTS; 3 OBJECTIVES AND ENDPOINTS: footnote "a"	Specified that subjects randomized to placebo arm will be crossed over to receive apalutamide treatment either after completion of a maximum 5 cycles of placebo treatment or at the time of having documented PSA progression, whichever comes earlier.	To clarify the expected cross-over time for subjects of placebo arm in consistency with other sections of the protocol.
1.1 Synopsis: OVERALL DESIGN; 4.1 Overall design	Specified that subjects will be stratified by PSA doubling time (PSADT) (>6 months vs ≤6 months).	In original protocol, patients will be stratified according to PSADT (>6 months vs ≤6 months) at randomization. However, this information was missed in a few places. Added specification in consistency with other sections of the protocol.
1.1 Synopsis: INTERVENTION GROUPS AND DURATION; 6.1 Study interventions administered	Table: Changed "30-ct, 100 cc" to "120-ct, 160 cc"	To clarify and correct information
1.1 Synopsis: INTERVENTION GROUPS AND DURATION; 2.2.1 Androgen Deprivation Therapy; 6.1 Study interventions administered	Added specification that ADT will be provided by sponsor and clarity ADT with GnRHa will be continued for all patients who have not been surgically castrated in consistency with other sections of the protocol.	To specify the way of ADT provided and used.
1.1 Synopsis: EFFICACY EVALUATIONS; 4.1 Overall design	Added specification that PSA evaluations will be performed by a central lab "during the treatment phase".	For patients participating the optional prescreening phase, PSA evaluations will be performed by the local laboratories. Added the specification that PSA will be performed by a central lab during the treatment phase to avoid confusion.
1.1 Synopsis: EFFICACY EVALUATIONS; 1.3 Schedule of Activities (SoA): footnote 9; 8.1 Efficacy Assessments	Added "intravenous (IV) contrast is required when not medically contraindicated" for disease assessment.	To clarify and add requirements for disease assessment
1.3 Schedule of Activities (SoA)	Updated information for apalutamide/matched placebo administration	In the original protocol, only daily dosage was instructed in the SoA. Updated the table to reflect the study drug dispense.
1.3 Schedule of Activities	Added specification that during the	In the original protocol, window

**Section Number and Name**	**Description of Change**	**Brief Rationale**
(SoA)<br>8 STUDY ASSESSMENTS AND PROCEDURES: Overview	Treatment Phase, study visits may occur within ± 3-day window.<br>Added specification that imaging may occur up to 8 days before cycles requiring images and that unscheduled assessments can occur at any time as needed if signs of disease progression are observed.	allowed for planned visit and imaging was not specified. This amendment added the information that a ±3-day window is allowed for planned visit during the treatment phase and planned imaging may occur up to 8 days before cycles requiring imaging.
1.3 Schedule of Activities (SoA)	Added time window allowed for PK sample collection: predose within 4.8h prior to dosing; 2h postdose (± 10 minutes).	To add the time window allowed in consistency with SPARTAN and Protocol 56021927PCR1013.
1.3 Schedule of Activities (SoA)	Added specification that the SAE reporting would be limited to SAEs related to the PSA blood draws during the prescreening phase.	To clarify the SAE collection during the optional prescreening phase
4.4 End of study definition	Deleted "in all participating countries".	The study is only in China.
6.2 Preparation/Handling/Storage/Accountability	The temperature range for storage of study intervention was changed from "20°C to 25°C" to "15°C to 30°C"	Information correction
6.3 Procedures for Randomization	Updated the window allowed for PSA values for PSADT calculation from within 6 months prior to randomization to within 24 months prior to randomization and specified that the first and last PSA values used in the calculation must be separated by at least 8 weeks.	The window of requiring PSA values collected within 6 months prior to randomization for calculation of the PSADT has the unintended consequence of excluding patients who are otherwise eligible, but who may have had one or more of the required PSAs collected outside the 6 months window. Therefore, this amendment will incorporate a cutoff that allows inclusion of PSA values collected within 24 months prior to the subject's randomization.
6.6 Dose Modification	Clarified that for toxicities other than rash, "The investigator's rationale to re-escalate treatment must be discussed with and approved by the sponsor's medical monitor on an individual basis prior to implementation."	Dose re-escalation for rash is allowed for apalutamide/placebo at the discretion of the investigator. This revision is to clarify that for toxicities other than rash, dose re-escalation is generally not allowed unless being discussed with and approved by the Sponsor.
8.2.1Physical Examination	Added that weight assessment will be conducted as part of physical examination after screening.	Weight decreased has been reported in patients receiving apalutamide treatment. Thus, added weight assessment to have patients' weight monitored.
8.2.4 Clinical Safety Laboratory Assessment	Added specification that unscheduled laboratory assessment may be performed locally when an immediate clinical decision needs to be made.	Per investigator's feedback, this is to add specification that local lab can be used due to urgent safety consideration.
Throughout the protocol	Minor grammatical, formatting, or spelling changes were made.	Minor errors were noted

Amendment 2 (20 February 2020)

Overall Rationale for the Amendment: The overall reason for the amendment is to 1) clarify and edit the exclusion criteria and SPARTAN study result in Study-Specific Ethical Design Considerations, 2) and clarify specific information including efficacy evaluations, androgen deprivation therapy and Clinical Safety Laboratory Assessments.

Section Number and Name	Description of Change	Brief Rationale
1.1 Synopsis: EFFICACY EVALUATIONS<br>1.3 Schedule of Activities (SoA)<br>8.1 Efficacy Assessments	Added statements for efficacy evaluations: "scans obtained prior to signing informed consent as part of the patient's standard of care may be allowed, provided the timing of the scans fall within the screening window"; "The same method of assessment and the same technique should be used at Screening and during follow-up." And "Tumor evaluation by positron emission tomography (PET) scan or by ultrasound or by prostate-specific membrane antigen (PSMA)-PET will not replace for standard chest, abdomen, and pelvis CT or MRI scans or bone scans as required by the study, but the CT portion of a PET/CT with sufficient quality which could meet the study requirement may be submitted in lieu of a dedicated CT."	To clarify the scan collection requirement.
1.1 Synopsis: OVERALL DESIGN<br>1.3 Schedule of Activities (SoA)-Footnote 3<br>4.1 Overall Design<br>7.1 Discontinuation of Study Intervention	• Specified the study visit window for planned Posttreatment Follow-up visit after documented disease progression (distant metastases) to be ±7 days.<br>• Updated the wordings related to Posttreatment Follow-up phase.	• In the original protocol, window allowed for planned Posttreatment Follow-up visit after documented disease progression (distant metastases) was not specified.<br>• Clarify the Posttreatment Follow-up process for participants entering Posttreatment Follow-up phase with or without evidence of distant metastasis.
1.3 Schedule of Activities (SoA)	Added comments for physical examination "The screening physical examination will also include a full review of medical or oncological history and a complete examination of all organ systems. Limited symptom directed physical examinations and weight assessment will be conducted at other visits." and vital signs "Body temperature, pulse/heart rate, respiratory rate, and BP are measured at the screening. Only BP is recorded at other visits.".	To clarify the different requirements for physical examination and vital signs at screening visit and other visits.
1.3 Schedule of Activities (SoA)-Footnote 2<br>8 STUDY ASSESSMENTS AND PROCEDURES	Specified the study visit window to be -3 ~ +2-day	Update the study visit window to avoid missing dose.
2.2.1 Androgen	Specified "Androgen Deprivation	To further clarify androgen deprivation

Section Number and Name	Description of Change	Brief Rationale
Deprivation Therapy	Therapy with continuous GnRHa will be continued for all patients who have not been surgically castrated.”	therapy that either continuous GnRHa or surgically castration is acceptable.
4.2.1 Study-Specific Ethical Design Considerations	Previous statement “Further, on the SPARTAN study only 1 placebo subject (out of 401 enrolled) was found to have metastatic disease at the first post-baseline scan without prior to PSA progression.” was revised to be “Further, on the SPARTAN study about 6.2% of the placebo participants were found to have metastatic disease at the first post-baseline scan without prior PSA progression and only 2-3 participants in this study are expected to have the same situation given one third of 111 Chinese participants (about 37 subjects) are to be randomly assigned to placebo arm.”	To correct the information.
5.2 Exclusion Criteria No 15	Corrected exclusion criteria 15 from “For men who are sexually active with women of child bearing potential” to “Men who are sexually active with women of childbearing potential except”	To clarify the typo in exclusion criteria 15
5.2 Exclusion Criteria No 13	Exclusion criteria 13 was divided into two: 13 and 18.	Error correction
1.1 Synopsis: OVERALL DESIGN<br>4.1 Overall Design<br>6.3 Measures to Minimize Bias: Randomization and Blinding: Blinding	Revised “The PSA data collected at baseline and post-baseline obtained within the first 5 cycles, for individual participant will be analyzed by the central laboratory to flag out documented PSA progression per PCWG2. The data will be integrated into the IWRS to trigger the cross-over.” to be “The central laboratory will analyze the individual PSA data, collected at baseline and post-baseline obtained within the first 5 cycles to flag out documented PSA progression per PCWG2 and the data will be entered into the IWRS to trigger the cross-over by an independent team not affiliated with the study team. The cross-over of control arm participants will be handled by IWRS to maintain the blinding of original treatment assignment of individual participant.”	To clarify the original text and specify that an independent team not affiliated with the study team will trigger the cross-over.
6.6.1 Dose Modifications for Rash and Rash Management	Revised and corrected the information to be “Apalutamide/placebo can be held up to 28 days in the event of rash. If treatment interruption for any AE grade is longer than 28 days, resumption of treatment may still be feasible. Please reach out to the sponsor to discuss	Update and align the information at different location.

Section Number and Name	Description of Change	Brief Rationale
	further management. Dose re-escalation for rash is allowed for apalutamide /placebo at the discretion of the investigator.”	
8.2.4 Clinical Safety Laboratory Assessments	Clarified “total T3, free T4 (direct) and Total T4 if TSH is not within normal limit” from “T3, T4 (direct) and Total T4 if TSH is not within normal limit”.	To clarify the information.
Throughout the protocol	Minor grammatical, formatting, or spelling changes were made.	Minor errors were noted

11. REFERENCES

1. Chen W, Zheng R, Baade PD, et al. Cancer Statistics in China. 2015. CA Cancer J Clin. 2016;66:115-132.
2. Clinical Study Protocol ARN-509-003 (SPARTAN). A Multicenter, Randomized, Double-Blind, Placebo-Controlled, Phase III Study of ARN509 in Men with Non-Metastatic (M0) Castration-Resistant Prostate Cancer. Version 3.0. Janssen Research & Development, LLC. (08 May 2013).
3. Clinical Study Report ARN-509-001. An open-label, phase I/II safety, PK, and proof-of-concept study of ARN-509 in patients with progressive advanced castration-resistant prostate cancer. Janssen Research & Development, LLC. (25 Aug 2017).
4. Clinical Study Report ARN-509-003 (SPARTAN). A multicenter, randomized, double-blind, placebo-controlled, phase III study of ARN-509 in men with non-metastatic (M0) castration-resistant prostate cancer. Janssen Research & Development, LLC. (25 Sep 2017).
5. Clinical Study Report Addendum ARN-509-003 (SPARTAN)—Update to the Original Clinical Study Report dated 25 September 2017. Final Analysis of Overall Survival, Efficacy, and Safety. (30 April 2020)
6. Freedland SJ, Humphreys EB, Mangold LA, et al. Death in patients with recurrent prostate cancer after radical prostatectomy: prostate-specific antigen doubling time subgroups and their associated contributions to all-cause mortality. J Clin Oncol. 2007;25:1765-1771.
7. Investigator's Brochure: JNJ-56021927 (apalutamide), edition 16. Janssen Research & Development, LLC. (14 Apr 2022).
8. Rathkopf DE, Morris MJ, Fox JJ, et al. Phase I study of ARN-509, a novel antiandrogen, in the treatment of castration-resistant prostate cancer. J Clin Oncol. 2013;31:3525-3530.
9. Smith MR, Cook R, Lee KA, Nelson JB. Disease and host characteristics as predictors of time to first bone metastasis and death in men with progressive castration-resistant nonmetastatic prostate cancer. Cancer 2011;117:2077-2085.
10. Smith MR, Kabbinavar F, Saad F, et al. Natural history of rising serum prostate-specific antigen in men with castrate nonmetastatic prostate cancer. J Clin Oncol. 2005;23:2918-2925.
11. Stephenson AJ, Kattan MW, Eastham JA, et al. Defining biochemical recurrence of prostate cancer after radical prostatectomy: a proposal for a standardized definition. J Clin Oncol. 2005;23:8253-8261.

INVESTIGATOR AGREEMENT

I have read this protocol and agree that it contains all necessary details for carrying out this study. I will conduct the study as outlined herein and will complete the study within the time designated.

I will provide copies of the protocol and all pertinent information to all individuals responsible to me who assist in the conduct of this study. I will discuss this material with them to ensure that they are fully informed regarding the study intervention, the conduct of the study, and the obligations of confidentiality.

Coordinating Investigator (where required):

Name (typed or printed): ______________________

Institution and Address: ______________________

____

____

____

Signature: ______________________ Date: ______________________
(Day Month Year)

Principal (Site) Investigator:

Name (typed or printed): ______________________

Institution and Address: ______________________

____

____

____

Telephone Number: ______________________

Signature: ______________________ Date: ______________________
(Day Month Year)

Sponsor's Responsible Medical Officer:

Name (typed or printed): Xiao, Chang (Cathy)

Institution: Janssen Research & Development, [LLC]

Signature: [signature] Date: 23 Jun 2022
(Day Month Year)

Note: If the address or telephone number of the investigator changes during the study, written notification will be provided by the investigator to the sponsor, and a protocol amendment will not be required.
